# Extensive Phylogenetic Analysis of Piscine Orthoreovirus Genomic Sequences Shows the Robustness of Subgenotype Classification

**DOI:** 10.3390/pathogens10010041

**Published:** 2021-01-07

**Authors:** Marcos Godoy, Daniel A. Medina, Rudy Suarez, Sandro Valenzuela, Jaime Romero, Molly Kibenge, Yingwei Wang, Frederick Kibenge

**Affiliations:** 1 Laboratorio de Biotecnología Aplicadas, Facultad de Medicina Veterinaria, Universidad San Sebastián, Sede De la Patagonia, Lago Panguipulli 1390, Puerto Montt 5480000, Chile; marcos.godoy@uss.cl (M.G.); daniel.medina@uss.cl (D.A.M.); 2 Centro de Investigaciones Biológicas Aplicadas (CIBA), Lago Panguipulli 1390, Puerto Montt 5480000, Chile; rudy.suarez@ciba.cl (R.S.); sandrolvalenzuelad@gmail.com (S.V.); 3 Doctorado en Acuicultura, Programa Cooperativo Universidad de Chile, Universidad CatÓlica del Norte, Coquimbo 1781421, Chile; 4 Universidad CatÓlica del Norte, Coquimbo 1781421, Chile; 5 Laboratorio de Biotecnología, Instituto de Nutrición y Tecnología de los Alimentos, Universidad de Chile, Santiago 7800024, Chile; jromero@inta.uchile.cl; 6 Department of Pathology and Microbiology, Atlantic Veterinary College, University of Prince Edward Island, Charlottetown, PE C1A4P3, Canada; mkibenge@upei.ca; 7 School of Mathematical and Computational Sciences, University of Prince Edward Island, Charlottetown, PE C1A4P3, Canada; ywang@upei.ca

**Keywords:** *Piscine orthoreovirus*, PRV, genotype, subgenotype, phylogeny

## Abstract

*Piscine orthoreovirus* (PRV) belongs to the family *Reoviridae* and has been described mainly in association with salmonid infections. The genome of PRV consists of about 23,600 bp, with 10 segments of double-stranded RNA, classified as small (S1 to S4), medium (M1, M2 and M3) and large (L1, L2 and L3); these range approximately from 1000 bp (segment S4) to 4000 bp (segment L1). How the genetic variation among PRV strains affects the virulence for salmonids is still poorly understood. The aim of this study was to describe the molecular phylogeny of PRV based on an extensive sequence analysis of the S1 and M2 segments of PRV available in the GenBank database to date (May 2020). The analysis was extended to include new PRV sequences for S1 and M2 segments. In addition, subgenotype classifications were assigned to previously published unclassified sequences. It was concluded that the phylogenetic trees are consistent with the original classification using the PRV genomic segment S1, which differentiates PRV into two major genotypes, I and II, and each of these into two subgenotypes, designated as Ia and Ib, and IIa and IIb, respectively. Moreover, some clusters of country- and host-specific PRV subgenotypes were observed in the subset of sequences used. This work strengthens the subgenotype classification of PRV based on the S1 segment and can be used to enhance research on the virulence of PRV.

## 1. Introduction

Heart and skeletal muscle inflammation (HSMI) is an infectious disease caused by Piscine orthoreovirus (PRV) and was described for the first time in Norway [1,2,3]. PRV belongs to the genus *Orthoreovirus* [4], subfamily *Spinareovirinae* in the *Reoviridae* family [1].

PRV has been detected in numerous wild and farmed salmonid species in Norway [5,6,7,8,9], Canada [10,11,12,13,14], Chile [10,15], the United States [12,16], Japan [17], Scotland, Ireland, the Faroe Islands, Iceland, Germany, Sweden, Denmark, Italy, France [18,19,20,21,22,23] and the Czech Republic [24].

In Atlantic salmon (*Salmo salar*) and Coho salmon (*Oncorhynchus kisutch*) in Chile the clinical signs of affected fish are mainly characterized by systemic circulatory disturbance. Histopathologically, the fish present myocarditis and myositis of the red musculature [2,15,25,26]. Moreover, during PRV infection, the presence of intracytoplasmic inclusion bodies in erythrocytes has been described [26,27,28]. 

The genome of PRV consists of about 23,600 bp, which encode at least 11 proteins distributed across ten segments of double-stranded RNA, classified as small (S1, S2, S3 and S4), medium (M1, M2 and M3) and large (L1, L2 and L3); these range approximately from 1000 bp (segment S4) to 4000 bp (segment L1) [1,4,10]. Phylogenetic analysis, based mainly on the PRV S1 segment, differentiates the virus into two genotypes, I and II, and each of these into two major subgenotypes, designated as Ia and Ib, and IIa and IIb, respectively [10,15]. Alternatively, Garseth et al. [29] described four genogroups, I, II, III and IV. A complementary classification using the coding sequence of PRV segments described three subtypes of PRV, designated as PRV-1, PRV-2 and PRV-3 [17,19], with subgenotypes Ia and Ib making up the PRV-1 subtype and subgenotypes IIb and IIa corresponding to the PRV-2 and PRV-3 subtypes, respectively [30]. 

PRV subgenotypes Ia and Ib have been associated with HSMI in Atlantic salmon and HSMI-like disease in Rainbow trout (*Oncorhynchus mykiss*) and Coho salmon [1,3,7,13,15]. In Canada, the infection of Chinook salmon (*O. tshawytscha*) with PRV subgenotype Ia is associated with Jaundice disease [14,31] and in Japan, infection with the PRV-2 (subgenotype IIb) causes erythrocytic inclusion body syndrome (EIBS) [17]. No PRV subgenotype or subtype has been described to be host-specific or geographically exclusive, apart from PRV-Ib, which has not been found in Canada or the USA to date [10,32], and PRV-2, which to date has been reported only in Japan [17]. Consequently, the wide range of hosts, clinical signs and geographic distribution have turned PRV into an emerging virus in the salmon aquaculture industry worldwide.

The aim of this study was to revisit the molecular classification, based on phylogenetic analysis, of almost all PRV sequences available to date (May 2020) in the GenBank database, with a focus on the S1 and M2 genome segments, including unpublished new sequences generated for this work. Our results strengthen the subgenotype classification of PRV based on the S1 segment and allow the classification of previously unclassified, publicly available sequences into the PRV subgenotypes through phylogenetic dendrograms.

## 2. Results

### Phylogenetic Analyses of PRV Genomic Sequences

We retrieved all the available sequences for the ten genomic segments of PRV listed under the Tax ID: txid1157337 in the National Center for Biotechnology Information (NCBI) GenBank database (Supplementary Table S1). New sequences for the S1 and M2 segments were added in preparation for this work, resulting in the obtainment of between 38 and 390 sequences with respect to the PRV segments used in the study (Table 1).

**Figure 1:**
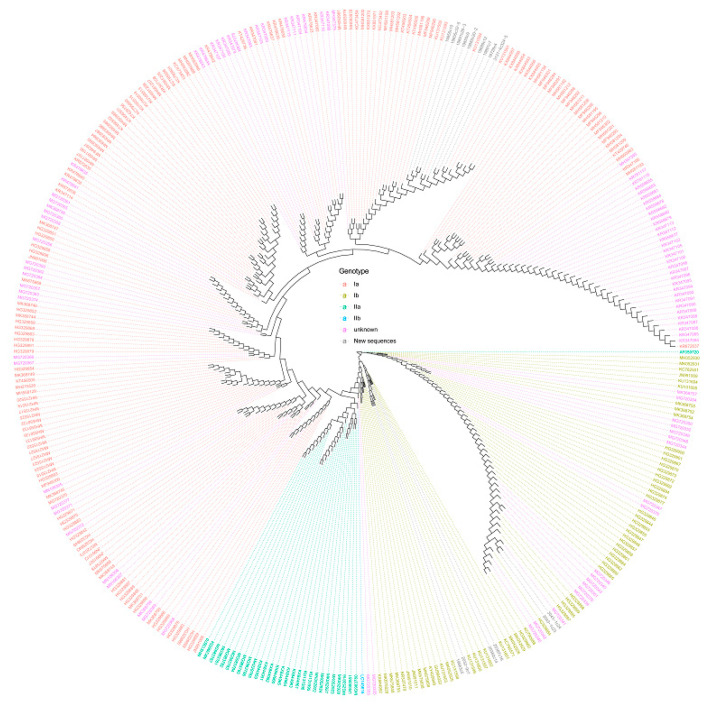
Circular dendrogram of S1 PRV genomic segment. The phylogenetic analysis was performed using sequences with at least 400 nucleotides. New and unknown subgenotype sequences were assigned according to their positions in the dendrogram branches.

**Figure 2:**
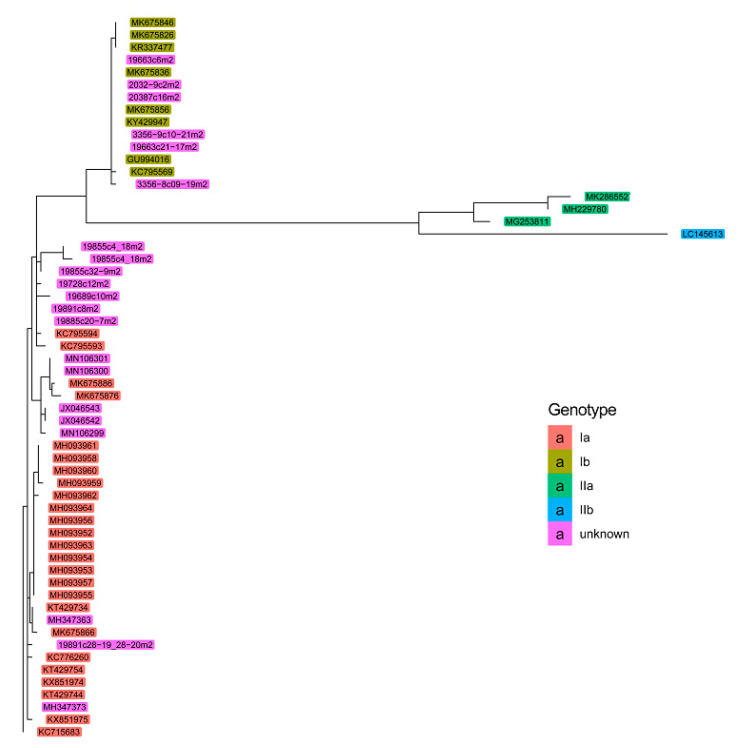
Phylogenetic tree of PRV M2 genomic segment. The dendrogram was built utilizing sequences with at least 1000 nucleotides. New and unknown subgenotype sequences were assigned according to their positions in the dendrogram branches.

The sequences collected were used to perform an initial phylogenic analysis based on subgenotype classifications, obtaining a clear differentiation using PRV S1 and M2 segments. In accordance with previous observations, it was concluded that the use of these segments would enable the grouping of the PRV into subgenotypes [10,15]. We performed a restrictive analysis of the S1 and M2 segments, discarding sequences with less than 400 nucleotides for the S1 segment and less than 1000 nucleotides for the M2 segment, to cover the conserved coding sequences of σ3 and μ1 proteins. A total of 356 and 59 sequences, respectively, were retained in the end, including the 11 new sequences each for S1 and M2 segments reported in this work (Table 2). The circular dendrogram of the PRV S1 sequences is presented in Figure 1, showing the assignment of new and published unclassified sequences according to their positions in the dendrogram branches. A rectangular version of Figure 1 is provided to enable better exploration of the data (Supplementary Figure S1). The phylogenetic tree of PRV M2 sequences is presented in Figure 2, showing the assignment of new and published unclassified sequences according to their positions in the dendrogram branches. These phylogenetic analyses were able to group the PRV genogroups and allowed us to classify both new sequences (Table 2) and published unclassified sequences into the PRV subgenotypes (Supplementary Table S2). The remaining eight genomic segments had lower resolution and representativity and did not allow for a clear ordination using either subgenotype [10] or subtype [20] classifications (Supplementary Figure S2).

**Figure 3:**
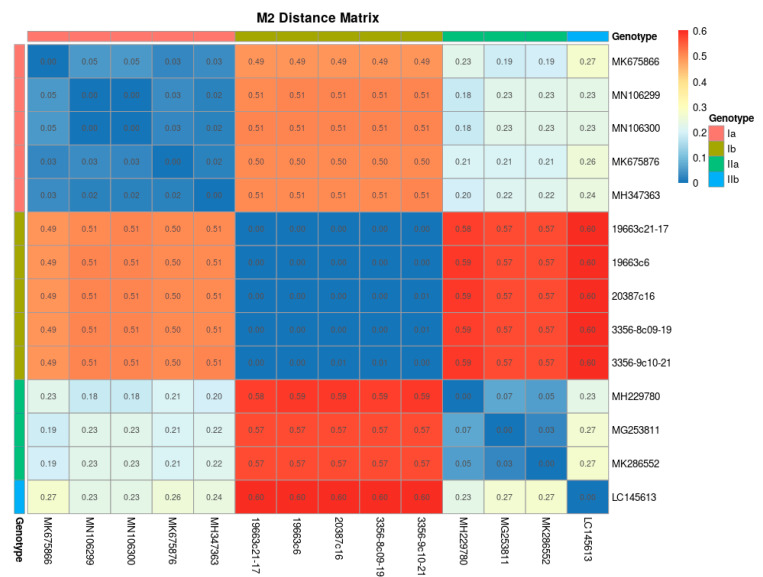
Phylogenetic distance heatmap for 14 PRV M2 genomic sequences. The colors represent the subgenotype and the distance matrix value.

**Figure 4:**
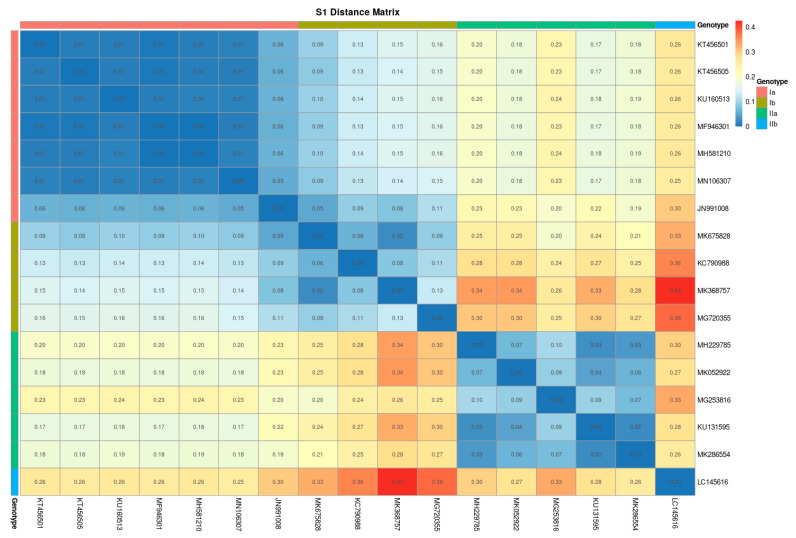
Phylogenetic distance heatmap obtained from 17 PRV S1 genomic sequences. The colors represent the subgenotype and the distance matrix value.3. Discussion.

We then used 14 and 17 genomic sequences belonging to M2 and S1 segments, respectively, to graphically show the distances between the sequences according to their classification by subgenotype. Both genomic distances matrixes obtained show good fitness in differentiating between the PRV subgenotypes (Figure 3 and Figure 4). Then, we extended this analysis using 40 and 61 amino acidic sequences belonging to M2 and S1 segments that encode the proteins μ1 and σ3, respectively, including the new sequences produced in this work, classified by subgenotype, subtype, country and host. The distance matrix built using the amino acid sequences of the protein μ1 (M2 segment), presented in Supplementary Figure S3, shows less robustness in the heatmaps used to cluster PRV by subgenotype or subtype. In contrast, the protein σ3 heatmaps show better clustering using subgenotype and subtype classifications (Supplementary Figure S4). Thus, two clear clusters belonging to subgenotypes Ia and Ib were observed in the PRV-1 subtype; these served to strengthen this subtype classification (Figure 4 and Supplementary Figure S3). Country (Supplementary Figure S5) and host species (Supplementary Figure S6) clustering were observed when heatmaps were drawn using these metadata variables. We observed, mainly with the protein σ3 (S1 segment), that the Chile, Norway and Canada sequences shared low distance, suggesting high similarity. However, in the Chile sequences there were two marked groups, one sharing low distance sequences with Canada and Norway and the other clustering with a few Italy and Denmark sequences (Supplementary Figure S5). This agrees with previous reports of the presence of two subgenotypes in Chile [10,15]. Regarding the host species, the distance matrixes inside the Coho salmon sequences were found to have two clear clusters, thereby indicating that this host may carry two PRV subgenotypes. In addition, Coho salmon was found to share low distance clusters with Rainbow trout and a few sequences belonging to Atlantic salmon, suggesting that they harbor similar PRV subgenotypes. We found similar observations in the case of Atlantic salmon. First, we observed two clusters of low distance, suggesting that Atlantic salmon harbors two kinds of PRV sequences. Moreover, Atlantic salmon shared low distance clustering with Chinook salmon and a few sequences of Rainbow trout and Coho salmon (Supplementary Figure S6).

## 3. Discussion

We extended the geographical range of the already characterized PRV isolates by compiling all the available sequences to date (May 2020) associated with the NCBI Taxonomy ID: 1157337 of PRV. Moreover, we added new sequences for the S1 and M2 segments of 11 PRV isolates from Chile, where salmonids are non-native species and where Coho salmon, Rainbow trout and Atlantic salmon are farmed in the same regions, which contributes to the understanding of the phylogenetics of PRV. This study supports the original classification using the PRV genomic segment S1, which differentiates PRV into two major genotypes, I and II, and each of them into two subgenotypes designated as Ia and Ib, and IIa and IIb, respectively [10,15]. Through massive phylogenetic dendrograms with all available sequences, we provided additional evidence that strengthens this classification using new PRV S1 and M2 segment sequences. These dendrograms allowed us to classify, by subgenotype, already published sequences of S1 and M2 segments that were previously unclassified in the GenBank database. This assignment showed that a significant number of the publicly available sequences belong to the PRV-1 subtype (subgenotypes Ia and Ib) and, to a lesser extent, to the PRV-3 subtype (subgenotype IIa). However, the lack of sequences of PRV-2 (subgenotype IIb) points to the need to perform further analysis to understand the contribution of this variant to the worldwide PRV phylogeny. Furthermore, this kind of study should be complemented by using methodologies based on whole genome sequencing.

The distance analyses of the PRV genomic M2 and S1 segments and the amino acid sequences of proteins σ3 (S1 segment) and μ1 (M2 segment) show clear clustering between sequences belonging to the same subgenotype. This contrasts with the PRV-1 subtype classification [20], which shows, in protein σ3 alignments, two marked clusters belonging to subgenotypes Ia and Ib, thereby strengthening the subgenotype classification. Although no country- or host-specific PRV subgenotype has been described, some clusters were observed in the subset of sequences used. These latter observations indicate the need to perform a deep meta-analysis to elucidate whether relationships between virulence and subgenotypes can be described. In fact, the PRV subgenotypes Ia and Ib (PRV-1) have been associated with HSMI in Atlantic salmon, this being a trending topic in aquaculture surveillance. In farmed Atlantic salmon, PRV-Ib has been shown to be the cause of HSMI [3]—whereas experimental studies have shown PRV-Ia to be of low virulence, although it is associated with moderate heart lesions in sockeye salmon [31]—and in Pacific Canada the associated disease in farmed Atlantic salmon was referred to as HSMI-like disease [33]. The higher virulence of PRV-Ib was linked to the evolution of PRV in Norwegian salmonid aquaculture possibly resulting from gene reassortment involving PRV segments S1 and M2 [34]. This prompted the development of a real-time reverse transcription polymerase chain reaction (RT-qPCR) assay specific for PRV-Ib associated with HSMI [35]. However, the study linking PRV-Ib to higher virulence [34] was largely speculative because, although eight of the PRV segment S1 sequences used in that study were of PRV-Ia from HSMI in BC Canada [13,14], the so-called “HSMI Clade” included only PRV-Ib isolates. Moreover, some of the PRV-Ib isolates have not been associated with HSMI [6,36]. In fact, an experimental study comparing the virulence of three PRV-1b isolates and three PRV-1a isolates in farmed Atlantic salmon showed no differences in PRV RNA in blood cells among the different isolates when measured by RT-qPCR from one to ten weeks post-challenge. However, two of the PRV-1b isolates resulted in more severe heart histopathology scores than the other PRV-1b isolate and the three PRV-1a isolates, although even in these PRV isolates with less severe histopathological lesions, a few individual fish were observed to have lesion scores high enough to qualify as HSMI [37]. It therefore follows that the variation in virulence found in PRV-1b isolates would be expected to also occur among PRV-1a isolates. The identification of the sequence relationships and the subgenotype classification performed in the present study can be used to enhance research on the virulence of PRV. 

## 4. Materials and Methods

### 4.1. Extensive Phylogeny of PRV Segments 

Sequences in FAST-All (FASTA) format for all PRV segments were retrieved from the NCBI GenBank public database [38]. Sequences from 2007 to the present date (May 2020) were selected by means of the rentrez package [39] under R environment v3.5.2 [40], utilizing the NCBI Taxonomy ID: txid1157337 assigned to PRV. To increase the accuracy of our analysis, multiple sequence alignments (MSAs) [41] were performed using the T-Coffee software [42]. Additionally, IQ-TREE, software to calculate trees using the maximum likelihood algorithm [43], was used to find the best model substitution automatically through its ModelFinder feature [44]. Trees were built using the ggtree [45] and ggplot2 packages [46], both based on the R statistical language [40] and scripted utilizing the integrated development environment R-Studio [47]. To graphically show the distance between σ3 and μ1 amino acidic sequences, the nucleotide sequences were translated to amino acidic sequences using Blastx [48]. Heatmaps were drawn using the distance matrix calculated by the phangorn R library [49] from the alignments obtained by the maximum likelihood algorithm using the Clustal Omega software, including the model substitution parameter [50]. The resulting distance information was postprocessed and formatted using the library reshape2 [51] and represented using the pheatmap package [52] under the R statistical language.

### 4.2. Nucleic Acid Sequencing of New Isolates

Viral sequencing was performed following the methods previously described to sequence genomic PRV segments [10,15]. Briefly, the RNA was isolated using a modified extraction protocol based on equilibrated phenol and stabilized chloroform, isoamyl alcohol (25:24:1) (PanReac Applichem, Chicago, IL, USA), and repurified using the E.Z.N.A.^®^ Total RNA Kit I ( Omega Bio-tek, Inc., Norcross, GA, USA). The RNA was tested by RT-qPCR to detect the presence of PRV, using ELF-1α as internal control, following the protocol described by Kibenge et al. [10]. Only samples with a cycle threshold (Ct) below 25 were utilized to perform the DNA sequencing of segments S1 and M2 using the primers described in Kibenge et al. [10]. Sequences were deposited in the NCBI GenBank database under the accession numbers listed in Table 2.

## Figures and Tables

**Table 1:** Number of sequences for each Piscine orthoreovirus (PRV) genomic segment used in the study.

	PRV Genomic Segment									
	L1	L2	L3	M1	M2	M3	S1	S2	S3	S4
Number of sequences	53	38	40	41	61	43	390	101	42	100

**Table 2:** New sequences of PRV segments S1 and M2 produced in this study.

GenBank Accession Number	PRV Segment	PRV Subgenotype	Collection Date	Fish Species ^1^	Location in Chile	Farming Conditions
MT598071	M2	Ib	06-04-2018	*O. kisutch*	Chiloé (Calen)	Seawater
MT598072	M2	Ia	12-04-2018	*S. salar*	Chiloé (Quellón)	Seawater
MT598073	M2	Ia	26-04-2018	*O. kisutch*	Chiloé (Quellón)	Seawater
MT598068	M2	Ia	12-06-2018	*O. kisutch*	Chiloé (Quellón)	Seawater
MT598074	M2	Ia	19-06-2018	*O. kisutch*	Chiloé (Caucahue)	Seawater
MT598069	M2	Ia	21-06-2018	*O. kisutch*	Chiloé (Detico)	Seawater
MT598075	M2	Ib	27-03-2018	*O. kisutch*	Pargua (P. Montt) ^2^	Freshwater
MT598076	M2	Ib	05-12-2018	*S. salar*	Chiloé (Voigue)	Seawater
MT598077	M2	Ib	19-12-2019	*S. salar*	Lenca	rFeshwater
MT598078	M2	Ib	19-12-2019	*S. salar*	Lenca	Freshwater
MT598070	M2	Ib	04-12-2018	*S. salar*	Chiloé (Quicavi)	Seawater
MT598079	S1	Ib	06-04-2018	*O. kisutch*	Chiloé (Calen)	Seawater
MT598080	S1	Ia	12-04-2018	*S. salar*	Chiloé (Quellón)	Seawater
MT598081	S1	Ia	26-04-2018	*O. kisutch*	Chiloé (Quellón)	Seawater
MT598082	S1	Ia	12-06-2018	*O. kisutch*	Chiloé (Quellón)	Seawater
MT598083	S1	Ia	19-06-2018	*O. kisutch*	Chiloé (Caucahue)	Seawater
MT598084	S1	Ia	21-06-2018	*O. kisutch*	Chiloé (Detico)	Seawater
MT598085	S1	Ib	27-03-2018	*O. kisutch*	Pargua (P. Montt) ^2^	Freshwater
MT598086	S1	Ib	04-12-2018	*S. salar*	Chiloé (Quicavi)	Seawater
MT598087	S1	Ib	05-12-2018	*S. salar*	Chiloé (Voigue)	Seawater
MT598088	S1	Ib	17-06-2019	*S. salar*	Pargua (P. Montt) ^2^	Seawater
MT598089	S1	Ia	07-08-2019	*O. kisutch*	Puerto Cisne	Seawater

^1^ O. kisutch (Oncorhynchus kisutch); S. salar (Salmo salar); ^2^ P. Montt, Puerto Montt.

## Data Availability

The data presented in this study are available in the Supplementary Materials.

## Supplementary Materials

The following are available online at https://www.mdpi.com/2076-0817/10/1/41/s1, Figure S1: Rectangular version of phylogenetic distances from the alignment of S1 sequences above 400 nucleotides; Figure S2: Phylogenetic trees of segments L1, L2, L3, M1, M3, S2, S3 and S4; Figure S3: Phylogenetic distances from the alignment of 40 μ1 amino acid sequences grouped by genotype and represented as heatmaps; Figure S4: Phylogenetic distances from the alignment of 61 σ3 amino acid sequences grouped by genotype and represented as heatmaps; Figure S5: Phylogenetic distances from the alignment of μ1 sequences in Figure S4, grouped by country and represented as heatmaps; Figure S6: Phylogenetic distances from the alignment of σ3 sequences in Figure S4, grouped by host and represented as heatmaps; Table S1: GenBank Accession Numbers and metadata for PRV sequences of all ten genomic segments listed under the Tax ID: txid1157337 in the NCBI GenBank database to date (May 2020). Accession numbers and metadata of all sequences retrieved from NCBI GenBank used in this study; Table S2: GenBank accession numbers and metadata of PRV segments S1 and M2 sequences listed under the Tax ID: txid1157337 in the NCBI GenBank database to date (May 2020) used in this study.
