# Supplementary material for: Extensive Phylogenetic Analysis of Piscine Orthoreovirus Genomic Sequences Shows the Robustness of Subgenotype Classification
Source: Pathogens. 2021 Jan 7;10(1):41. doi: 10.3390/pathogens10010041 (PMC7825714; doi:10.3390/pathogens10010041)
Supplement: Supplementary file 1 [file pathogens-10-00041-s001.zip › Supplementary folder/pathogens_Supplementary Table S1_December 13 2020.pdf]

Supplementary Table S1. GenBank Accession Numbers and metadata for PRV sequences of all 10 genomic segments listed under the Tax ID: txid1157337 in the NCBI GenBank database to date (May 2020) used in this study

GenBank Accession Number	PRV isolate	PRV subgenotype (Kibenge *et al.,* 2013; Godoy *et al.,* 2016)	PRV subtype (Dhamotaran *et al.,* 2018)	PRV Description	Country	Collection_date Year)	Host	Primary Author
AF059720	OUTGROUP	IIa	PRV3	outgroup description	Canada	1999	avian	Duncan
GU994016	Salmo/GP-2010/NOR	Ib	PRV1	Reovirus sp. Salmo/GP-2010/NOR segment M2, partial sequence	Norway	2010	Salmo salar	Palacios
GU994022	Salmo/GP-2010/NOR	Ib	PRV1	Reovirus sp. Salmo/GP-2010/NOR segment S1. partial sequence	Norway	2010	Salmo salar	Palacios
HG329842	35 Bjoreio	Ia	PRV1	Piscine reovirus partial mRNA for Sigma 3. segment S1. isolate 35 Bjoreio 2009 Wild	Norway	2009	Salmo salar	Garseth
HG329843	45 Eira	Ia	PRV1	Piscine reovirus partial mRNA for Sigma 3. segment S1. isolate 45 Eira 2009 Hatchery-reared	Norway	2009	Salmo salar	Garseth
HG329844	61 Eira	Ib	PRV1	Piscine reovirus partial mRNA for Sigma 3. segment S1. isolate 61 Eira 2009 Hatchery reared	Norway	2009	Salmo salar	Garseth
HG329845	81 Etne	Ib	PRV1	Piscine reovirus partial mRNA for Sigma 3. segment S1. isolate 81 Etne 2009 Wild	Norway	2009	Salmo salar	Garseth
HG329846	90 Etne	Ib	PRV1	Piscine reovirus partial mRNA for Sigma 3. segment S1. isolate 90 Etne 2009 Wild	Norway	2009	Salmo salar	Garseth
HG329847	93 Etne	Ib	PRV1	Piscine reovirus partial mRNA for Sigma 3. segment S1. isolate 93 Etne 2009 Wild	Norway	2009	Salmo salar	Garseth
HG329848	131 Gaula	Ia	PRV1	Piscine reovirus partial mRNA for Sigma 3. segment S1. isolate 131 Gaula 2009 Wild	Norway	2009	Salmo salar	Garseth
HG329849	182 Hestdal	Ia	PRV1	Piscine reovirus partial mRNA for Sigma 3. segment S1. isolate 182 Hestdal 2009 Wild	Norway	2009	Salmo salar	Garseth
HG329850	187 Hestdal	Ia	PRV1	Piscine reovirus partial mRNA for Sigma 3. segment S1. isolate 187 Hestdal 2009 Wild	Norway	2009	Salmo salar	Garseth
HG329851	190 Hestdal	Ia	PRV1	Piscine reovirus partial mRNA for Sigma 3. segment S1. isolate 190 Hestdal 2009 Wild	Norway	2009	Salmo salar	Garseth
HG329852	211 Mandal	Ia	PRV1	Piscine reovirus partial mRNA for Sigma 3. segment S1. isolate 211 Mandal 2009 Wild	Norway	2009	Salmo salar	Garseth
HG329853	220 Mandal	Ib	PRV1	Piscine reovirus partial mRNA for Sigma 3. segment S1. isolate 220 Mandal 2009 Wild	Norway	2009	Salmo salar	Garseth
HG329854	246 Mandal	Ia	PRV1	Piscine reovirus partial mRNA for Sigma 3. segment S1. isolate 246 Mandal 2009 Wild	Norway	2009	Salmo salar	Garseth
HG329855	261 Nidelv	Ib	PRV1	Piscine reovirus partial mRNA for Sigma 3. segment S1. isolate 261 Nidelv 2009 Escaped farmed	Norway	2009	Salmo salar	Garseth
HG329856	273 Skibotn	Ib	PRV1	Piscine reovirus partial mRNA for Sigma 3. segment S1. isolate 273 Skibotn 2009 Wild	Norway	2009	Salmo salar	Garseth
HG329857	283 Skjomen	Ib	PRV1	Piscine reovirus partial mRNA for Sigma 3. segment S1. isolate 283 Skjomen 2009 Escaped farmed	Norway	2009	Salmo salar	Garseth
HG329858	284 Stjordal	Ia	PRV1	Piscine reovirus partial mRNA for Sigma 3. segment S1. isolate 284 Stjordal 2009 Wild	Norway	2009	Salmo salar	Garseth
HG329859	307 Stjordal	Ia	PRV1	Piscine reovirus partial mRNA for Sigma 3. segment S1. isolate 307 Stjordal 2009 Wild	Norway	2009	Salmo salar	Garseth
HG329860	318 Storelva Holt	Ib	PRV1	Piscine reovirus partial mRNA for Sigma 3. segment S1. isolate 318 Storelva Holt 2009 Wild	Norway	2009	Salmo salar	Garseth
HG329861	338 Surna	Ib	PRV1	Piscine reovirus partial mRNA for Sigma 3. segment S1. isolate 338 Surna 2009 Escaped farmed	Norway	2009	Salmo salar	Garseth
HG329862	350 Surna	Ib	PRV1	Piscine reovirus partial mRNA for Sigma 3. segment S1. isolate 350 Surna 2009 Escaped farmed	Norway	2009	Salmo salar	Garseth
HG329863	407 Vosso	Ia	PRV1	Piscine reovirus partial mRNA for Sigma 3. segment S1. isolate 407 Vosso 2009 Wild	Norway	2009	Salmo salar	Garseth
HG329864	411 Vosso	Ib	PRV1	Piscine reovirus partial mRNA for Sigma 3. segment S1. isolate 41 Vosso 2009 Wild	Norway	2009	Salmo salar	Garseth
HG329865	412 Vosso	Ib	PRV1	Piscine reovirus partial mRNA for Sigma 3. segment S1. isolate 412 Vosso 2009 Hatchery-reared	Norway	2009	Salmo salar	Garseth
HG329866	414 Vosso	Ib	PRV1	Piscine reovirus partial mRNA for Sigma 3. segment S1. isolate 414 Vosso 2009 Hatchery-reared	Norway	2009	Salmo salar	Garseth
HG329867	438 Alta	Ib	PRV1	Piscine reovirus partial mRNA for Sigma 3. segment S1. isolate 438 Alta 2008 Wild	Norway	2008	Salmo salar	Garseth
HG329868	445 Alta	Ia	PRV1	Piscine reovirus partial mRNA for Sigma 3. segment S1. isolate 445 Alta 2008 Wild	Norway	2008	Salmo salar	Garseth
HG329869	470 Drevja	Ia	PRV1	Piscine reovirus partial mRNA for Sigma 3. segment S1. isolate 470 Drevja 2008 Wild	Norway	2008	Salmo salar	Garseth
HG329870	491 Eira	Ib	PRV1	Piscine reovirus partial mRNA for Sigma 3. segment S1. isolate 491 Eira 2008 Wild	Norway	2008	Salmo salar	Garseth
HG329871	517 Eira	Ia	PRV1	Piscine reovirus partial mRNA for Sigma 3. segment S1. isolate 517 Eira 2008 Wild	Norway	2008	Salmo salar	Garseth
HG329872	522 Ekso	Ib	PRV1	Piscine reovirus partial mRNA for Sigma 3. segment S1. isolate 522 Ekso 2008 Wild	Norway	2008	Salmo salar	Garseth
HG329873	555 Fusta	Ib	PRV1	Piscine reovirus partial mRNA for Sigma 3. segment S1. isolate 555 Fusta 2008 Wild	Norway	2008	Salmo salar	Garseth
HG329874	565 Gaula	Ib	PRV1	Piscine reovirus partial mRNA for Sigma 3. segment S1. isolate 565 Gaula 2008 Wild	Norway	2008	Salmo salar	Garseth
HG329875	629 Jolstra	Ia	PRV1	Piscine reovirus partial mRNA for Sigma 3. segment S1. isolate 629 Jolstra 2008 Wild	Norway	2008	Salmo salar	Garseth
HG329876	708 Nausta	Ia	PRV1	Piscine reovirus partial mRNA for Sigma 3. segment S1. isolate 708 Nausta 2008 Wild	Norway	2008	Salmo salar	Garseth
HG329877	818 Surna	Ib	PRV1	Piscine reovirus partial mRNA for Sigma 3. segment S1. isolate 818 Surna 2008 Wild	Norway	2008	Salmo salar	Garseth
HG329878	842 Vikja	Ia	PRV1	Piscine reovirus partial mRNA for Sigma 3. segment S1. isolate 842 Vikja 2008 Wild	Norway	2008	Salmo salar	Garseth
HG329879	851 Vikja	Ia	PRV1	Piscine reovirus partial mRNA for Sigma 3. segment S1. isolate 851 Vikja 2008 Wild	Norway	2008	Salmo salar	Garseth
HG329880	866 Vikja	Ia	PRV1	Piscine reovirus partial mRNA for Sigma 3. segment S1. isolate 866 Vikja 2008 Wild	Norway	2008	Salmo salar	Garseth
HG329881	907 Vosso	Ia	PRV1	Piscine reovirus partial mRNA for Sigma 3. segment S1. isolate 907 Vosso 2008 Hatchery-reared	Norway	2008	Salmo salar	Garseth
HG329882	909 Vosso	Ia	PRV1	Piscine reovirus partial mRNA for Sigma 3. segment S1. isolate 909 Vosso 2008 Hatchery-reared	Norway	2008	Salmo salar	Garseth
HG329883	931 Alta	Ia	PRV1	Piscine reovirus partial mRNA for Sigma 3. segment S1. isolate 931 Alta 2007 Wild	Norway	2007	Salmo salar	Garseth
HG329884	982 Eira	Ib	PRV1	Piscine reovirus partial mRNA for Sigma 3. segment S1. isolate 982 Eira 2007 Wild	Norway	2007	Salmo salar	Garseth
HG329885	985 Ekso	Ia	PRV1	Piscine reovirus partial mRNA for Sigma 3. segment S1. isolate 985 Ekso 2007 wild	Norway	2007	Salmo salar	Garseth
HG329886	987 Ekso	Ia	PRV1	Piscine reovirus partial mRNA for Sigma 3. segment S1. isolate 987 Ekso 2007 wild	Norway	2007	Salmo salar	Garseth
HG329887	989 Ekso	Ia	PRV1	Piscine reovirus partial mRNA for Sigma 3. segment S1. isolate 989 Ekso 2007 Wild	Norway	2007	Salmo salar	Garseth
HG329888	993 Ekso	Ia	PRV1	Piscine reovirus partial mRNA for Sigma 3. segment S1. isolate 993 Ekso 2007 Wild	Norway	2007	Salmo salar	Garseth
HG329889	1039 Laerdal	Ia	PRV1	Piscine reovirus partial mRNA for Sigma 3. segment S1. isolate 1039 Laerdal 2007 Wild	Norway	2007	Salmo salar	Garseth
HG329890	1062 Mandal	Ia	PRV1	Piscine reovirus partial mRNA for Sigma 3. segment S1. isolate 1062 Mandal 2007 wild	Norway	2007	Salmo salar	Garseth
HG329891	1137 Stjordal	Ia	PRV1	Piscine reovirus partial mRNA for Sigma 3. segment S1. isolate 1137 Stjordal 2007 Wild	Norway	2007	Salmo salar	Garseth
HG329892	1160 Surna	Ib	PRV1	Piscine reovirus partial mRNA for Sigma 3. segment S1. isolate 1160 Surna 2007 Wild	Norway	2007	Salmo salar	Garseth
HG329893	1195 Aaroy	Ia	PRV1	Piscine reovirus partial mRNA for Sigma 3. segment S1. isolate 1195 Aaroy 2007 Wild	Norway	2007	Salmo salar	Garseth
HG329894	1261 Halsan	Ib	PRV1	Piscine reovirus partial mRNA for Sigma 3. segment S1. isolate 1261 Halsan 2009 Escaped farmed	Norway	2009	Salmo salar	Garseth
HG329895	1309 Eidsdal	Ib	PRV1	Piscine reovirus partial mRNA for Sigma 3. segment S1. isolate 1309 Eidsdal 2008 Wild	Norway	2008	Salmo salar	Garseth
HG329896	1343 Moelv	Ia	PRV1	Piscine reovirus partial mRNA for Sigma 3. segment S1. isolate 1343 Moelv 2008 Wild	Norway	2008	Salmo salar	Garseth
HG329897	1361 Moelv	Ib	PRV1	Piscine reovirus partial mRNA for Sigma 3. segment S1. isolate 1361 Moelv 2008 Sea-trout	Norway	2008	Salmo salar	Garseth
HG329898	1459 Etne	Ib	PRV1	Piscine reovirus partial mRNA for Sigma 3. segment S1. isolate 1459 Etne 2010 Escaped farmed	Norway	2010	Salmo salar	Garseth
HG329899	1462 Etne	Ib	PRV1	Piscine reovirus partial mRNA for Sigma 3. segment S1. isolate 1462 Etne 2010 Escaped farmed	Norway	2010	Salmo salar	Garseth
HG329900	1463 Etne	Ib	PRV1	Piscine reovirus partial mRNA for Sigma 3. segment S1. isolate 1463 Etne 2010 Escaped farmed	Norway	2010	Salmo salar	Garseth
HG329901	1469 Etne	Ib	PRV1	Piscine reovirus partial mRNA for Sigma 3. segment S1. isolate 1469 Etne 2010 Escaped farmed	Norway	2010	Salmo salar	Garseth
JN991006	5433-S3	Ia	PRV1	Piscine reovirus isolate 5433 sigma 3 protein gene. partial cds	Norway	2010	Salmo salar	Lovoll
JN991007	1921-S3	Ia	PRV1	Piscine reovirus isolate 1921 sigma 3 protein gene. partial cds	Norway	2010	Salmo salar	Lovoll
JN991008	9326-S3	Ia	PRV1	Piscine reovirus isolate 9326 sigma 3 protein gene. partial cds	Norway	2010	Salmo salar	Lovoll
JN991009	7243-S3	Ib	PRV1	Piscine reovirus isolate 7243 sigma 3 protein gene. partial cds	Norway	2010	Salmo salar	Lovoll
JN991010	7030-S3	Ib	PRV1	Piscine reovirus isolate 7030 sigma 3 protein gene. partial cds	Norway	2010	Salmo salar	Lovoll
JN991011	8286-S3	Ib	PRV1	Piscine reovirus isolate 8286 sigma 3 protein gene. partial cds	Norway	2010	Salmo salar	Lovoll
JN991012	3817-S3	Ia	PRV1	Piscine reovirus isolate 3817 sigma 3 protein gene. partial cds	Norway	2010	Salmo salar	Lovoll
JX046542	PRV-M2-R11/11	unknown	unknown	Piscine reovirus isolate PRV-M2-R11/11 outer capsid protein [M2] gene, partial cds	Norway	2011	Salmo salar	Tolaas
JX046543	PRV-M2-R12/11	unknown	unknown	Piscine reovirus isolate PRV-M2-R12/11 outer capsid protein [M2] gene, partial cds	Norway	2011	Salmo salar	Tolaas
KC473452	VT02292012-163	Ia	PRV1	Piscine reovirus isolate VT02292012-163 sigma 1 and sigma 1S genes. complete cds	Canada	2012	Salmo salar	Kibenge
KC473453	VT06062012-358	Ia	PRV1	Piscine reovirus isolate VT06062012-358 sigma 1 and sigma 1S genes. complete cds	Canada	2012	Salmo salar	Kibenge
KC473454	VT06202012-371	Ia	PRV1	Piscine reovirus isolate VT06202012-371 sigma 1 and sigma 1S genes. complete cds	Canada	2012	Salmo salar	Kibenge
KC715683	VT06062012-358	Ia	PRV1	Piscine reovirus isolate VT06062012-358 outer shell protein mRNA, complete cds	Canada	2012	Salmo salar	Kibenge
KC776260	VT06202012-371	Ia	PRV1	Piscine reovirus isolate VT06202012-371 outer shell protein mRNA, complete cds	Canada	2012	Salmo salar	Kibenge
KC782501	CGA337	Ib	PRV1	UNVERIFIED: Piscine reovirus isolate CGA/337/2012 sigma 3-like gene. partial sequence	Chile	2012	Salmo salar	Kibenge
KC790988	CGA8857	Ib	PRV1	UNVERIFIED: Piscine reovirus isolate CGA/8857/2012 sigma 3-like gene. partial sequence	Chile	2012	Salmo salar	Kibenge
KC795569	CGA280-05	Ib	PRV1	Piscine reovirus isolate CGA280-05 outer shell protein mRNA, complete cds	Chile	2012	Salmo salar	Kibenge
KC795571	CGA280-05	Ib	PRV1	Piscine reovirus isolate CGA280-05 outer clamp protein and non-structural protein genes. complete cds	Chile	2012	Salmo salar	Kibenge
KC795593	VT02292012-163	Ia	PRV1	Piscine reovirus isolate VT02292012-163 outer shell protein mRNA, partial cds	Canada	2012	Salmo salar	Kibenge
KC795594	VT02292012-167	Ia	PRV1	Piscine reovirus isolate VT02292012-167 outer shell protein mRNA, partial cds	Canada	2012	Salmo salar	Kibenge
KC795599	VT02292012-167	Ia	PRV1	Piscine reovirus isolate VT02292012-167 outer clamp protein and non-structural protein genes. complete	Canada	2012	Salmo salar	Kibenge
KC795600	VT03202012-196	Ia	PRV1	Piscine reovirus isolate VT03202012-196 outer clamp protein and non-structural protein genes. complete	Canada	2012	Salmo salar	Kibenge
KC795601	VT03202012-209	Ia	PRV1	Piscine reovirus isolate VT03202012-209 outer clamp protein and non-structural protein genes. complete	Canada	2012	Salmo salar	Kibenge
KR337477	50607	Ib	PRV1	Piscine reovirus isolate 050607 segment M2 mu 1 protein gene, complete cds	Norway	2007	Salmo salar	Haatveit
KR337479	50607	Ib	PRV1	Piscine reovirus isolate 050607 segment S1 sigma 3 protein and p13 protein genes. complete cds	Norway	2007	Salmo salar	Haatveit
KR347078	BCA1849_01	Ia	PRV1	Piscine reovirus isolate BCA1849_01 sigma 3 gene. partial cds; and protein p13 gene. complete cds	Canada	2001	Salmo salar	Siah
KR347079	BCA1850_01	Ia	PRV1	Piscine reovirus isolate BCA1850_01 sigma 3 gene. partial cds; and protein p13 gene. complete cds	Canada	2001	Salmo salar	Siah
KR347080	BCA1854_05	Ia	PRV1	Piscine reovirus isolate BCA1854_05 sigma 3 gene. partial cds; and protein p13 gene. complete cds	Canada	2005	Salmo salar	Siah
KR347081	BCJ18824_13	Ia	PRV1	Piscine reovirus isolate BCJ18824_13 sigma 3 gene. partial cds; and protein p13 gene. complete cds	Canada	2013	Oncorhynchus tshawytscha	Siah
KR347082	BCJ19323_13	Ia	PRV1	Piscine reovirus isolate BCJ19323_13 sigma 3 gene. partial cds; and protein p13 gene. complete cds	Canada	2013	Oncorhynchus kisutch	Siah
KR347083	BCJ19943_13	Ia	PRV1	Piscine reovirus isolate BCJ19943_13 sigma 3 gene. partial cds; and protein p13 gene. complete cds	Canada	2013	Oncorhynchus kisutch	Siah
KR347084	BCJ24201_13	Ia	PRV1	Piscine reovirus isolate BCJ24201_13 sigma 3 gene. partial cds; and protein p13 gene. complete cds	Canada	2013	Salmo salar	Siah
KR347085	BCJ28529_13	Ia	PRV1	Piscine reovirus isolate BCJ28529_13 sigma 3 gene. partial cds; and protein p13 gene. complete cds	Canada	2013	Salmo salar	Siah
KR347086	BCJ28537_13	Ia	PRV1	Piscine reovirus isolate BCJ28537_13 sigma 3 gene. partial cds; and protein p13 gene. complete cds	Canada	2013	Salmo salar	Siah
KR347087	BCJ28545_13	Ia	PRV1	Piscine reovirus isolate BCJ28545_13 sigma 3 gene. partial cds; and protein p13 gene. complete cds	Canada	2013	Salmo salar	Siah
KR347088	BCJ31910_13	Ia	PRV1	Piscine reovirus isolate BCJ31910_13 sigma 3 gene. partial cds; and protein p13 gene. complete cds	Canada	2013	Salmo salar	Siah
KR347089	BCJ31914_13	Ia	PRV1	Piscine reovirus isolate BCJ31914_13 sigma 3 gene. partial cds; and protein p13 gene. complete cds	Canada	2013	Salmo salar	Siah
KR347090	BCJ31915_13	Ia	PRV1	Piscine reovirus isolate BCJ31915_13 sigma 3 gene. partial cds; and protein p13 gene. complete cds	Canada	2013	Salmo salar	Siah
KR347091	BCJ31916_13	Ia	PRV1	Piscine reovirus isolate BCJ31916_13 sigma 3 gene. partial cds; and protein p13 gene. complete cds	Canada	2013	Salmo salar	Siah
KR347092	BCJ31920_13	Ia	PRV1	Piscine reovirus isolate BCJ31920_13 sigma 3 gene. partial cds; and protein p13 gene. complete cds	Canada	2013	Salmo salar	Siah
KR347093	BCJ34056_13	Ia	PRV1	Piscine reovirus isolate BCJ34056_13 sigma 3 gene. partial cds; and protein p13 gene. complete cds	Canada	2013	Oncorhynchus kisutch	Siah
KR347094	BCJ35240_13	Ia	PRV1	Piscine reovirus isolate BCJ35240_13 sigma 3 gene. partial cds; and protein p13 gene. complete cds	Canada	2013	Salmo salar	Siah
KR347095	BCJ35246_13	Ia	PRV1	Piscine reovirus isolate BCJ35246_13 sigma 3 gene. partial cds; and protein p13 gene. complete cds	Canada	2013	Salmo salar	Siah
KR347096	BCJ35249_13	Ia	PRV1	Piscine reovirus isolate BCJ35249_13 sigma 3 gene. partial cds; and protein p13 gene. complete cds	Canada	2013	Salmo salar	Siah
KR347097	BCJ35255_13	Ia	PRV1	Piscine reovirus isolate BCJ35255_13 sigma 3 gene. partial cds; and protein p13 gene. complete cds	Canada	2013	Salmo salar	Siah
KR347098	BCJ35256_13	Ia	PRV1	Piscine reovirus isolate BCJ35256_13 sigma 3 gene. partial cds; and protein p13 gene. complete cds	Canada	2013	Salmo salar	Siah
KR347099	BCJ37896_13	Ia	PRV1	Piscine reovirus isolate BCJ37896_13 sigma 3 gene. partial cds; and protein p13 gene. complete cds	Canada	2013	Oncorhynchus kisutch	Siah
KR347100	BCJ40723_13	Ia	PRV1	Piscine reovirus isolate BCJ40723_13 sigma 3 gene. partial cds; and protein p13 gene. complete cds	Canada	2013	Salmo salar	Siah
KR347101	BCJ40731_13	Ia	PRV1	Piscine reovirus isolate BCJ40731_13 sigma 3 gene. partial cds; and protein p13 gene. complete cds	Canada	2013	Salmo salar	Siah
KR347102	BCJ40740_13	Ia	PRV1	Piscine reovirus isolate BCJ40740_13 sigma 3 gene. partial cds; and protein p13 gene. complete cds	Canada	2013	Salmo salar	Siah
KR347103	BCJ378151_13	Ia	PRV1	Piscine reovirus isolate BCJ378151_13 sigma 3 gene. partial cds; and protein p13 gene. complete cds	Canada	2013	Oncorhynchus kisutch	Siah
KR347104	BCJ378241_13	Ia	PRV1	Piscine reovirus isolate BCJ378241_13 sigma 3 gene. partial cds; and protein p13 gene. complete cds	Canada	2013	Oncorhynchus kisutch	Siah
KR347105	BCJ402256_13	Ia	PRV1	Piscine reovirus isolate BCJ402256_13 sigma 3 gene. partial cds; and protein p13 gene. complete cds	Canada	2013	Oncorhynchus kisutch	Siah
KR347106	BCJ402276_13	Ia	PRV1	Piscine reovirus isolate BCJ402276_13 sigma 3 gene. partial cds; and protein p13 gene. complete cds	Canada	2013	Oncorhynchus kisutch	Siah
KR347107	BCJ402334_13	Ia	PRV1	Piscine reovirus isolate BCJ402334_13 sigma 3 gene. partial cds; and protein p13 gene. complete cds	Canada	2013	Oncorhynchus kisutch	Siah
KR347108	BCK1435_14	Ia	PRV1	Piscine reovirus isolate BCK1435_14 sigma 3 gene. partial cds; and protein p13 gene. complete cds	Canada	2014	Salmo salar	Siah
KR347109	BCK1436_14	Ia	PRV1	Piscine reovirus isolate BCK1436_14 sigma 3 gene. partial cds; and protein p13 gene. complete cds	Canada	2014	Salmo salar	Siah
KR347110	BCK1562_14	Ia	PRV1	Piscine reovirus isolate BCK1562_14 sigma 3 gene. partial cds; and protein p13 gene. complete cds	Canada	2014	Oncorhynchus kisutch	Siah
KR347111	BCK1566_14	Ia	PRV1	Piscine reovirus isolate BCK1566_14 sigma 3 gene. partial cds; and protein p13 gene. complete cds	Canada	2014	Oncorhynchus kisutch	Siah
KR347112	BCK14114_14	Ia	PRV1	Piscine reovirus isolate BCK14114_14 sigma 3 gene. partial cds; and protein p13 gene. complete cds	Canada	2014	Salmo salar	Siah
KR347113	BCK14120_14	Ia	PRV1	Piscine reovirus isolate BCK14120_14 sigma 3 gene. partial cds; and protein p13 gene. complete cds	Canada	2014	Salmo salar	Siah
KR347114	BCK14310_14	Ia	PRV1	Piscine reovirus isolate BCK14310_14 sigma 3 gene. partial cds; and protein p13 gene. complete cds	Canada	2014	Salmo salar	Siah
KR347115	BCK15625_14	Ia	PRV1	Piscine reovirus isolate BCK15625_14 sigma 3 gene. partial cds; and protein p13 gene. complete cds	Canada	2014	Oncorhynchus kisutch	Siah
KR478633	WS1207_12	Ia	PRV1	Piscine reovirus isolate WS1207_12 sigma 3 gene. partial cds; and p13 protein gene. complete cds	USA	2012	Oncorhynchus tshawytscha	Siah
KR478634	WS1209_12	Ia	PRV1	Piscine reovirus isolate WS1209_12 sigma 3 gene. partial cds; and p13 protein gene. complete cds	USA	2012	Oncorhynchus tshawytscha	Siah
KR478635	WSKFH1_14	Ia	PRV1	Piscine reovirus isolate WSKFH1_14 sigma 3 gene. partial cds; and p13 protein gene. complete cds	USA	2014	Oncorhynchus kisutch	Siah
KR478636	WSKFH2_14	Ia	PRV1	Piscine reovirus isolate WSKFH2_14 sigma 3 gene. partial cds; and p13 protein gene. complete cds	USA	2014	Oncorhynchus kisutch	Siah
KR478637	WSKFH11_14	Ia	PRV1	Piscine reovirus isolate WSKFH11_14 sigma 3 gene. partial cds; and p13 protein gene. complete cds	USA	2014	Oncorhynchus kisutch	Siah
KR478638	WSKFH12_14	Ia	PRV1	Piscine reovirus isolate WSKFH12_14 sigma 3 gene. partial cds; and p13 protein gene. complete cds	USA	2014	Oncorhynchus kisutch	Siah
KR478639	WSKFH13_14	Ia	PRV1	Piscine reovirus isolate WSKFH13_14 sigma 3 gene. partial cds; and p13 protein gene. complete cds	USA	2014	Oncorhynchus kisutch	Siah
KR478640	AKJ20115_13	Ia	PRV1	Piscine reovirus isolate AKJ20115_13 sigma 3 gene. partial cds; and sigma 3 gene. complete cds	USA	2013	Oncorhynchus kisutch	Siah
KR478641	AKJ20120_13	Ia	PRV1	Piscine reovirus isolate AKJ20120_13 sigma 3 gene. partial cds; and p13 protein gene. complete cds	USA	2013	Oncorhynchus kisutch	Siah
KR478642	BCA1338_01	Ia	PRV1	Piscine reovirus isolate BCA1338_01 sigma 3 gene. partial cds; and p13 protein gene. complete cds	Canada	2001	Oncorhynchus tshawytscha	Siah
KR478643	BCA1846_01	Ia	PRV1	Piscine reovirus isolate BCA1846_01 sigma 3 gene. partial cds; and p13 protein gene. complete cds	Canada	2001	Salmo salar	Siah
KR478644	BCA1848_01	Ia	PRV1	Piscine reovirus isolate BCA1848_01 sigma 3 gene. partial cds; and p13 protein gene. complete cds	Canada	2001	Salmo salar	Siah
KR558677	BC131_13	Ia	PRV1	Piscine reovirus isolate BC131_13 sigma 3 gene. partial cds	Canada	2013	Salmo salar	Siah
KR558678	BC132_13	Ia	PRV1	Piscine reovirus isolate BC132_13 sigma 3 gene. partial cds	Canada	2013	Salmo salar	Siah
KR558679	BC133_13	Ia	PRV1	Piscine reovirus isolate BC133_13 sigma 3 gene. partial cds	Canada	2013	Salmo salar	Siah
KR558680	BC134_13	Ia	PRV1	Piscine reovirus isolate BC134_13 sigma 3 gene. partial cds	Canada	2013	Salmo salar	Siah
KR558681	BC135_13	Ia	PRV1	Piscine reovirus isolate BC135_13 sigma 3 gene. partial cds	Canada	2013	Salmo salar	Siah
KR558682	BC136_13	Ia	PRV1	Piscine reovirus isolate BC136_13 sigma 3 gene. partial cds	Canada	2013	Salmo salar	Siah
KR558683	BC137_13	Ia	PRV1	Piscine reovirus isolate BC137_13 sigma 3 gene. partial cds	Canada	2013	Salmo salar	Siah
KR558684	BC138_13	Ia	PRV1	Piscine reovirus isolate BC138_13 sigma 3 gene. partial cds	Canada	2013	Salmo salar	Siah
KR558685	BC139_13	Ia	PRV1	Piscine reovirus isolate BC139_13 sigma 3 gene. partial cds	Canada	2013	Salmo salar	Siah
KR781117	BC1310_13	Ia	PRV1	Piscine reovirus isolate BC1310_13 sigma 3 gene. partial cds	Canada	2013	Salmo salar	Siah
KR781118	BC1311_13	Ia	PRV1	Piscine reovirus isolate BC1311_13 sigma 3 gene. partial cds	Canada	2013	Salmo salar	Siah
KR872635	BCinoc3	Ia	PRV1	Piscine reovirus isolate BCinoc3 segment S1 sigma 3 gene. partial cds	Canada	2012	Oncorhynchus tshawytscha	Garver
KR872636	BCinoc12	Ia	PRV1	Piscine reovirus isolate BCinoc12 segment S1 sigma 3 gene. partial cds	Canada	2012	Oncorhynchus tshawytscha	Garver
KR872637	BC361_14	Ia	PRV1	Piscine reovirus isolate BC361_14 segment S1 sigma 3 gene. partial cds	Canada	2014	Salmo salar	Garver
KT429734	BCJ19943_13	Ia	PRV1	Piscine reovirus isolate BCJ19943_13 outer shell protein mRNA, complete cds	Canada	2013	Oncorhynchus kisutch	Siah
KT429736	BCJ19943_13	Ia	PRV1	Piscine reovirus isolate BCJ19943_13 sigma 1 and sigma 1S mRNAs. complete cds	Canada	2013	Oncorhynchus kisutch	Siah
KT429744	BCJ31915_13	Ia	PRV1	Piscine reovirus isolate BCJ31915_13 outer shell protein mRNA, partial cds	Canada	2013	Salmo salar	Siah
KT429746	BCJ31915_13	Ia	PRV1	Piscine reovirus isolate BCJ31915_13 sigma 1 and sigma 1S mRNAs. complete cds	Canada	2013	Salmo salar	Siah
KT429754	WSKFH12_14	Ia	PRV1	Piscine reovirus isolate WSKFH12_14 outer shell protein mRNA, complete cds	USA	2014	Oncorhynchus kisutch	Siah
KT429756	WSKFH12_14	Ia	PRV1	Piscine reovirus isolate WSKFH12_14 sigma 1 and sigma 1S mRNAs. complete cds	USA	2014	Oncorhynchus kisutch	Siah
KT456500	VT09122012-755	Ia	PRV1	Piscine reovirus isolate VT09122012-755 segment S1 outer clamp protein and p13 protein genes. complete	Canada	2011	Oncorhynchus tshawytscha	Kibenge
KT456501	VT09192013-402	Ia	PRV1	Piscine reovirus isolate VT09192013-402 segment S1 outer clamp protein and p13 protein genes. complete	Canada	2013	Oncorhynchus kisutch	Kibenge
KT456502	VT09192013-408	Ia	PRV1	Piscine reovirus isolate VT09192013-408 segment S1 outer clamp protein and p13 protein genes. complete	Canada	2013	Oncorhynchus kisutch	Kibenge
KT456503	VT01212014-03	Ia	PRV1	Piscine reovirus isolate VT01212014-03 segment S1 outer clamp protein and p13 protein genes. complete	Canada	2014	Salmo salar	Kibenge
KT456504	VT01212014-04	Ia	PRV1	Piscine reovirus isolate VT01212014-04 segment S1 outer clamp protein and p13 protein genes. complete	Canada	2014	Salmo salar	Kibenge
KT456505	VT01212014-09	Ia	PRV1	Piscine reovirus isolate VT01212014-09 segment S1 outer clamp protein and p13 protein genes. complete	Iceland	2014	Salmo salar	Kibenge
KU131591	VT12202013_CGA_2013_	Ia	PRV1	Piscine reovirus isolate VT12202013-CGA-2013-4 segment S1 outer clamp protein and p13 protein genes	Chile	2013	Oncorhynchus kisutch	Godoy
KU131592	VT12202013_CGA_2013_	Ia	PRV1	Piscine reovirus isolate VT12202013-CGA-2013-1 segment S1 outer clamp protein and p13 protein genes	Chile	2013	Oncorhynchus kisutch	Godoy
KU131593	VT12202013_CGA_2013_	Ia	PRV1	Piscine reovirus isolate VT12202013-CGA-2013-2 segment S1 outer clamp protein and p13 protein genes	Chile	2013	Oncorhynchus kisutch	Godoy
KU131594	2015_CGA_2015_B	Ia	PRV1	Piscine reovirus isolate 2015-CGA-2015-B segment S1 outer clamp protein gene. partial cds; and p13	Chile	2015	Oncorhynchus kisutch	Godoy
KU131595	VT12202013_CGA_2013_	IIa	PRV3	Piscine reovirus isolate VT12202013-CGA-2013-3 segment S1 outer clamp protein and p13 protein genes	Chile	2013	Oncorhynchus kisutch	Godoy
KU131596	VT12202013_CGA_2013_	IIa	PRV3	Piscine reovirus isolate VT12202013-CGA-2013-5 segment S1 outer clamp protein and p13 protein genes	Chile	2013	Oncorhynchus kisutch	Godoy
KU131597	2013_CGA_2013_A	Ib	PRV1	Piscine reovirus isolate 2013-CGA-2013-A segment S1 outer clamp protein and p13 protein genes	Chile	2013	Salmo salar	Godoy
KU131598	2013_CGA_2013_C	Ib	PRV1	Piscine reovirus isolate 2013-CGA-2013-C segment S1 outer clamp protein and p13 protein genes	Chile	2013	Oncorhynchus kisutch	Godoy
KU131599	VT02182014_CGA_2013_	Ib	PRV1	Piscine reovirus isolate VT02182014-CGA-2013-8 segment S1 outer clamp protein and p13 protein genes	Chile	2013	Salmo salar	Godoy
KU131600	VT02182014_CGA_2013_	Ib	PRV1	Piscine reovirus isolate VT02182014-CGA-2013-9 segment S1 outer clamp protein and p13 protein genes	Chile	2013	Salmo salar	Godoy
KU131601	VT02182014_CGA_2013_	Ib	PRV1	Piscine reovirus isolate VT02182014-CGA-2013-10 segment S1 outer clamp protein and p13 protein genes	Chile	2013	Salmo salar	Godoy
KU131602	VT02182014_CGA_2013_	Ib	PRV1	Piscine reovirus isolate VT02182014-CGA-2013-6 segment S1 outer clamp protein and p13 protein genes	Chile	2013	Salmo salar	Godoy
KU131603	VT02182014_CGA_2013_	Ib	PRV1	Piscine reovirus isolate VT02182014-CGA-2013-7 segment S1 outer clamp protein and p13 protein genes	Chile	2013	Salmo salar	Godoy
KU131604	2015_CGA_2015_A	Ib	PRV1	Piscine reovirus isolate 2015-CGA-2015-A segment S1 outer clamp protein and p13 protein genes	Chile	2015	Oncorhynchus kisutch	Godoy
KU131605	2015_CGA_2015_C	Ib	PRV1	Piscine reovirus isolate 2015-CGA-2015-C segment S1 outer clamp protein gene. partial cds; and p13	Chile	2015	Salmo salar	Godoy
KU160513	VT01292015-09	Ia	PRV1	Piscine reovirus isolate VT01292015-09 segment S1 outer clamp protein and p13 protein genes. complete	Canada	2015	Salmo salar	Kibenge
KU160514	VT07222015-106	Ia	PRV1	Piscine reovirus isolate VT07222015-106 segment S1 outer clamp protein and p13 protein genes. complete	Canada	2015	Salmo salar	Kibenge

KU160515	VT07222015-107	Ia	PRV1	Piscine reovirus isolate VT07222015-107 segment S1 outer clamp protein and p13 protein genes. complete	Canada	2015	Salmo salar	Kibenge
KX844951	C10/P4.1	IIa	PRV3	Piscine reovirus isolate C10/P4.1 sigma 3 protein (OCPSigma3) gene. complete cds	Chile	2014	Oncorhynchus mykiss	Cartagena
KX844952	IM/P5.2	Ib	PRV1	Piscine reovirus isolate IM/P5.2 sigma 3 protein (OCPSigma3) gene. complete cds	Chile	2015	Oncorhynchus mykiss	Cartagena
KX844953	V/P3.2	Ia	PRV1	Piscine reovirus isolate V/P3.2 sigma 3 protein (OCPSigma3) gene. complete cds	Chile	2014	Oncorhynchus mykiss	Cartagena
KX844954	V/P3.1	Ia	PRV1	Piscine reovirus isolate V/P3.1 sigma 3 protein (OCPSigma3) gene. complete cds	Chile	2014	Oncorhynchus mykiss	Cartagena
KX844955	V/P2.2	Ia	PRV1	Piscine reovirus isolate V/P2.2 sigma 3 protein (OCPSigma3) gene. complete cds	Chile	2014	Oncorhynchus mykiss	Cartagena
KX844956	V/P2.1	Ia	PRV1	Piscine reovirus isolate V/P2.1 sigma 3 protein (OCPSigma3) gene. complete cds	Chile	2014	Oncorhynchus mykiss	Cartagena
KX844957	V/P1.2	Ia	PRV1	Piscine reovirus isolate V/P1.2 sigma 3 protein (OCPSigma3) gene. complete cds	Chile	2014	Oncorhynchus mykiss	Cartagena
KX844958	V/P1.1	Ia	PRV1	Piscine reovirus isolate V/P1.1 sigma 3 protein (OCPSigma3) gene. complete cds	Chile	2014	Oncorhynchus mykiss	Cartagena
KX844959	C10/P4.2	IIa	PRV3	Piscine reovirus isolate C10/P4.2 sigma 3 protein (OCPSigma3) gene. complete cds	Chile	2014	Oncorhynchus mykiss	Cartagena
KX844960	C10/P3.2	IIa	PRV3	Piscine reovirus isolate C10/P3.2 sigma 3 protein (OCPSigma3) gene. complete cds	Chile	2014	Oncorhynchus mykiss	Cartagena
KX844961	C10/P3.1	IIa	PRV3	Piscine reovirus isolate C10/P3.1 sigma 3 protein (OCPSigma3) gene. complete cds	Chile	2014	Oncorhynchus mykiss	Cartagena
KX844962	C10/P2.2	IIa	PRV3	Piscine reovirus isolate C10/P2.2 sigma 3 protein (OCPSigma3) gene. complete cds	Chile	2014	Oncorhynchus mykiss	Cartagena
KX844963	C10/P2.1	IIa	PRV3	UNVERIFIED: Piscine reovirus isolate C10/P2.1 sigma 3 protein-like (OCPSigma3) gene. complete sequence	Chile	2014	Oncorhynchus mykiss	Cartagena
KX844964	C10/P1.2	IIa	PRV3	Piscine reovirus isolate C10/P1.2 sigma 3 protein (OCPSigma3) gene. complete cds	Chile	2014	Oncorhynchus mykiss	Cartagena
KX844965	C10/P1.1	IIa	PRV3	Piscine reovirus isolate C10/P1.1 sigma 3 protein (OCPSigma3) gene. complete cds	Chile	2014	Oncorhynchus mykiss	Cartagena
KX851970	B5690	Ia	PRV1	dump description	Canada	2013	Salmo salar	Di Cicco
KX851971	B7274	Ia	PRV1	dump description	Canada	2013	Salmo salar	Di Cicco
KX851974	B7274	Ia	PRV1	Piscine reovirus isolate B7274 segment M2, complete sequence	Canada	2013	Salmo salar	Di Cicco
KX851975	B5690	Ia	PRV1	Piscine reovirus isolate B5690 segment M2, complete sequence	Canada	2013	Salmo salar	Di Cicco
KY369097	12-07_4	Ia	PRV1	Piscine orthoreovirus isolate 12-07_4 sigma 3 and p13 protein genes. partial cds	USA	2012	Oncorhynchus tshawytscha	Purcell
KY369098	12-09_1	Ia	PRV1	Piscine orthoreovirus isolate 12-09_1 sigma 3 and p13 protein genes. partial cds	USA	2012	Oncorhynchus tshawytscha	Purcell
KY369099	13-18_12	Ia	PRV1	Piscine orthoreovirus isolate 13-18_12 sigma 3 and p13 protein genes. partial cds	USA	2012	Oncorhynchus kisutch	Purcell
KY369100	13-26_10	Ia	PRV1	Piscine orthoreovirus isolate 13-26_10 sigma 3 and p13 protein genes. partial cds	USA	2012	Oncorhynchus kisutch	Purcell
KY369101	13-33_1389	Ia	PRV1	Piscine orthoreovirus isolate 13-33_1389 sigma 3 and p13 protein genes. partial cds	USA	2012	Oncorhynchus kisutch	Purcell
KY369102	13_33_1395	Ia	PRV1	Piscine orthoreovirus isolate 13_33_1395 sigma 3 and p13 protein genes. partial cds	USA	2012	Oncorhynchus kisutch	Purcell
KY369103	13_37_1361	Ia	PRV1	Piscine orthoreovirus isolate 13_37_1361 sigma 3 and p13 protein genes. partial cds	USA	2012	Oncorhynchus mykiss	Purcell
KY369104	13-6_1587	Ia	PRV1	Piscine orthoreovirus isolate 13-6_1587 sigma 3 and p13 protein genes. partial cds	USA	2012	Oncorhynchus tshawytscha	Purcell
KY369105	13-6_1594	Ia	PRV1	Piscine orthoreovirus isolate 13-6_1594 sigma 3 and p13 protein genes. partial cds	USA	2012	Oncorhynchus tshawytscha	Purcell
KY369106	14-11_3	Ia	PRV1	Piscine orthoreovirus isolate 14-11_3 sigma 3 and p13 protein genes. partial cds	USA	2012	Oncorhynchus tshawytscha	Purcell
KY369107	14-18_13	Ia	PRV1	Piscine orthoreovirus isolate 14-18_13 sigma 3 and p13 protein genes. partial cds	USA	2012	Salmo salar	Purcell
KY369108	14-21_16	Ia	PRV1	Piscine orthoreovirus isolate 14-21_16 sigma 3 and p13 protein genes. partial cds	USA	2012	Oncorhynchus kisutch	Purcell
KY369109	14-22_12	Ia	PRV1	Piscine orthoreovirus isolate 14-22_12 sigma 3 and p13 protein genes. partial cds	USA	2012	Oncorhynchus kisutch	Purcell
KY369110	14-29_15	Ia	PRV1	Piscine orthoreovirus isolate 14-29_15 sigma 3 and p13 protein genes. partial cds	USA	2012	Oncorhynchus kisutch	Purcell
KY369111	14-29_2	Ia	PRV1	Piscine orthoreovirus isolate 14-29_2 sigma 3 and p13 protein genes. partial cds	USA	2012	Oncorhynchus kisutch	Purcell
KY369112	14-30_5	Ia	PRV1	Piscine orthoreovirus isolate 14-30_5 sigma 3 and p13 protein genes. partial cds	USA	2013	Oncorhynchus tshawytscha	Purcell
KY369113	15-10_8	Ia	PRV1	Piscine orthoreovirus isolate 15-10_8 sigma 3 and p13 protein genes. partial cds	USA	2013	Oncorhynchus kisutch	Purcell
KY369114	15-3_9	Ia	PRV1	Piscine orthoreovirus isolate 15-3_9 sigma 3 and p13 protein genes. partial cds	USA	2013	Oncorhynchus tshawytscha	Purcell
KY369115	15-8_2	Ia	PRV1	Piscine orthoreovirus isolate 15-8_2 sigma 3 and p13 protein genes. partial cds	USA	2013	Oncorhynchus kisutch	Purcell
KY369116	15-9_5	Ia	PRV1	Piscine orthoreovirus isolate 15-9_5 sigma 3 and p13 protein genes. partial cds	USA	2013	Oncorhynchus kisutch	Purcell
KY369117	15-28_9	Ia	PRV1	Piscine orthoreovirus isolate 15-28_9 sigma 3 and p13 protein genes. partial cds	USA	2013	Oncorhynchus gorbuscha	Purcell
KY369118	15-29_5	Ia	PRV1	Piscine orthoreovirus isolate 15-29_5 sigma 3 and p13 protein genes. partial cds	USA	2013	Oncorhynchus kisutch	Purcell
KY369119	15-29_8	Ia	PRV1	Piscine orthoreovirus isolate 15-29_8 sigma 3 and p13 protein genes. partial cds	USA	2013	Oncorhynchus kisutch	Purcell
KY369120	15-30_15	Ia	PRV1	Piscine orthoreovirus isolate 15-30_15 sigma 3 and p13 protein genes. partial cds	USA	2013	Oncorhynchus kisutch	Purcell
KY369121	15-56_17	Ia	PRV1	Piscine orthoreovirus isolate 15-56_17 sigma 3 and p13 protein genes. partial cds	USA	2013	Oncorhynchus kisutch	Purcell
KY369122	15-57_1	Ia	PRV1	Piscine orthoreovirus isolate 15-57_1 sigma 3 and p13 protein genes. partial cds	USA	2013	Oncorhynchus kisutch	Purcell
KY369123	15-57_8	Ia	PRV1	Piscine orthoreovirus isolate 15-57_8 sigma 3 and p13 protein genes. partial cds	USA	2013	Oncorhynchus kisutch	Purcell
KY369124	15-32_3	Ia	PRV1	Piscine orthoreovirus isolate 15-32_3 p13 protein and sigma 3 genes. partial cds	USA	2013	Oncorhynchus kisutch	Purcell
KY369125	15-21_14	Ia	PRV1	Piscine orthoreovirus isolate 15-21_14 sigma 3 and p13 protein genes. partial cds	USA	2013	Oncorhynchus tshawytscha	Purcell
KY369126	15-26_1	Ia	PRV1	Piscine orthoreovirus isolate 15-26_1 sigma 3 and p13 protein genes. partial cds	USA	2013	Oncorhynchus kisutch	Purcell
KY379959	15-34_11	Ia	PRV1	Piscine orthoreovirus isolate 15-34_11 sigma 3 and p13 protein genes. partial cds	USA	2013	Oncorhynchus kisutch	Purcell
KY429947	NOR2012-V3621	Ib	PRV1	Piscine orthoreovirus isolate NOR2012-V3621 segment M2, complete sequence	Norway	2012	Salmo salar	Wessel
KY429949	NOR2012-V3621	Ib	PRV1	dump description	Norway	2012	Salmo salar	Wessel
LC145613	PRV-2	IIb	PRV2	Piscine orthoreovirus 2 genomic RNA, segment M2, complete sequence	Japan	2012	Oncorhynchus kisutch	Takano
LC145616	PRV-2	IIb	PRV2	Piscine orthoreovirus 2 genomic RNA, segment S1, complete sequence	Japan	2012	Oncorhynchus kisutch	Takano
LN680851	F445-2013/NOR	IIa	PRV3	Reovirus sp. rainbow trout/F445-2013/NOR partial segment 1 for sigma 3 protein	Norway	2013	Oncorhynchus mykiss	Olsen
MF946290	VT10092015-122	Ia	PRV1	Piscine orthoreovirus isolate VT10092015-122 outer clamp protein and p13 genes. complete cds	Canada	2015	Salmo salar	Kibenge
MF946291	VT10092015-123	Ia	PRV1	Piscine orthoreovirus isolate VT10092015-123 outer clamp protein and p13 genes. complete cds	Canada	2015	Salmo salar	Kibenge
MF946292	VT10092015-125	Ia	PRV1	Piscine orthoreovirus isolate VT10092015-125 outer clamp protein and p13 genes. complete cds	Canada	2015	Salmo salar	Kibenge
MF946293	VT12072015-130	Ia	PRV1	Piscine orthoreovirus isolate VT12072015-130 outer clamp protein and p13 genes. complete cds	Canada	2015	Salmo salar	Kibenge
MF946294	VT12072015-132	Ia	PRV1	Piscine orthoreovirus isolate VT12072015-132 outer clamp protein and p13 genes. complete cds	Canada	2015	Salmo salar	Kibenge
MF946295	VT12072015-137	Ia	PRV1	Piscine orthoreovirus isolate VT12072015-137 outer clamp protein and p13 genes. complete cds	Canada	2015	Salmo salar	Kibenge
MF946296	VT12162015-141	Ia	PRV1	Piscine orthoreovirus isolate VT12162015-141 outer clamp protein and p13 genes. complete cds	Canada	2015	Salmo salar	Kibenge
MF946297	VT01222016-15	Ia	PRV1	Piscine orthoreovirus isolate VT01222016-15 outer clamp protein and p13 genes. complete cds	Canada	2015	Salmo salar	Kibenge
MF946298	VT06082016-37	Ia	PRV1	Piscine orthoreovirus isolate VT06082016-37 outer clamp protein and p13 genes. complete cds	Canada	2016	Salmo salar	Kibenge
MF946299	VT02162017-59	Ia	PRV1	Piscine orthoreovirus isolate VT02162017-59 outer clamp protein and p13 genes. complete cds	Norway	2017	Salmo salar	Kibenge
MF946300	VT03022017-69	Ia	PRV1	Piscine orthoreovirus isolate VT03022017-69 outer clamp protein and p13 genes. complete cds	Canada	2017	Salmo salar	Kibenge
MF946301	VT03302017-99	Ia	PRV1	Piscine orthoreovirus isolate VT03302017-99 outer clamp protein and p13 genes. complete cds	Canada	2017	Salmo salar	Kibenge
MF946302	VT04282017A-108-10	Ia	PRV1	Piscine orthoreovirus isolate VT04282017A-108-10 outer clamp protein and p13 genes. complete cds	Canada	2017	Salmo salar	Kibenge
MG253811	NOR/060214	IIa	PRV3	Piscine orthoreovirus 3 strain NOR/060214 segment M2 mu 1 protein gene, complete cds	Norway	2013	Onchorynchus mykiss	Dhamotharan
MG253816	NOR/060214	IIa	PRV3	Piscine orthoreovirus 3 strain NOR/060214 segment S1 sigma 3 and p13 genes. complete cds	Norway	2014	Onchorynchus mykiss	Dhamotharan
MG720339	12-105_(58)	Ib	PRV1	Piscine orthoreovirus isolate 12-105_(58) F-Andoy 2012 Escaped segment S1 sigma 3 protein gene	Norway	2012	Salmo salar	Madhun
MG720340	12-112_(59)	Ib	PRV1	Piscine orthoreovirus isolate 12-112_(59) F-Andoy 2012 Escaped segment S1 sigma 3 protein gene	Norway	2012	Salmo salar	Madhun
MG720341	12-115_(60)	Ib	PRV1	Piscine orthoreovirus isolate 12-115_(60) F-Andoy 2012 Escaped segment S1 sigma 3 protein gene	Norway	2012	Salmo salar	Madhun
MG720342	22-6_(9)	Ib	PRV1	Piscine orthoreovirus isolate 22-6_(9) B-Nordkapp 2012 Escaped segment S1 sigma 3 protein gene	Norway	2012	Salmo salar	Madhun
MG720343	12-142_(61)	Ib	PRV1	Piscine orthoreovirus isolate 12-142_(61) F-Andoy 2012 Escaped segment S1 sigma 3 protein gene	Norway	2012	Salmo salar	Madhun
MG720344	38-172_(44)	Ib	PRV1	Piscine orthoreovirus isolate 38-172_(44) D-Manes 2012 Wild segment S1 sigma 3 protein gene. partial	Norway	2012	Salmo salar	Madhun
MG720345	8-032_(62)	Ib	PRV1	Piscine orthoreovirus isolate 8-032_(62) A-Bugoynes 2012 Escaped segment S1 sigma 3 protein gene	Norway	2012	Salmo salar	Madhun
MG720346	38-242_(43)	Ib	PRV1	Piscine orthoreovirus isolate 38-242_(43) D-Manes 2012 Escaped segment S1 sigma 3 protein gene	Norway	2012	Salmo salar	Madhun
MG720347	38-197_(48)	Ib	PRV1	Piscine orthoreovirus isolate 38-197_(48) D-Manes 2012 Escaped segment S1 sigma 3 protein gene	Norway	2012	Salmo salar	Madhun
MG720348	31-60_(54)	Ib	PRV1	Piscine orthoreovirus isolate 31-60_(54) C-Alta 2012 Escaped segment S1 sigma 3 protein gene. partial	Norway	2012	Salmo salar	Madhun
MG720349	8-093_(63)	Ib	PRV1	Piscine orthoreovirus isolate 8-093_(63) A-Bugoynes 2012 Wild segment S1 sigma 3 protein gene. partial	Norway	2012	Salmo salar	Madhun
MG720350	38-267_(36)	Ib	PRV1	Piscine orthoreovirus isolate 38-267_(36) D-Manes 2012 Wild segment S1 sigma 3 protein gene. partial	Norway	2012	Salmo salar	Madhun
MG720351	38-382_(18)	Ib	PRV1	Piscine orthoreovirus isolate 38-382_(18) D-Manes 2012 Escaped segment S1 sigma 3 protein gene	Norway	2012	Salmo salar	Madhun
MG720352	38-369_(42)	Ib	PRV1	Piscine orthoreovirus isolate 38-369_(42) D-Manes 2012 Wild segment S1 sigma 3 protein gene. partial	Norway	2012	Salmo salar	Madhun
MG720353	38-226_(45)	Ib	PRV1	Piscine orthoreovirus isolate 38-226_(45) D-Manes 2012 Escaped segment S1 sigma 3 protein gene	Norway	2012	Salmo salar	Madhun
MG720354	38-268_(13)	Ib	PRV1	Piscine orthoreovirus isolate 38-268_(13) D-Manes 2012 Wild segment S1 sigma 3 protein gene. partial	Norway	2012	Salmo salar	Madhun
MG720355	12-503_(15)	Ib	PRV1	Piscine orthoreovirus isolate 12-503_(15) E-Stamnes 2012 Escaped segment S1 sigma 3 protein gene	Norway	2012	Salmo salar	Madhun
MG720356	12-20B_(55)	Ia	PRV1	Piscine orthoreovirus isolate 12-20B_(55) F-Andoy 2012 Escaped segment S1 sigma 3 protein gene	Norway	2012	Salmo salar	Madhun
MG720357	12-501_(16)	Ia	PRV1	Piscine orthoreovirus isolate 12-501_(16) E-Stamnes 2012 Escaped segment S1 sigma 3 protein gene	Norway	2012	Salmo salar	Madhun
MG720358	38-181_(41)	Ia	PRV1	Piscine orthoreovirus isolate 38-181_(41) D-Manes 2012 Escaped segment S1 sigma 3 protein gene	Norway	2012	Salmo salar	Madhun
MG720359	38-225_(40)	Ia	PRV1	Piscine orthoreovirus isolate 38-225_(40) D-Manes 2012 Escaped segment S1 sigma 3 protein gene	Norway	2012	Salmo salar	Madhun
MG720360	12-31_(14)	Ia	PRV1	Piscine orthoreovirus isolate 12-31_(14) E-Stamnes 2012 Wild segment S1 sigma 3 protein gene. partial	Norway	2012	Salmo salar	Madhun
MG720361	38-368_(37)	Ia	PRV1	Piscine orthoreovirus isolate 38-368_(37) D-Manes 2012 Wild segment S1 sigma 3 protein gene. partial	Norway	2012	Salmo salar	Madhun
MG720362	38-182_(39)	Ia	PRV1	Piscine orthoreovirus isolate 38-182_(39) D-Manes 2012 Escaped segment S1 sigma 3 protein gene	Norway	2012	Salmo salar	Madhun
MG720363	38-183_(12)	Ia	PRV1	Piscine orthoreovirus isolate 38-183_(12) D-Manes 2012 Escaped segment S1 sigma 3 protein gene	Norway	2012	Salmo salar	Madhun
MG720364	38-196_(34)	Ia	PRV1	Piscine orthoreovirus isolate 38-196_(34) D-Manes 2012 Escaped segment S1 sigma 3 protein gene	Norway	2012	Salmo salar	Madhun
MG720365	31-02_(53)	Ia	PRV1	Piscine orthoreovirus isolate 31-02_(53) C-Alta 2012 Escaped segment S1 sigma 3 protein gene. partial	Norway	2012	Salmo salar	Madhun
MG720366	12-35_(57)	Ia	PRV1	Piscine orthoreovirus isolate 12-35_(57) E-Stamnes 2012 Wild segment S1 sigma 3 protein gene. partial	Norway	2012	Salmo salar	Madhun
MG720367	8-017_(17)	Ia	PRV1	Piscine orthoreovirus isolate 8-017_(17) A-Bugoynes 2012 Wild segment S1 sigma 3 protein gene. partial	Norway	2012	Salmo salar	Madhun
MG720368	31-72_(7)	Ia	PRV1	Piscine orthoreovirus isolate 31-72_(7) C-Alta 2012 Wild segment S1 sigma 3 protein gene. partial cds	Norway	2012	Salmo salar	Madhun
MG720369	38-207_(35)	Ia	PRV1	Piscine orthoreovirus isolate 38-207_(35) D-Manes 2012 Escaped segment S1 sigma 3 protein gene	Norway	2012	Salmo salar	Madhun
MG720370	22-14_(8)	Ia	PRV1	Piscine orthoreovirus isolate 22-14_(8) B-Nordkapp 2012 Wild segment S1 sigma 3 protein gene. partial	Norway	2012	Salmo salar	Madhun
MG720371	38-363_(46)	Ia	PRV1	Piscine orthoreovirus isolate 38-363_(46) D-Manes 2012 Escaped segment S1 sigma 3 protein gene	Norway	2012	Salmo salar	Madhun
MG720372	38-367_(11)	Ia	PRV1	Piscine orthoreovirus isolate 38-367_(11) D-Manes 2012 Escaped segment S1 sigma 3 protein gene	Norway	2012	Salmo salar	Madhun
MG720373	38-185_(33)	Ia	PRV1	Piscine orthoreovirus isolate 38-185_(33) D-Manes 2012 Escaped segment S1 sigma 3 protein gene	Norway	2012	Salmo salar	Madhun
MG720374	38-269_(38)	Ia	PRV1	Piscine orthoreovirus isolate 38-269_(38) D-Manes 2012 Wild segment S1 sigma 3 protein gene. partial	Norway	2012	Salmo salar	Madhun
MG720375	38-176_(47)	Ib	PRV1	Piscine orthoreovirus isolate 38-176_(47) D-Manes 2012 Escaped segment S1 sigma 3 protein gene	Norway	2012	Salmo salar	Madhun
MG720376	22-24_(10)	Ib	PRV1	Piscine orthoreovirus isolate 22-24_(10) B-Nordkapp 2012 Escaped segment S1 sigma 3 protein gene	Norway	2012	Salmo salar	Madhun
MG720377	12-11_(56)	Ia	PRV1	Piscine orthoreovirus isolate 12-11_(56) E-Stamnes 2012 Wild segment S1 sigma 3 protein gene. partial	Norway	2012	Salmo salar	Madhun
MG770490	DH773072017	IIa	PRV3	Piscine orthoreovirus isolate PRV_Om_DH773/775_072017 sigma 3 protein gene. partial cds	Germany	2017	Oncorhynchus mykiss	Adamek
MG983780	NOR/060214	IIa	PRV3	Piscine orthoreovirus 3 isolate NOR/060214 sigma 3 protein gene. partial cds	Norway	2013	Onchorynchus mykiss	Dhamotharan
MG983781	G1491	IIa	PRV3	Piscine orthoreovirus 3 isolate G1491 sigma 3 protein gene. partial cds	Scotland	2017	Onchorynchus mykiss	Dhamotharan
MG983782	DK/17-18918-13	IIa	PRV3	Piscine orthoreovirus 3 isolate DK/17-18918-13 sigma 3 protein gene. partial cds	Denmark	2017	Onchorynchus mykiss	Dhamotharan
MG983783	IT/17-211.3	IIa	PRV3	Piscine orthoreovirus 3 isolate IT/17-19266-27 sigma 3 protein gene. partial cds	Italy	2017	Salmo trutta	Dhamotharan
MG983784	IT/17-267	IIa	PRV3	Piscine orthoreovirus 3 isolate IT/17-19266-35 sigma 3 protein gene. partial cds	Italy	2017	Salmo trutta	Dhamotharan
MG983785	DK/17-18918-1	IIa	PRV3	Piscine orthoreovirus 3 isolate DK/17-18918-1 sigma 3 protein gene. partial cds	Denmark	2017	Onchorynchus mykiss	Dhamotharan
MG983786	DK/17-18918-6	IIa	PRV3	Piscine orthoreovirus 3 isolate DK/17-18918-6 sigma 3 protein gene. partial cds	Denmark	2017	Onchorynchus mykiss	Dhamotharan
MG983787	DH/747072017	IIa	PRV3	Piscine orthoreovirus 3 isolate DH/747072017 sigma 3 protein gene. partial cds	Germany	2017	Onchorynchus mykiss	Dhamotharan
MH093952	A.3.2-36_G609	Ia	PRV1	Piscine orthoreovirus isolate A.3.2-36_G609 outer shell gene, complete cds	Canada	2011	Salmo salar	Di Cicco
MH093953	A.3.2-69_G531	Ia	PRV1	Piscine orthoreovirus isolate A.3.2-69_G531 outer shell gene, complete cds	Canada	2011	Salmo salar	Di Cicco
MH093954	A.3.2-153_G808	Ia	PRV1	Piscine orthoreovirus isolate A.3.2-153_G808 outer shell gene, complete cds	Canada	2013	Salmo salar	Di Cicco
MH093955	A.3.3-19_G744	Ia	PRV1	Piscine orthoreovirus isolate A.3.3-19_G744 outer shell gene, complete cds	Canada	2012	Salmo salar	Di Cicco
MH093956	A.3.3-133_G444	Ia	PRV1	Piscine orthoreovirus isolate A.3.3-133_G444 outer shell gene, complete cds	Canada	2012	Salmo salar	Di Cicco
MH093957	A.3.5-168_G860	Ia	PRV1	Piscine orthoreovirus isolate A.3.5-168_G860 outer shell gene, complete cds	Canada	2013	Salmo salar	Di Cicco
MH093958	P.2-1_G577	Ia	PRV1	Piscine orthoreovirus isolate P.2-1_G577 outer shell gene, complete cds	Canada	2011	Oncorhynchus tshawytscha	Di Cicco
MH093959	P.2-3_G460	Ia	PRV1	Piscine orthoreovirus isolate P.2-3_G460 outer shell gene, complete cds	Canada	2013	Oncorhynchus tshawytscha	Di Cicco
MH093960	P.2-95_G383	Ia	PRV1	Piscine orthoreovirus isolate P.2-95_G383 outer shell gene, complete cds	Canada	2013	Oncorhynchus tshawytscha	Di Cicco
MH093961	P.2-99_G772	Ia	PRV1	Piscine orthoreovirus isolate P.2-99_G772 outer shell gene, complete cds	Canada	2013	Oncorhynchus tshawytscha	Di Cicco
MH093962	P.3-3_G729	Ia	PRV1	Piscine orthoreovirus isolate P.3-3_G729 outer shell gene, complete cds	Canada	2012	Oncorhynchus tshawytscha	Di Cicco
MH093963	P.3-37_G446	Ia	PRV1	Piscine orthoreovirus isolate P.3-37_G446 outer shell gene, complete cds	Canada	2013	Oncorhynchus tshawytscha	Di Cicco
MH093964	P.3-120_G417	Ia	PRV1	Piscine orthoreovirus isolate P.3-120_G417 outer shell gene, complete cds	Canada	2012	Oncorhynchus tshawytscha	Di Cicco
MH093978	A.3.2-36_G609	Ia	PRV1	Piscine orthoreovirus isolate A.3.2-36_G609 outer clamp gene. partial cds	Canada	2011	Salmo salar	Di Cicco
MH093979	A.3.2-69_G531	Ia	PRV1	Piscine orthoreovirus isolate A.3.2-69_G531 outer clamp gene. partial cds	Canada	2011	Salmo salar	Di Cicco
MH093980	A.3.2-153_G808	Ia	PRV1	Piscine orthoreovirus isolate A.3.2-153_G808 outer clamp gene. partial cds	Canada	2013	Salmo salar	Di Cicco
MH093981	A.3.3-19_G744	Ia	PRV1	Piscine orthoreovirus isolate A.3.3-19_G744 outer clamp gene. partial cds	Canada	2012	Salmo salar	Di Cicco
MH093982	A.3.3-133_G444	Ia	PRV1	Piscine orthoreovirus isolate A.3.3-133_G444 outer clamp gene. partial cds	Canada	2012	Salmo salar	Di Cicco
MH093983	A.3.5-168_G860	Ia	PRV1	Piscine orthoreovirus isolate A.3.5-168_G860 outer clamp gene. partial cds	Canada	2013	Salmo salar	Di Cicco
MH093984	P.2-1_G577	Ia	PRV1	Piscine orthoreovirus isolate P.2-1_G577 outer clamp gene. partial cds	Canada	2011	Oncorhynchus tshawytscha	Di Cicco
MH093985	P.2-3_G460	Ia	PRV1	Piscine orthoreovirus isolate P.2-3_G460 outer clamp gene. partial cds	Canada	2013	Oncorhynchus tshawytscha	Di Cicco
MH093986	P.2-95_G383	Ia	PRV1	Piscine orthoreovirus isolate P.2-95_G383 outer clamp gene. partial cds	Canada	2013	Oncorhynchus tshawytscha	Di Cicco
MH093987	P.2-99_G772	Ia	PRV1	Piscine orthoreovirus isolate P.2-99_G772 outer clamp gene. partial cds	Canada	2013	Oncorhynchus tshawytscha	Di Cicco
MH093988	P.3-3_G729	Ia	PRV1	Piscine orthoreovirus isolate P.3-3_G729 outer clamp gene. partial cds	Canada	2012	Oncorhynchus tshawytscha	Di Cicco
MH093989	P.3-37_G446	Ia	PRV1	Piscine orthoreovirus isolate P.3-37_G446 outer clamp gene. partial cds	Canada	2013	Oncorhynchus tshawytscha	Di Cicco
MH093990	P.3-120_G417	Ia	PRV1	Piscine orthoreovirus isolate P.3-120_G417 outer clamp gene. partial cds	Canada	2012	Oncorhynchus tshawytscha	Di Cicco
MH215517	VT01192018-07	Ia	PRV1	Piscine orthoreovirus isolate VT01192018-07 sigma 3 protein and p13 genes. complete cds	USA	2017	Salmo salar	Kibenge
MH215518	VT01192018-16	Ia	PRV1	Piscine orthoreovirus isolate VT01192018-16 sigma 3 protein and p13 genes. complete cds	USA	2017	Salmo salar	Kibenge
MH215519	VT01192018-21	Ia	PRV1	Piscine orthoreovirus isolate VT01192018-21 sigma 3 protein and p13 genes. complete cds	USA	2017	Salmo salar	Kibenge
MH215520	VT01192018-26	Ia	PRV1	Piscine orthoreovirus isolate VT01192018-26 sigma 3 protein and p13 genes. complete cds	USA	2017	Salmo salar	Kibenge
MH215521	VT01192018-33	Ia	PRV1	Piscine orthoreovirus isolate VT01192018-33 sigma 3 protein and p13 genes. complete cds	USA	2017	Salmo salar	Kibenge
MH215522	VT01192018-43	Ia	PRV1	Piscine orthoreovirus isolate VT01192018-43 sigma 3 protein and p13 genes. complete cds	USA	2017	Salmo salar	Kibenge
MH215523	VT01192018-48	Ia	PRV1	Piscine orthoreovirus isolate VT01192018-48 sigma 3 protein and p13 genes. complete cds	USA	2017	Salmo salar	Kibenge
MH215524	VT01192018-52	Ia	PRV1	Piscine orthoreovirus isolate VT01192018-52 sigma 3 protein and p13 genes. complete cds	USA	2017	Salmo salar	Kibenge
MH215525	VT01192018-53	Ia	PRV1	Piscine orthoreovirus isolate VT01192018-53 sigma 3 protein and p13 genes. complete cds	USA	2017	Salmo salar	Kibenge
MH215526	VT10252017-405	Ia	PRV1	Piscine orthoreovirus isolate VT10252017-405 sigma 3 protein and p13 genes. complete cds	Canada	2017	Salmo salar	Kibenge
MH215527	VT11022017-423	Ia	PRV1	Piscine orthoreovirus isolate VT11022017-423 sigma 3 protein and p13 genes. complete cds	Canada	2017	Salmo salar	Kibenge
MH229780	ADLPRV3	IIa	PRV3	Piscine orthoreovirus 3 isolate ADLPRV3 segment M2, complete sequence	Chile	2017	Oncorhynchus kisutch	Bohle
MH229785	ADLPRV3	IIa	PRV3	Piscine orthoreovirus 3 isolate ADLPRV3 segment S1, complete sequence	Chile	2017	Oncorhynchus kisutch	Bohle
MH347363	16-005	unknown	unknown	Piscine orthoreovirus isolate 16-005 segment M2 outer capsid shell gene, complete cds.	Canada	2016	Salmo salar	Polinski
MH347364	16-005	unknown	unknown	Piscine orthoreovirus isolate 16-005 segment M2 outer capsid shell gene, complete cds	Canada	2016	Salmo salar	Polinski
MH347365	16-005	Ia	PRV1	Piscine orthoreovirus isolate 16-005 segment S1 outer capsid coat and nonstructural protein P13 genes, complete cds.	Canada	2016	Salmo salar	Polinski
MH347373	16-011	unknown	unknown	Piscine orthoreovirus isolate 16-011 segment M2 outer capsid shell gene, complete cds	Canada	2016	Salmo salar	Polinski
MH347375	16-011	Ia	PRV1	Piscine orthoreovirus isolate 16-011 segment S1 outer capsid coat and nonstructural protein P13 genes, complete cds.	Canada	2016	Salmo salar	Polinski
MH513870	Kuehn-PRV2	IIa	PRV3	Piscine orthoreovirus isolate S1 outer clamp protein gene. partial cds	Germany	2008	Salmo salar	Kuehn
MH558129	VT04202018-119	Ia	PRV1	Piscine orthoreovirus isolate VT04202018-119 segment S1 outer clamp protein and nonstructural protein	USA	2018	Salmo salar	Kibenge
MH558130	VT04202018-121	Ia	PRV1	Piscine orthoreovirus isolate VT04202018-121 segment S1 outer clamp protein and nonstructural protein	USA	2018	Salmo salar	Kibenge
MH558131	VT04202018-123	Ia	PRV1	Piscine orthoreovirus isolate VT04202018-123 segment S1 outer clamp protein and nonstructural protein	USA	2018	Salmo salar	Kibenge
MH558132	VT04202018-124	Ia	PRV1	Piscine orthoreovirus isolate VT04202018-124 segment S1 outer clamp protein and nonstructural protein	USA	2017	Salmo salar	Kibenge
MH558133	VT04202018-129	Ia	PRV1	Piscine orthoreovirus isolate VT04202018-129 segment S1 outer clamp protein and nonstructural protein	Iceland	2018	Salmo salar	Kibenge
MH581192	VT05252017-236	Ia	PRV1	Piscine orthoreovirus isolate VT05252017-236 outer clamp protein and non-structural protein genes	Canada	2017	Salmo salar	Kibenge
MH581193	VT06092017-347	Ia	PRV1	Piscine orthoreovirus isolate VT06092017-347 outer clamp protein and non-structural protein genes	Canada	2017	Salmo salar	Kibenge
MH581194	VT06092017-348	Ia	PRV1	Piscine orthoreovirus isolate VT06092017-348 outer clamp protein and non-structural protein genes	Canada	2017	Salmo salar	Kibenge
MH581195	VT10042017-382	Ia	PRV1	Piscine orthoreovirus isolate VT10042017-382 outer clamp protein and non-structural protein genes	Canada	2017	Salmo salar	Kibenge
MH581196	VT10042017-383	Ia	PRV1	Piscine orthoreovirus isolate VT10042017-383 outer clamp protein and non-structural protein genes	Canada	2017	Salmo salar	Kibenge
MH581197	VT10252017-421	Ia	PRV1	Piscine orthoreovirus isolate VT10252017-421 outer clamp protein and non-structural protein genes	Canada	2017	Oncorhynchus tshawytscha	Kibenge
MH581198	VT10252017-422	Ia	PRV1	Piscine orthoreovirus isolate VT10252017-422 outer clamp protein and non-structural protein genes	Canada	2017	Oncorhynchus tshawytscha	Kibenge
MH581199	VT11302017-453	Ia	PRV1	Piscine orthoreovirus isolate VT11302017-453 outer clamp protein and non-structural protein genes	Canada	2017	Salmo salar	Kibenge
MH581200	VT11302017-454	Ia	PRV1	Piscine orthoreovirus isolate VT11302017-454 outer clamp protein and non-structural protein genes	Canada	2017	Salmo salar	Kibenge
MH581201	VT03022018-66	Ia	PRV1	Piscine orthoreovirus isolate VT03022018-66 outer clamp protein and non-structural protein genes	Canada	2018	Salmo salar	Kibenge
MH581202	VT03022018-67	Ia	PRV1	Piscine orthoreovirus isolate VT03022018-67 outer clamp protein and non-structural protein genes	Canada	2018	Salmo salar	Kibenge
MH581203	VT03022018-68	Ia	PRV1	Piscine orthoreovirus isolate VT03022018-68 outer clamp protein and non-structural protein genes	Canada	2018	Salmo salar	Kibenge
MH581204	VT03262018-75-27	Ia	PRV1	Piscine orthoreovirus isolate VT03262018-75-27 outer clamp protein and non-structural protein genes	Canada	2018	Salmo salar	Kibenge
MH581205	VT03262018-77	Ia	PRV1	Piscine orthoreovirus isolate VT03262018-77 outer clamp protein and non-structural protein genes	Canada	2018	Salmo salar	Kibenge
MH581206	VT03262018-83	Ia	PRV1	Piscine orthoreovirus isolate VT03262018-83 outer clamp protein and non-structural protein genes	Canada	2018	Salmo salar	Kibenge

MH581207	VT03262018-84	Ia	PRV1	Piscine orthoreovirus isolate VT03262018-84 outer clamp protein and non-structural protein genes	Canada	2018	Salmo salar	Kibenge
MH581208	VT03262018-88	Ia	PRV1	Piscine orthoreovirus isolate VT03262018-88 outer clamp protein and non-structural protein genes	Canada	2018	Salmo salar	Kibenge
MH581209	VT04062018-105	Ia	PRV1	Piscine orthoreovirus isolate VT04062018-105 outer clamp protein and non-structural protein genes	Canada	2018	Salmo salar	Kibenge
MH581210	VT04062018-106	Ia	PRV1	Piscine orthoreovirus isolate VT04062018-106 outer clamp protein and non-structural protein genes	Canada	2018	Salmo salar	Kibenge
MH581211	VT04062018-108	Ia	PRV1	Piscine orthoreovirus isolate VT04062018-108 outer clamp protein and non-structural protein genes	Canada	2018	Salmo salar	Kibenge
MH581212	VT04062018-109	Ia	PRV1	Piscine orthoreovirus isolate VT04062018-109 outer clamp protein and non-structural protein genes	Canada	2018	Salmo salar	Kibenge
MK052922	13(P2)	IIa	PRV3	Piscine orthoreovirus isolate 13(P2) segment S1, complete sequence	Chile	2018	Oncorhynchus kisutch	Cartagena
MK052923	14(P2)	IIa	PRV3	Piscine orthoreovirus isolate 14(P2) segment S1, complete sequence	Chile	2018	Oncorhynchus kisutch	Cartagena
MK052924	15(P2)	IIa	PRV3	Piscine orthoreovirus isolate 15(P2) segment S1, complete sequence	Chile	2018	Oncorhynchus kisutch	Cartagena
MK052925	17(P5)	Ib	PRV1	Piscine orthoreovirus isolate 17(P5) segment S1, complete sequence.	Chile	2018	Oncorhynchus kisutch	Cartagena
MK052926	18(P5)	Ib	PRV1	Piscine orthoreovirus isolate 18(P5) segment S1, complete sequence.	Chile	2018	Oncorhynchus kisutch	Cartagena
MK052927	19(P2)	IIa	PRV3	Piscine orthoreovirus isolate 19(P2) segment S1, complete sequence.	Chile	2018	Oncorhynchus kisutch	Cartagena
MK052928	23(P2)	IIa	PRV3	Piscine orthoreovirus isolate 23(P2) segment S1, complete sequence	Chile	2018	Oncorhynchus kisutch	Cartagena
MK052929	25(P5)	Ib	PRV1	Piscine orthoreovirus isolate 25(P5) segment S1, complete sequence.	Chile	2018	Oncorhynchus kisutch	Cartagena
MK052930	27(P5)	Ib	PRV1	Piscine orthoreovirus isolate 27(P5) segment S1, complete sequence	Chile	2018	Oncorhynchus kisutch	Cartagena
MK052931	28(P5)	Ib	PRV1	Piscine orthoreovirus isolate 28(P5) segment S1, complete sequence.	Chile	2018	Oncorhynchus kisutch	Cartagena
MK286552	Kuehn-PRV2	IIa	PRV3	Piscine orthoreovirus outer shell protein gene, partial cds	Germany	2008	Salmo trutta fario	Kuehn
MK286554	Kuehn-PRV2	IIa	PRV3	Piscine orthoreovirus outer clamp protein gene. partial cds	Germany	2008	Salmo trutta fario	Kuehn
MK368744	DK-14-15958-4	Ia	PRV1	Piscine orthoreovirus isolate DK-14-15958-4 sigma 3 protein gene. complete cds	Denmark	2014	Salmo salar	Vendramin
MK368745	DK-2013-50-288-63	Ia	PRV1	Piscine orthoreovirus isolate DK-2013-50-288-63 sigma 3 protein gene. partial cds	Denmark	2013	Salmo salar	Vendramin
MK368746	DK-2013-50-259-10	Ia	PRV1	Piscine orthoreovirus isolate DK-2013-50-259-10 sigma 3 protein gene. partial cds	Denmark	2013	Salmo salar	Vendramin
MK368747	DK-2013-50-282-20	Ia	PRV1	Piscine orthoreovirus isolate DK-2013-50-282-20 sigma 3 protein gene. partial cds	Denmark	2013	Salmo salar	Vendramin
MK368748	DK-2013-50-282-11	Ia	PRV1	Piscine orthoreovirus isolate DK-2013-50-282-11 sigma 3 protein gene. partial cds	Denmark	2013	Salmo salar	Vendramin
MK368749	DK-14-16407-1	Ia	PRV1	Piscine orthoreovirus isolate DK-14-16407-1 sigma 3 protein gene. partial cds	Denmark	2014	Salmo salar	Vendramin
MK368750	DK-2013-50-288-65	Ia	PRV1	Piscine orthoreovirus isolate DK-2013-50-288-65 sigma 3 protein gene. partial cds	Denmark	2013	Salmo salar	Vendramin
MK368751	DK-15-23576-37	Ia	PRV1	Piscine orthoreovirus isolate DK-15-23576-37 sigma 3 protein gene. partial cds	Denmark	2015	Salmo salar	Vendramin
MK368752	DK-14-15550-2	Ib	PRV1	Piscine orthoreovirus isolate DK-14-15550-2 sigma 3 protein gene. partial cds	Denmark	2015	Salmo salar	Vendramin
MK368753	DK-14-16407-3	Ib	PRV1	Piscine orthoreovirus isolate DK-14-16407-3 sigma 3 protein gene. complete cds	Denmark	2014	Salmo salar	Vendramin
MK368754	DK-15-23478-20	Ib	PRV1	Piscine orthoreovirus isolate DK-15-23478-20 sigma 3 protein gene. partial cds	Denmark	2015	Salmo salar	Vendramin
MK368755	SW-17-1611-24	Ib	PRV1	Piscine orthoreovirus isolate SW-17-1611-24 sigma 3 protein gene. partial cds	Sweden	2017	Salmo salar	Vendramin
MK368756	IR-17-13316-6	Ia	PRV1	Piscine orthoreovirus isolate IR-17-13316-6 sigma 3 protein gene. partial cds	Ireland	2017	Salmo salar	Vendramin
MK368757	IR-17-6222-57	Ia	PRV1	Piscine orthoreovirus isolate IR-17-6222-57 sigma 3 protein gene. partial cds	Ireland	2017	Salmo salar	Vendramin
MK368758	F211-893-59	Ia	PRV1	Piscine orthoreovirus isolate F211-893-59 sigma 3 protein gene. partial cds	Faroe Islands	2011	Salmo salar	Vendramin
MK368759	F211-516-8	Ia	PRV1	Piscine orthoreovirus isolate F211-516-8 sigma 3 protein gene. partial cds	Faroe Islands	2011	Salmo salar	Vendramin
MK675826	NOR-1997	Ib	PRV1	Piscine orthoreovirus isolate NOR-1997 segment M2 mu1 gene, partial cds.	Norway	1997	Salmo salar	Dhamotharan
MK675828	NOR-1997	Ib	PRV1	Piscine orthoreovirus isolate NOR-1997 segment S1 sigma 3 and p13 genes. complete cds	Norway	1997	Salmo salar	Dhamotharan
MK675836	NOR-2005/TT	Ib	PRV1	Piscine orthoreovirus isolate NOR-2005/TT segment M2 mu1 gene, complete cds.	Norway	2005	Salmo salar	Dhamotharan
MK675838	NOR-2005/TT	Ib	PRV1	Piscine orthoreovirus isolate NOR-2005/TT segment S1 sigma 3 and p13 genes. complete cds	Norway	2005	Salmo salar	Dhamotharan
MK675846	NOR-2015/MS	Ib	PRV1	Piscine orthoreovirus isolate NOR-2015/MS segment M2 mu1 gene, complete cds	Norway	2015	Salmo salar	Dhamotharan
MK675848	NOR-2015/MS	Ib	PRV1	Piscine orthoreovirus isolate NOR-2015/MS segment S1 sigma 3 and p13 genes. complete cds	Norway	2015	Salmo salar	Dhamotharan
MK675856	NOR-2015/SSK	Ib	PRV1	Piscine orthoreovirus isolate NOR-2015/SSK segment M2 mu1 gene, complete cds	Norway	2015	Salmo salar	Dhamotharan
MK675858	NOR-2015/SSK	Ib	PRV1	Piscine orthoreovirus isolate NOR-2015/SSK segment S1 sigma 3 and p13 genes. complete cds	Norway	2015	Salmo salar	Dhamotharan
MK675866	NOR-1988	Ia	PRV1	Piscine orthoreovirus isolate NOR-1988 segment M2 mu1 gene, complete cds	Norway	1998	Salmo salar	Dhamotharan
MK675868	NOR-1988	Ia	PRV1	Piscine orthoreovirus isolate NOR-1988 segment S1 sigma 3 and p13 genes. complete cds	Norway	1998	Salmo salar	Dhamotharan
MK675876	FO/1978/15	Ia	PRV1	Piscine orthoreovirus isolate FO/1978/15 segment M2 mu1 gene, complete cds	Denmark	2015	Salmo salar	Vendramin
MK675878	FO/1978/15	Ia	PRV1	Piscine orthoreovirus isolate FO/1978/15 segment S1 sigma 3 and p13 genes. complete cds	Denmark	2015	Salmo salar	Vendramin
MK675886	FO/41/16	Ia	PRV1	Piscine orthoreovirus isolate FO/41/16 segment M2 mu1 gene, complete cds	Denmark	2016	Salmo salar	Vendramin
MK675888	FO/41/16	Ia	PRV1	Piscine orthoreovirus isolate FO/41/16 segment S1 sigma 3 and p13 genes. complete cds	Denmark	2016	Salmo salar	Vendramin
MN106287	J3542	unknown	unknown	Piscine orthoreovirus isolate J3542 L1 gene, complete cds.	Greenland	2017	Salmo salar	Teffer
MN106288	J3575	unknown	unknown	Piscine orthoreovirus isolate J3575 L1 gene, complete cds.	Greenland	2017	Salmo salar	Teffer
MN106289	J3611	unknown	unknown	Piscine orthoreovirus isolate J3611 L1 gene, complete cds.	Greenland	2016	Salmo salar	Teffer
MN106290	J3542	unknown	unknown	Piscine orthoreovirus isolate J3542 L2 gene, complete cds.	Greenland	2017	Salmo salar	Teffer
MN106291	J3575	unknown	unknown	Piscine orthoreovirus isolate J3575 L2 gene, complete cds.	Greenland	2017	Salmo salar	Teffer
MN106292	J3611	unknown	unknown	Piscine orthoreovirus isolate J3611 L2 gene, complete cds.	Greenland	2016	Salmo salar	Teffer
MN106293	J3542	unknown	unknown	Piscine orthoreovirus isolate J3542 L3 gene, complete cds.	Greenland	2017	Salmo salar	Teffer
MN106294	J3575	unknown	unknown	Piscine orthoreovirus isolate J3575 L3 gene, complete cds.	Greenland	2017	Salmo salar	Teffer
MN106295	J3611	unknown	unknown	Piscine orthoreovirus isolate J3611 L3 gene, complete cds.	Greenland	2016	Salmo salar	Teffer
MN106296	J3542	unknown	unknown	Piscine orthoreovirus isolate J3542 M1 gene, complete cds.	Greenland	2017	Salmo salar	Teffer
MN106297	J3575	unknown	unknown	Piscine orthoreovirus isolate J3575 M1 gene, complete cds.	Greenland	2017	Salmo salar	Teffer
MN106298	J3611	unknown	unknown	Piscine orthoreovirus isolate J3611 M1 gene, complete cds.	Greenland	2016	Salmo salar	Teffer
MN106299	J3542	unknown	unknown	Piscine orthoreovirus isolate J3542 M2 gene, complete cds.	Greenland	2017	Salmo salar	Teffer
MN106300	J3575	unknown	unknown	Piscine orthoreovirus isolate J3575 M2 gene, complete cds.	Greenland	2017	Salmo salar	Teffer
MN106301	J3611	unknown	unknown	Piscine orthoreovirus isolate J3611 M2 gene, complete cds.	Greenland	2016	Salmo salar	Teffer
MN106302	J3542	unknown	unknown	Piscine orthoreovirus isolate J3542 M3 gene, complete cds.	Greenland	2017	Salmo salar	Teffer
MN106303	J3575	unknown	unknown	Piscine orthoreovirus isolate J3575 M3 gene, complete cds.	Greenland	2017	Salmo salar	Teffer
MN106304	J3611	unknown	unknown	Piscine orthoreovirus isolate J3611 M3 gene, complete cds.	Greenland	2016	Salmo salar	Teffer
MN106305	J3542	unknown	unknown	Piscine orthoreovirus isolate J3542 S1 gene, complete cds.	Greenland	2017	Salmo salar	Teffer
MN106306	J3575	unknown	unknown	Piscine orthoreovirus isolate J3575 S1 gene, complete cds.	Greenland	2017	Salmo salar	Teffer
MN106307	J3611	unknown	unknown	Piscine orthoreovirus isolate J3611 S1 gene, complete cds.	Greenland	2016	Salmo salar	Teffer
MN106308	J3542	unknown	unknown	Piscine orthoreovirus isolate J3542 S2 gene, complete cds.	Greenland	2017	Salmo salar	Teffer
MN106309	J3575	unknown	unknown	Piscine orthoreovirus isolate J3575 S2 gene, complete cds.	Greenland	2017	Salmo salar	Teffer
MN106310	J3611	unknown	unknown	Piscine orthoreovirus isolate J3611 S2 gene, complete cds.	Greenland	2016	Salmo salar	Teffer
MN106311	J3542	unknown	unknown	Piscine orthoreovirus isolate J3542 S3 gene, complete cds.	Greenland	2017	Salmo salar	Teffer
MN106312	j3575	unknown	unknown	Piscine orthoreovirus isolate J3575 S3 gene, complete cds.	Greenland	2017	Salmo salar	Teffer
MN106313	J3611	unknown	unknown	Piscine orthoreovirus isolate J3611 S3 gene, complete cds.	Greenland	2016	Salmo salar	Teffer
MN106314	J3542	unknown	unknown	Piscine orthoreovirus isolate J3542 S4 gene, partial cds	Greenland	2018	Salmo salar	Teffer
MN106315	J3575	unknown	unknown	Piscine orthoreovirus isolate J3575 S4 gene, partial cds	Greenland	2017	Salmo salar	Teffer
MN106316	J3611	unknown	unknown	Piscine orthoreovirus isolate J3611 S4 gene, partial cds	Greenland	2016	Salmo salar	Teffer
MN337614	2019-50-212	unknown	unknown	Piscine orthoreovirus 3 isolate 2019-50-212 S3 gene, partial cds.	Norway	2017	Onchorynchus mykiss	Moldal
NC036472	CGA280-05	Ib	PRV1	Piscine reovirus isolate CGA280-05 outer clamp protein and non-structural protein gene, segment S1, complete cds.	Chile	2011	Salmo salar	Kibenge
