# Supplementary material for: Extensive Phylogenetic Analysis of Piscine Orthoreovirus Genomic Sequences Shows the Robustness of Subgenotype Classification
Source: Pathogens. 2021 Jan 7;10(1):41. doi: 10.3390/pathogens10010041 (PMC7825714; doi:10.3390/pathogens10010041)
Supplement: Supplementary file 1 [file pathogens-10-00041-s001.zip › Supplementary folder/pathogens_Supplementary Table S2_December 13 2020.pdf]

Supplementary Table S2. GenBank accession numbers and metadata of PRV segments S1 and M2 sequences listed under the Tax ID: txid1157337 in the NCBI GenBank database to date (May 2020) used in this study

GenBank Accession Number	PRV isolate	PRV subgenotype (Kibenge et al., 2013; Godoy *et al.,* 2016)	PRV subtype (Dhamotaran *et al.,* 2018)	PRV Description	Country	Collection_date (Year)	Host	Primary Author
AF059720	OUTGROUP			Avian orthoreovirus strain 176 segment S3 major outer capsid	Canada	1999	Avian	Duncan
GU994016	Salmo/GP-2010/NOR	Ib	PRV1	Reovirus sp. Salmo/GP-2010/NOR segment M2, partial sequence	Norway	2010	*Salmo salar*	Palacios
GU994022	Salmo/GP-2010/NOR	Ib	PRV1	Reovirus sp. Salmo/GP-2010/NOR segment S1. partial sequence	Norway	2010	*Salmo salar*	Palacios
HG329842	35 Bjoreio	Ia	PRV1	Piscine reovirus partial mRNA for Sigma 3. segment S1. isolate 35 Bjoreio 2009 Wild	Norway	2009	*Salmo salar*	Garseth
HG329843	45 Eira	Ia	PRV1	Piscine reovirus partial mRNA for Sigma 3. segment S1. isolate 45 Eira 2009 Hatchery-reared	Norway	2009	*Salmo salar*	Garseth
HG329844	61 Eira	Ib	PRV1	Piscine reovirus partial mRNA for Sigma 3. segment S1. isolate 61 Eira 2009 Hatchery reared	Norway	2009	*Salmo salar*	Garseth
HG329845	81 Etne	Ib	PRV1	Piscine reovirus partial mRNA for Sigma 3. segment S1. isolate 81 Etne 2009 Wild	Norway	2009	*Salmo salar*	Garseth
HG329846	90 Etne	Ib	PRV1	Piscine reovirus partial mRNA for Sigma 3. segment S1. isolate 90 Etne 2009 Wild	Norway	2009	*Salmo salar*	Garseth
HG329847	93 Etne	Ib	PRV1	Piscine reovirus partial mRNA for Sigma 3. segment S1. isolate 93 Etne 2009 Wild	Norway	2009	*Salmo salar*	Garseth
HG329848	131 Gaula	Ia	PRV1	Piscine reovirus partial mRNA for Sigma 3. segment S1. isolate 131 Gaula 2009 Wild	Norway	2009	*Salmo salar*	Garseth
HG329849	182 Hestdal	Ia	PRV1	Piscine reovirus partial mRNA for Sigma 3. segment S1. isolate 182 Hestdal 2009 Wild	Norway	2009	*Salmo salar*	Garseth
HG329850	187 Hestdal	Ia	PRV1	Piscine reovirus partial mRNA for Sigma 3. segment S1. isolate 187 Hestdal 2009 Wild	Norway	2009	*Salmo salar*	Garseth
HG329851	190 Hestdal	Ia	PRV1	Piscine reovirus partial mRNA for Sigma 3. segment S1. isolate 190 Hestdal 2009 Wild	Norway	2009	*Salmo salar*	Garseth
HG329852	211 Mandal	Ia	PRV1	Piscine reovirus partial mRNA for Sigma 3. segment S1. isolate 211 Mandal 2009 Wild	Norway	2009	*Salmo salar*	Garseth
HG329853	220 Mandal	Ib	PRV1	Piscine reovirus partial mRNA for Sigma 3. segment S1. isolate 220 Mandal 2009 Wild	Norway	2009	*Salmo salar*	Garseth
HG329854	246 Mandal	Ia	PRV1	Piscine reovirus partial mRNA for Sigma 3. segment S1. isolate 246 Mandal 2009 Wild	Norway	2009	*Salmo salar*	Garseth
HG329855	261 Nidelv	Ib	PRV1	Piscine reovirus partial mRNA for Sigma 3. segment S1. isolate 261 Nidelv 2009 Escaped farmed	Norway	2009	*Salmo salar*	Garseth
HG329856	273 Skibotn	Ib	PRV1	Piscine reovirus partial mRNA for Sigma 3. segment S1. isolate 273 Skibotn 2009 Wild	Norway	2009	*Salmo salar*	Garseth
HG329857	283 Skjomen	Ib	PRV1	Piscine reovirus partial mRNA for Sigma 3. segment S1. isolate 283 Skjomen 2009 Escaped farmed	Norway	2009	*Salmo salar*	Garseth
HG329858	284 Stjordal	Ia	PRV1	Piscine reovirus partial mRNA for Sigma 3. segment S1. isolate 284 Stjordal 2009 Wild	Norway	2009	*Salmo salar*	Garseth
HG329859	307 Stjordal	Ia	PRV1	Piscine reovirus partial mRNA for Sigma 3. segment S1. isolate 307 Stjordal 2009 Wild	Norway	2009	*Salmo salar*	Garseth
HG329860	318 Storelva Holt	Ib	PRV1	Piscine reovirus partial mRNA for Sigma 3. segment S1. isolate 318 Storelva Holt 2009 Wild	Norway	2009	*Salmo salar*	Garseth
HG329861	338 Surna	Ib	PRV1	Piscine reovirus partial mRNA for Sigma 3. segment S1. isolate 338 Surna 2009 Escaped farmed	Norway	2009	*Salmo salar*	Garseth
HG329862	350 Surna	Ib	PRV1	Piscine reovirus partial mRNA for Sigma 3. segment S1. isolate 350 Surna 2009 Escaped farmed	Norway	2009	*Salmo salar*	Garseth
HG329863	407 Vosso	Ia	PRV1	Piscine reovirus partial mRNA for Sigma 3. segment S1. isolate 407 Vosso 2009 Wild	Norway	2009	*Salmo salar*	Garseth
HG329864	411 Vosso	Ib	PRV1	Piscine reovirus partial mRNA for Sigma 3. segment S1. isolate 41 Vosso 2009 Wild	Norway	2009	*Salmo salar*	Garseth
HG329865	412 Vosso	Ib	PRV1	Piscine reovirus partial mRNA for Sigma 3. segment S1. isolate 412 Vosso 2009 Hatchery-reared	Norway	2009	*Salmo salar*	Garseth
HG329866	414 Vosso	Ib	PRV1	Piscine reovirus partial mRNA for Sigma 3. segment S1. isolate 414 Vosso 2009 Hatchery-reared	Norway	2009	*Salmo salar*	Garseth
HG329867	438 Alta	Ib	PRV1	Piscine reovirus partial mRNA for Sigma 3. segment S1. isolate 438 Alta 2008 Wild	Norway	2008	*Salmo salar*	Garseth
HG329868	445 Alta	Ia	PRV1	Piscine reovirus partial mRNA for Sigma 3. segment S1. isolate 445 Alta 2008 Wild	Norway	2008	*Salmo salar*	Garseth
HG329869	470 Drevja	Ia	PRV1	Piscine reovirus partial mRNA for Sigma 3. segment S1. isolate 470 Drevja 2008 Wild	Norway	2008	*Salmo salar*	Garseth
HG329870	491 Eira	Ib	PRV1	Piscine reovirus partial mRNA for Sigma 3. segment S1. isolate 491 Eira 2008 Wild	Norway	2008	*Salmo salar*	Garseth
HG329871	517 Eira	Ia	PRV1	Piscine reovirus partial mRNA for Sigma 3. segment S1. isolate 517 Eira 2008 Wild	Norway	2008	*Salmo salar*	Garseth
HG329872	522 Ekso	Ib	PRV1	Piscine reovirus partial mRNA for Sigma 3. segment S1. isolate 522 Ekso 2008 Wild	Norway	2008	*Salmo salar*	Garseth
HG329873	555 Fusta	Ib	PRV1	Piscine reovirus partial mRNA for Sigma 3. segment S1. isolate 555 Fusta 2008 Wild	Norway	2008	*Salmo salar*	Garseth
HG329874	565 Gaula	Ib	PRV1	Piscine reovirus partial mRNA for Sigma 3. segment S1. isolate 565 Gaula 2008 Wild	Norway	2008	*Salmo salar*	Garseth
HG329875	629 Jolstra	Ia	PRV1	Piscine reovirus partial mRNA for Sigma 3. segment S1. isolate 629 Jolstra 2008 Wild	Norway	2008	*Salmo salar*	Garseth
HG329876	708 Nausta	Ia	PRV1	Piscine reovirus partial mRNA for Sigma 3. segment S1. isolate 708 Nausta 2008 Wild	Norway	2008	*Salmo salar*	Garseth
HG329877	818 Surna	Ib	PRV1	Piscine reovirus partial mRNA for Sigma 3. segment S1. isolate 818 Surna 2008 Wild	Norway	2008	*Salmo salar*	Garseth
HG329878	842 Vikja	Ia	PRV1	Piscine reovirus partial mRNA for Sigma 3. segment S1. isolate 842 Vikja 2008 Wild	Norway	2008	*Salmo salar*	Garseth
HG329879	851 Vikja	Ia	PRV1	Piscine reovirus partial mRNA for Sigma 3. segment S1. isolate 851 Vikja 2008 Wild	Norway	2008	*Salmo salar*	Garseth
HG329880	866 Vikja	Ia	PRV1	Piscine reovirus partial mRNA for Sigma 3. segment S1. isolate 866 Vikja 2008 Wild	Norway	2008	*Salmo salar*	Garseth
HG329881	907 Vosso	Ia	PRV1	Piscine reovirus partial mRNA for Sigma 3. segment S1. isolate 907 Vosso 2008 Hatchery-reared	Norway	2008	*Salmo salar*	Garseth
HG329882	909 Vosso	Ia	PRV1	Piscine reovirus partial mRNA for Sigma 3. segment S1. isolate 909 Vosso 2008 Hatchery-reared	Norway	2008	*Salmo salar*	Garseth
HG329883	931 Alta	Ia	PRV1	Piscine reovirus partial mRNA for Sigma 3. segment S1. isolate 931 Alta 2007 Wild	Norway	2007	*Salmo salar*	Garseth
HG329884	982 Eira	Ib	PRV1	Piscine reovirus partial mRNA for Sigma 3. segment S1. isolate 982 Eira 2007 Wild	Norway	2007	*Salmo salar*	Garseth
HG329885	985 Ekso	Ia	PRV1	Piscine reovirus partial mRNA for Sigma 3. segment S1. isolate 985 Ekso 2007 wild	Norway	2007	*Salmo salar*	Garseth
HG329886	987 Ekso	Ia	PRV1	Piscine reovirus partial mRNA for Sigma 3. segment S1. isolate 987 Ekso 2007 wild	Norway	2007	*Salmo salar*	Garseth
HG329887	989 Ekso	Ia	PRV1	Piscine reovirus partial mRNA for Sigma 3. segment S1. isolate 989 Ekso 2007 Wild	Norway	2007	*Salmo salar*	Garseth
HG329888	993 Ekso	Ia	PRV1	Piscine reovirus partial mRNA for Sigma 3. segment S1. isolate 993 Ekso 2007 Wild	Norway	2007	*Salmo salar*	Garseth
HG329889	1039 Laerdal	Ia	PRV1	Piscine reovirus partial mRNA for Sigma 3. segment S1. isolate 1039 Laerdal 2007 Wild	Norway	2007	*Salmo salar*	Garseth
HG329890	1062 Mandal	Ia	PRV1	Piscine reovirus partial mRNA for Sigma 3. segment S1. isolate 1062 Mandal 2007 wild	Norway	2007	*Salmo salar*	Garseth
HG329891	1137 Stjordal	Ia	PRV1	Piscine reovirus partial mRNA for Sigma 3. segment S1. isolate 1137 Stjordal 2007 Wild	Norway	2007	*Salmo salar*	Garseth
HG329892	1160 Surna	Ib	PRV1	Piscine reovirus partial mRNA for Sigma 3. segment S1. isolate 1160 Surna 2007 Wild	Norway	2007	*Salmo salar*	Garseth
HG329893	1195 Aaroy	Ia	PRV1	Piscine reovirus partial mRNA for Sigma 3. segment S1. isolate 1195 Aaroy 2007 Wild	Norway	2007	*Salmo salar*	Garseth
HG329894	1261 Halsan	Ib	PRV1	Piscine reovirus partial mRNA for Sigma 3. segment S1. isolate 1261 Halsan 2009 Escaped farmed	Norway	2009	*Salmo salar*	Garseth
HG329895	1309 Eidsdal	Ib	PRV1	Piscine reovirus partial mRNA for Sigma 3. segment S1. isolate 1309 Eidsdal 2008 Wild	Norway	2008	*Salmo salar*	Garseth
HG329896	1343 Moelv	Ia	PRV1	Piscine reovirus partial mRNA for Sigma 3. segment S1. isolate 1343 Moelv 2008 Wild	Norway	2008	*Salmo salar*	Garseth
HG329897	1361 Moelv	Ib	PRV1	Piscine reovirus partial mRNA for Sigma 3. segment S1. isolate 1361 Moelv 2008 Sea-trout	Norway	2008	*Salmo salar*	Garseth
HG329898	1459 Etne	Ib	PRV1	Piscine reovirus partial mRNA for Sigma 3. segment S1. isolate 1459 Etne 2010 Escaped farmed	Norway	2010	*Salmo salar*	Garseth
HG329899	1462 Etne	Ib	PRV1	Piscine reovirus partial mRNA for Sigma 3. segment S1. isolate 1462 Etne 2010 Escaped farmed	Norway	2010	*Salmo salar*	Garseth
HG329900	1463 Etne	Ib	PRV1	Piscine reovirus partial mRNA for Sigma 3. segment S1. isolate 1463 Etne 2010 Escaped farmed	Norway	2010	*Salmo salar*	Garseth
HG329901	1469 Etne	Ib	PRV1	Piscine reovirus partial mRNA for Sigma 3. segment S1. isolate 1469 Etne 2010 Escaped farmed	Norway	2010	*Salmo salar*	Garseth
JN991006	5433-S3	Ia	PRV1	Piscine reovirus isolate 5433 sigma 3 protein gene. partial cds	Norway	2010	*Salmo salar*	Lovoll
JN991007	1921-S3	Ia	PRV1	Piscine reovirus isolate 1921 sigma 3 protein gene. partial cds	Norway	2010	*Salmo salar*	Lovoll
JN991008	9326-S3	Ia	PRV1	Piscine reovirus isolate 9326 sigma 3 protein gene. partial cds	Norway	2010	*Salmo salar*	Lovoll
JN991009	7243-S3	Ib	PRV1	Piscine reovirus isolate 7243 sigma 3 protein gene. partial cds	Norway	2010	*Salmo salar*	Lovoll
JN991010	7030-S3	Ib	PRV1	Piscine reovirus isolate 7030 sigma 3 protein gene. partial cds	Norway	2010	*Salmo salar*	Lovoll
JN991011	8286-S3	Ib	PRV1	Piscine reovirus isolate 8286 sigma 3 protein gene. partial cds	Norway	2010	*Salmo salar*	Lovoll
JN991012	3817-S3	Ia	PRV1	Piscine reovirus isolate 3817 sigma 3 protein gene. partial cds	Norway	2010	*Salmo salar*	Lovoll
JX046542	PRV-M2-R11/11	unknown	unknown	Piscine reovirus isolate PRV-M2-R11/11 outer capsid protein [M2] gene, partial cds	Norway	2011	*Salmo salar*	Tolaas
JX046543	PRV-M2-R12/11	unknown	unknown	Piscine reovirus isolate PRV-M2-R12/11 outer capsid protein [M2] gene, partial cds	Norway	2011	*Salmo salar*	Tolaas
KC473452	VT02292012-163	Ia	PRV1	Piscine reovirus isolate VT02292012-163 sigma 1 and sigma 1S genes. complete cds	Canada	2012	*Salmo salar*	Kibenge
KC473453	VT06062012-358	Ia	PRV1	Piscine reovirus isolate VT06062012-358 sigma 1 and sigma 1S genes. complete cds	Canada	2012	*Salmo salar*	Kibenge
KC473454	VT06202012-371	Ia	PRV1	Piscine reovirus isolate VT06202012-371 sigma 1 and sigma 1S genes. complete cds	Canada	2012	*Salmo salar*	Kibenge
KC715683	VT06062012-358	Ia	PRV1	Piscine reovirus isolate VT06062012-358 outer shell protein mRNA, complete cds	Canada	2012	*Salmo salar*	Kibenge
KC776260	VT06202012-371	Ia	PRV1	Piscine reovirus isolate VT06202012-371 outer shell protein mRNA, complete cds	Canada	2012	*Salmo salar*	Kibenge
KC782501	CGA337	Ib	PRV1	UNVERIFIED: Piscine reovirus isolate CGA/337/2012 sigma 3-like gene. partial sequence	Chile	2012	*Salmo salar*	Kibenge
KC790988	CGA8857	Ib	PRV1	UNVERIFIED: Piscine reovirus isolate CGA/8857/2012 sigma 3-like gene. partial sequence	Chile	2012	*Salmo salar*	Kibenge
KC795569	CGA280-05	Ib	PRV1	Piscine reovirus isolate CGA280-05 outer shell protein mRNA, complete cds	Chile	2012	*Salmo salar*	Kibenge
KC795571	CGA280-05	Ib	PRV1	Piscine reovirus isolate CGA280-05 outer clamp protein and non-structural protein genes. complete cds	Chile	2012	*Salmo salar*	Kibenge
KC795593	VT02292012-163	Ia	PRV1	Piscine reovirus isolate VT02292012-163 outer shell protein mRNA, partial cds	Canada	2012	*Salmo salar*	Kibenge
KC795594	VT02292012-167	Ia	PRV1	Piscine reovirus isolate VT02292012-167 outer shell protein mRNA, partial cds	Canada	2012	*Salmo salar*	Kibenge
KC795599	VT02292012-167	Ia	PRV1	Piscine reovirus isolate VT02292012-167 outer clamp protein and non-structural protein genes. complete	Canada	2012	*Salmo salar*	Kibenge
KC795600	VT03202012-196	Ia	PRV1	Piscine reovirus isolate VT03202012-196 outer clamp protein and non-structural protein genes. complete	Canada	2012	*Salmo salar*	Kibenge
KC795601	VT03202012-209	Ia	PRV1	Piscine reovirus isolate VT03202012-209 outer clamp protein and non-structural protein genes. complete	Canada	2012	*Salmo salar*	Kibenge
KR337477	50607	Ib	PRV1	Piscine reovirus isolate 050607 segment M2 mu 1 protein gene, complete cds	Norway	2007	*Salmo salar*	Haatveit
KR337479	50607	Ib	PRV1	Piscine reovirus isolate 050607 segment S1 sigma 3 protein and p13 protein genes. complete cds	Norway	2007	*Salmo salar*	Haatveit
KR347078	BCA1849_01	Ia	PRV1	Piscine reovirus isolate BCA1849_01 sigma 3 gene. partial cds; and protein p13 gene. complete cds	Canada	2001	*Salmo salar*	Siah
KR347079	BCA1850_01	Ia	PRV1	Piscine reovirus isolate BCA1850_01 sigma 3 gene. partial cds; and protein p13 gene. complete cds	Canada	2001	*Salmo salar*	Siah
KR347080	BCA1854_05	Ia	PRV1	Piscine reovirus isolate BCA1854_05 sigma 3 gene. partial cds; and protein p13 gene. complete cds	Canada	2005	*Salmo salar*	Siah
KR347081	BCJ18824_13	Ia	PRV1	Piscine reovirus isolate BCJ18824_13 sigma 3 gene. partial cds; and protein p13 gene. complete cds	Canada	2013	*Oncorhynchus tshawytscha*	Siah
KR347082	BCJ19323_13	Ia	PRV1	Piscine reovirus isolate BCJ19323_13 sigma 3 gene. partial cds; and protein p13 gene. complete cds	Canada	2013	*Oncorhynchus kisutch*	Siah
KR347083	BCJ19943_13	Ia	PRV1	Piscine reovirus isolate BCJ19943_13 sigma 3 gene. partial cds; and protein p13 gene. complete cds	Canada	2013	*Oncorhynchus kisutch*	Siah
KR347084	BCJ24201_13	Ia	PRV1	Piscine reovirus isolate BCJ24201_13 sigma 3 gene. partial cds; and protein p13 gene. complete cds	Canada	2013	*Salmo salar*	Siah
KR347085	BCJ28529_13	Ia	PRV1	Piscine reovirus isolate BCJ28529_13 sigma 3 gene. partial cds; and protein p13 gene. complete cds	Canada	2013	*Salmo salar*	Siah
KR347086	BCJ28537_13	Ia	PRV1	Piscine reovirus isolate BCJ28537_13 sigma 3 gene. partial cds; and protein p13 gene. complete cds	Canada	2013	*Salmo salar*	Siah
KR347087	BCJ28545_13	Ia	PRV1	Piscine reovirus isolate BCJ28545_13 sigma 3 gene. partial cds; and protein p13 gene. complete cds	Canada	2013	*Salmo salar*	Siah
KR347088	BCJ31910_13	Ia	PRV1	Piscine reovirus isolate BCJ31910_13 sigma 3 gene. partial cds; and protein p13 gene. complete cds	Canada	2013	*Salmo salar*	Siah
KR347089	BCJ31914_13	Ia	PRV1	Piscine reovirus isolate BCJ31914_13 sigma 3 gene. partial cds; and protein p13 gene. complete cds	Canada	2013	*Salmo salar*	Siah
KR347090	BCJ31915_13	Ia	PRV1	Piscine reovirus isolate BCJ31915_13 sigma 3 gene. partial cds; and protein p13 gene. complete cds	Canada	2013	*Salmo salar*	Siah
KR347091	BCJ31916_13	Ia	PRV1	Piscine reovirus isolate BCJ31916_13 sigma 3 gene. partial cds; and protein p13 gene. complete cds	Canada	2013	*Salmo salar*	Siah
KR347092	BCJ31920_13	Ia	PRV1	Piscine reovirus isolate BCJ31920_13 sigma 3 gene. partial cds; and protein p13 gene. complete cds	Canada	2013	*Salmo salar*	Siah
KR347093	BCJ34056_13	Ia	PRV1	Piscine reovirus isolate BCJ34056_13 sigma 3 gene. partial cds; and protein p13 gene. complete cds	Canada	2013	*Oncorhynchus kisutch*	Siah
KR347094	BCJ35240_13	Ia	PRV1	Piscine reovirus isolate BCJ35240_13 sigma 3 gene. partial cds; and protein p13 gene. complete cds	Canada	2013	*Salmo salar*	Siah
KR347095	BCJ35246_13	Ia	PRV1	Piscine reovirus isolate BCJ35246_13 sigma 3 gene. partial cds; and protein p13 gene. complete cds	Canada	2013	*Salmo salar*	Siah
KR347096	BCJ35249_13	Ia	PRV1	Piscine reovirus isolate BCJ35249_13 sigma 3 gene. partial cds; and protein p13 gene. complete cds	Canada	2013	*Salmo salar*	Siah
KR347097	BCJ35255_13	Ia	PRV1	Piscine reovirus isolate BCJ35255_13 sigma 3 gene. partial cds; and protein p13 gene. complete cds	Canada	2013	*Salmo salar*	Siah
KR347098	BCJ35256_13	Ia	PRV1	Piscine reovirus isolate BCJ35256_13 sigma 3 gene. partial cds; and protein p13 gene. complete cds	Canada	2013	*Salmo salar*	Siah
KR347099	BCJ37896_13	Ia	PRV1	Piscine reovirus isolate BCJ37896_13 sigma 3 gene. partial cds; and protein p13 gene. complete cds	Canada	2013	*Oncorhynchus kisutch*	Siah
KR347100	BCJ40723_13	Ia	PRV1	Piscine reovirus isolate BCJ40723_13 sigma 3 gene. partial cds; and protein p13 gene. complete cds	Canada	2013	*Salmo salar*	Siah
KR347101	BCJ40731_13	Ia	PRV1	Piscine reovirus isolate BCJ40731_13 sigma 3 gene. partial cds; and protein p13 gene. complete cds	Canada	2013	*Salmo salar*	Siah
KR347102	BCJ40740_13	Ia	PRV1	Piscine reovirus isolate BCJ40740_13 sigma 3 gene. partial cds; and protein p13 gene. complete cds	Canada	2013	*Salmo salar*	Siah
KR347103	BCJ378151_13	Ia	PRV1	Piscine reovirus isolate BCJ378151_13 sigma 3 gene. partial cds; and protein p13 gene. complete cds	Canada	2013	*Oncorhynchus kisutch*	Siah
KR347104	BCJ378241_13	Ia	PRV1	Piscine reovirus isolate BCJ378241_13 sigma 3 gene. partial cds; and protein p13 gene. complete cds	Canada	2013	*Oncorhynchus kisutch*	Siah
KR347105	BCJ402256_13	Ia	PRV1	Piscine reovirus isolate BCJ402256_13 sigma 3 gene. partial cds; and protein p13 gene. complete cds	Canada	2013	*Oncorhynchus kisutch*	Siah
KR347106	BCJ402276_13	Ia	PRV1	Piscine reovirus isolate BCJ402276_13 sigma 3 gene. partial cds; and protein p13 gene. complete cds	Canada	2013	*Oncorhynchus kisutch*	Siah
KR347107	BCJ402334_13	Ia	PRV1	Piscine reovirus isolate BCJ402334_13 sigma 3 gene. partial cds; and protein p13 gene. complete cds	Canada	2013	*Oncorhynchus kisutch*	Siah
KR347108	BCK1435_14	Ia	PRV1	Piscine reovirus isolate BCK1435_14 sigma 3 gene. partial cds; and protein p13 gene. complete cds	Canada	2014	*Salmo salar*	Siah
KR347109	BCK1436_14	Ia	PRV1	Piscine reovirus isolate BCK1436_14 sigma 3 gene. partial cds; and protein p13 gene. complete cds	Canada	2014	*Salmo salar*	Siah
KR347110	BCK1562_14	Ia	PRV1	Piscine reovirus isolate BCK1562_14 sigma 3 gene. partial cds; and protein p13 gene. complete cds	Canada	2014	*Oncorhynchus kisutch*	Siah
KR347111	BCK1566_14	Ia	PRV1	Piscine reovirus isolate BCK1566_14 sigma 3 gene. partial cds; and protein p13 gene. complete cds	Canada	2014	*Oncorhynchus kisutch*	Siah
KR347112	BCK14114_14	Ia	PRV1	Piscine reovirus isolate BCK14114_14 sigma 3 gene. partial cds; and protein p13 gene. complete cds	Canada	2014	*Salmo salar*	Siah
KR347113	BCK14120_14	Ia	PRV1	Piscine reovirus isolate BCK14120_14 sigma 3 gene. partial cds; and protein p13 gene. complete cds	Canada	2014	*Salmo salar*	Siah
KR347114	BCK14310_14	Ia	PRV1	Piscine reovirus isolate BCK14310_14 sigma 3 gene. partial cds; and protein p13 gene. complete cds	Canada	2014	*Salmo salar*	Siah
KR347115	BCK15625_14	Ia	PRV1	Piscine reovirus isolate BCK15625_14 sigma 3 gene. partial cds; and protein p13 gene. complete cds	Canada	2014	*Oncorhynchus kisutch*	Siah
KR478633	WS1207_12	Ia	PRV1	Piscine reovirus isolate WS1207_12 sigma 3 gene. partial cds; and p13 protein gene. complete cds	USA	2012	*Oncorhynchus tshawytscha*	Siah
KR478634	WS1209_12	Ia	PRV1	Piscine reovirus isolate WS1209_12 sigma 3 gene. partial cds; and p13 protein gene. complete cds	USA	2012	*Oncorhynchus tshawytscha*	Siah
KR478635	WSKFH1_14	Ia	PRV1	Piscine reovirus isolate WSKFH1_14 sigma 3 gene. partial cds; and p13 protein gene. complete cds	USA	2014	*Oncorhynchus kisutch*	Siah
KR478636	WSKFH2_14	Ia	PRV1	Piscine reovirus isolate WSKFH2_14 sigma 3 gene. partial cds; and p13 protein gene. complete cds	USA	2014	*Oncorhynchus kisutch*	Siah
KR478637	WSKFH11_14	Ia	PRV1	Piscine reovirus isolate WSKFH11_14 sigma 3 gene. partial cds; and p13 protein gene. complete cds	USA	2014	*Oncorhynchus kisutch*	Siah
KR478638	WSKFH12_14	Ia	PRV1	Piscine reovirus isolate WSKFH12_14 sigma 3 gene. partial cds; and p13 protein gene. complete cds	USA	2014	*Oncorhynchus kisutch*	Siah
KR478639	WSKFH13_14	Ia	PRV1	Piscine reovirus isolate WSKFH13_14 sigma 3 gene. partial cds; and p13 protein gene. complete cds	USA	2014	*Oncorhynchus kisutch*	Siah
KR478640	AKJ20115_13	Ia	PRV1	Piscine reovirus isolate AKJ20115_13 sigma 3 gene. partial cds; and sigma 3 gene. complete cds	USA	2013	*Oncorhynchus kisutch*	Siah
KR478641	AKJ20120_13	Ia	PRV1	Piscine reovirus isolate AKJ20120_13 sigma 3 gene. partial cds; and p13 protein gene. complete cds	USA	2013	*Oncorhynchus kisutch*	Siah
KR478642	BCA1338_01	Ia	PRV1	Piscine reovirus isolate BCA1338_01 sigma 3 gene. partial cds; and p13 protein gene. complete cds	Canada	2001	*Oncorhynchus tshawytscha*	Siah
KR478643	BCA1846_01	Ia	PRV1	Piscine reovirus isolate BCA1846_01 sigma 3 gene. partial cds; and p13 protein gene. complete cds	Canada	2001	*Salmo salar*	Siah
KR478644	BCA1848_01	Ia	PRV1	Piscine reovirus isolate BCA1848_01 sigma 3 gene. partial cds; and p13 protein gene. complete cds	Canada	2001	*Salmo salar*	Siah
KR558677	BC131_13	Ia	PRV1	Piscine reovirus isolate BC131_13 sigma 3 gene. partial cds	Canada	2013	*Salmo salar*	Siah
KR558678	BC132_13	Ia	PRV1	Piscine reovirus isolate BC132_13 sigma 3 gene. partial cds	Canada	2013	*Salmo salar*	Siah
KR558679	BC133_13	Ia	PRV1	Piscine reovirus isolate BC133_13 sigma 3 gene. partial cds	Canada	2013	*Salmo salar*	Siah
KR558680	BC134_13	Ia	PRV1	Piscine reovirus isolate BC134_13 sigma 3 gene. partial cds	Canada	2013	*Salmo salar*	Siah
KR558681	BC135_13	Ia	PRV1	Piscine reovirus isolate BC135_13 sigma 3 gene. partial cds	Canada	2013	*Salmo salar*	Siah
KR558682	BC136_13	Ia	PRV1	Piscine reovirus isolate BC136_13 sigma 3 gene. partial cds	Canada	2013	*Salmo salar*	Siah
KR558683	BC137_13	Ia	PRV1	Piscine reovirus isolate BC137_13 sigma 3 gene. partial cds	Canada	2013	*Salmo salar*	Siah
KR558684	BC138_13	Ia	PRV1	Piscine reovirus isolate BC138_13 sigma 3 gene. partial cds	Canada	2013	*Salmo salar*	Siah
KR558685	BC139_13	Ia	PRV1	Piscine reovirus isolate BC139_13 sigma 3 gene. partial cds	Canada	2013	*Salmo salar*	Siah
KR781117	BC1310_13	Ia	PRV1	Piscine reovirus isolate BC1310_13 sigma 3 gene. partial cds	Canada	2013	*Salmo salar*	Siah
KR781118	BC1311_13	Ia	PRV1	Piscine reovirus isolate BC1311_13 sigma 3 gene. partial cds	Canada	2013	*Salmo salar*	Siah
KR872635	BCinoc3	Ia	PRV1	Piscine reovirus isolate BCinoc3 segment S1 sigma 3 gene. partial cds	Canada	2012	*Oncorhynchus tshawytscha*	Garver
KR872636	BCinoc12	Ia	PRV1	Piscine reovirus isolate BCinoc12 segment S1 sigma 3 gene. partial cds	Canada	2012	*Oncorhynchus tshawytscha*	Garver
KR872637	BC361_14	Ia	PRV1	Piscine reovirus isolate BC361_14 segment S1 sigma 3 gene. partial cds	Canada	2014	*Salmo salar*	Garver
KT429734	BCJ19943_13	Ia	PRV1	Piscine reovirus isolate BCJ19943_13 outer shell protein mRNA, complete cds	Canada	2013	*Oncorhynchus kisutch*	Siah
KT429736	BCJ19943_13	Ia	PRV1	Piscine reovirus isolate BCJ19943_13 sigma 1 and sigma 1S mRNAs. complete cds	Canada	2013	*Oncorhynchus kisutch*	Siah
KT429744	BCJ31915_13	Ia	PRV1	Piscine reovirus isolate BCJ31915_13 outer shell protein mRNA, partial cds	Canada	2013	*Salmo salar*	Siah
KT429746	BCJ31915_13	Ia	PRV1	Piscine reovirus isolate BCJ31915_13 sigma 1 and sigma 1S mRNAs. complete cds	Canada	2013	*Salmo salar*	Siah
KT429754	WSKFH12_14	Ia	PRV1	Piscine reovirus isolate WSKFH12_14 outer shell protein mRNA, complete cds	USA	2014	*Oncorhynchus kisutch*	Siah
KT429756	WSKFH12_14	Ia	PRV1	Piscine reovirus isolate WSKFH12_14 sigma 1 and sigma 1S mRNAs. complete cds	USA	2014	*Oncorhynchus kisutch*	Siah
KT456500	VT09122012-755	Ia	PRV1	Piscine reovirus isolate VT09122012-755 segment S1 outer clamp protein and p13 protein genes. complete	Canada	2011	*Oncorhynchus tshawytscha*	Kibenge
KT456501	VT09192013-402	Ia	PRV1	Piscine reovirus isolate VT09192013-402 segment S1 outer clamp protein and p13 protein genes. complete	Canada	2013	*Oncorhynchus kisutch*	Kibenge
KT456502	VT09192013-408	Ia	PRV1	Piscine reovirus isolate VT09192013-408 segment S1 outer clamp protein and p13 protein genes. complete	Canada	2013	*Oncorhynchus kisutch*	Kibenge
KT456503	VT01212014-03	Ia	PRV1	Piscine reovirus isolate VT01212014-03 segment S1 outer clamp protein and p13 protein genes. complete	Canada	2014	*Salmo salar*	Kibenge
KT456504	VT01212014-04	Ia	PRV1	Piscine reovirus isolate VT01212014-04 segment S1 outer clamp protein and p13 protein genes. complete	Canada	2014	*Salmo salar*	Kibenge
KT456505	VT01212014-09	Ia	PRV1	Piscine reovirus isolate VT01212014-09 segment S1 outer clamp protein and p13 protein genes. complete	Iceland	2014	*Salmo salar*	Kibenge
KU131591	VT12202013_CGA_2013_4	Ia	PRV1	Piscine reovirus isolate VT12202013-CGA-2013-4 segment S1 outer clamp protein and p13 protein genes	Chile	2013	*Oncorhynchus kisutch*	Godoy
KU131592	VT12202013_CGA_2013_1	Ia	PRV1	Piscine reovirus isolate VT12202013-CGA-2013-1 segment S1 outer clamp protein and p13 protein genes	Chile	2013	*Oncorhynchus kisutch*	Godoy
KU131593	VT12202013_CGA_2013_2	Ia	PRV1	Piscine reovirus isolate VT12202013-CGA-2013-2 segment S1 outer clamp protein and p13 protein genes	Chile	2013	*Oncorhynchus kisutch*	Godoy
KU131594	2015_CGA_2015_B	Ia	PRV1	Piscine reovirus isolate 2015-CGA-2015-B segment S1 outer clamp protein gene. partial cds; and p13	Chile	2015	*Oncorhynchus kisutch*	Godoy
KU131595	VT12202013_CGA_2013_3	IIa	PRV3	Piscine reovirus isolate VT12202013-CGA-2013-3 segment S1 outer clamp protein and p13 protein genes	Chile	2013	*Oncorhynchus kisutch*	Godoy
KU131596	VT12202013_CGA_2013_5	IIa	PRV3	Piscine reovirus isolate VT12202013-CGA-2013-5 segment S1 outer clamp protein and p13 protein genes	Chile	2013	*Oncorhynchus kisutch*	Godoy
KU131597	2013_CGA_2013_A	Ib	PRV1	Piscine reovirus isolate 2013-CGA-2013-A segment S1 outer clamp protein and p13 protein genes	Chile	2013	*Salmo salar*	Godoy
KU131598	2013_CGA_2013_C	Ib	PRV1	Piscine reovirus isolate 2013-CGA-2013-C segment S1 outer clamp protein and p13 protein genes	Chile	2013	*Oncorhynchus kisutch*	Godoy
KU131599	VT02182014_CGA_2013_8	Ib	PRV1	Piscine reovirus isolate VT02182014-CGA-2013-8 segment S1 outer clamp protein and p13 protein genes	Chile	2013	*Salmo salar*	Godoy
KU131600	VT02182014_CGA_2013_9	Ib	PRV1	Piscine reovirus isolate VT02182014-CGA-2013-9 segment S1 outer clamp protein and p13 protein genes	Chile	2013	*Salmo salar*	Godoy
KU131601	VT02182014_CGA_2013_10	Ib	PRV1	Piscine reovirus isolate VT02182014-CGA-2013-10 segment S1 outer clamp protein and p13 protein genes	Chile	2013	*Salmo salar*	Godoy
KU131602	VT02182014_CGA_2013_6	Ib	PRV1	Piscine reovirus isolate VT02182014-CGA-2013-6 segment S1 outer clamp protein and p13 protein genes	Chile	2013	*Salmo salar*	Godoy
KU131603	VT02182014_CGA_2013_7	Ib	PRV1	Piscine reovirus isolate VT02182014-CGA-2013-7 segment S1 outer clamp protein and p13 protein genes	Chile	2013	*Salmo salar*	Godoy
KU131604	2015_CGA_2015_A	Ib	PRV1	Piscine reovirus isolate 2015-CGA-2015-A segment S1 outer clamp protein and p13 protein genes	Chile	2015	*Oncorhynchus kisutch*	Godoy
KU131605	2015_CGA_2015_C	Ib	PRV1	Piscine reovirus isolate 2015-CGA-2015-C segment S1 outer clamp protein gene. partial cds; and p13	Chile	2015	*Salmo salar*	Godoy
KU160513	VT01292015-09	Ia	PRV1	Piscine reovirus isolate VT01292015-09 segment S1 outer clamp protein and p13 protein genes. complete	Canada	2015	*Salmo salar*	Kibenge
KU160514	VT07222015-106	Ia	PRV1	Piscine reovirus isolate VT07222015-106 segment S1 outer clamp protein and p13 protein genes. complete	Canada	2015	*Salmo salar*	Kibenge

KU160515	VT07222015-107	Ia	PRV1	Piscine reovirus isolate VT07222015-107 segment S1 outer clamp protein and p13 protein genes. complete	Canada	2015	*Salmo salar*	Kibenge
KX844951	C10/P4.1	IIa	PRV3	Piscine reovirus isolate C10/P4.1 sigma 3 protein (OCPSigma3) gene. complete cds	Chile	2014	*Oncorhynchus mykiss*	Cartagena
KX844952	IM/P5.2	Ib	PRV1	Piscine reovirus isolate IM/P5.2 sigma 3 protein (OCPSigma3) gene. complete cds	Chile	2015	*Oncorhynchus mykiss*	Cartagena
KX844953	V/P3.2	Ia	PRV1	Piscine reovirus isolate V/P3.2 sigma 3 protein (OCPSigma3) gene. complete cds	Chile	2014	*Oncorhynchus mykiss*	Cartagena
KX844954	V/P3.1	Ia	PRV1	Piscine reovirus isolate V/P3.1 sigma 3 protein (OCPSigma3) gene. complete cds	Chile	2014	*Oncorhynchus mykiss*	Cartagena
KX844955	V/P2.2	Ia	PRV1	Piscine reovirus isolate V/P2.2 sigma 3 protein (OCPSigma3) gene. complete cds	Chile	2014	*Oncorhynchus mykiss*	Cartagena
KX844956	V/P2.1	Ia	PRV1	Piscine reovirus isolate V/P2.1 sigma 3 protein (OCPSigma3) gene. complete cds	Chile	2014	*Oncorhynchus mykiss*	Cartagena
KX844957	V/P1.2	Ia	PRV1	Piscine reovirus isolate V/P1.2 sigma 3 protein (OCPSigma3) gene. complete cds	Chile	2014	*Oncorhynchus mykiss*	Cartagena
KX844958	V/P1.1	Ia	PRV1	Piscine reovirus isolate V/P1.1 sigma 3 protein (OCPSigma3) gene. complete cds	Chile	2014	*Oncorhynchus mykiss*	Cartagena
KX844959	C10/P4.2	IIa	PRV3	Piscine reovirus isolate C10/P4.2 sigma 3 protein (OCPSigma3) gene. complete cds	Chile	2014	*Oncorhynchus mykiss*	Cartagena
KX844960	C10/P3.2	IIa	PRV3	Piscine reovirus isolate C10/P3.2 sigma 3 protein (OCPSigma3) gene. complete cds	Chile	2014	*Oncorhynchus mykiss*	Cartagena
KX844961	C10/P3.1	IIa	PRV3	Piscine reovirus isolate C10/P3.1 sigma 3 protein (OCPSigma3) gene. complete cds	Chile	2014	*Oncorhynchus mykiss*	Cartagena
KX844962	C10/P2.2	IIa	PRV3	Piscine reovirus isolate C10/P2.2 sigma 3 protein (OCPSigma3) gene. complete cds	Chile	2014	*Oncorhynchus mykiss*	Cartagena
KX844963	C10/P2.1	IIa	PRV3	UNVERIFIED: Piscine reovirus isolate C10/P2.1 sigma 3 protein-like (OCPSigma3) gene. complete sequence	Chile	2014	*Oncorhynchus mykiss*	Cartagena
KX844964	C10/P1.2	IIa	PRV3	Piscine reovirus isolate C10/P1.2 sigma 3 protein (OCPSigma3) gene. complete cds	Chile	2014	*Oncorhynchus mykiss*	Cartagena
KX844965	C10/P1.1	IIa	PRV3	Piscine reovirus isolate C10/P1.1 sigma 3 protein (OCPSigma3) gene. complete cds	Chile	2014	*Oncorhynchus mykiss*	Cartagena
KX851970	B5690	Ia	PRV1	dump description	Canada	2013	*Salmo salar*	Di Cicco
KX851971	B7274	Ia	PRV1	dump description	Canada	2013	*Salmo salar*	Di Cicco
KX851974	B7274	Ia	PRV1	Piscine reovirus isolate B7274 segment M2, complete sequence	Canada	2013	*Salmo salar*	Di Cicco
KX851975	B5690	Ia	PRV1	Piscine reovirus isolate B5690 segment M2, complete sequence	Canada	2013	*Salmo salar*	Di Cicco
KY369097	12-07_4	Ia	PRV1	Piscine orthoreovirus isolate 12-07_4 sigma 3 and p13 protein genes. partial cds	USA	2012	*Oncorhynchus tshawytscha*	Purcell
KY369098	12-09_1	Ia	PRV1	Piscine orthoreovirus isolate 12-09_1 sigma 3 and p13 protein genes. partial cds	USA	2012	*Oncorhynchus tshawytscha*	Purcell
KY369099	13-18_12	Ia	PRV1	Piscine orthoreovirus isolate 13-18_12 sigma 3 and p13 protein genes. partial cds	USA	2012	*Oncorhynchus kisutch*	Purcell
KY369100	13-26_10	Ia	PRV1	Piscine orthoreovirus isolate 13-26_10 sigma 3 and p13 protein genes. partial cds	USA	2012	*Oncorhynchus kisutch*	Purcell
KY369101	13-33_1389	Ia	PRV1	Piscine orthoreovirus isolate 13-33_1389 sigma 3 and p13 protein genes. partial cds	USA	2012	*Oncorhynchus kisutch*	Purcell
KY369102	13_33_1395	Ia	PRV1	Piscine orthoreovirus isolate 13_33_1395 sigma 3 and p13 protein genes. partial cds	USA	2012	*Oncorhynchus kisutch*	Purcell
KY369103	13_37_1361	Ia	PRV1	Piscine orthoreovirus isolate 13_37_1361 sigma 3 and p13 protein genes. partial cds	USA	2012	*Oncorhynchus mykiss*	Purcell
KY369104	13-6_1587	Ia	PRV1	Piscine orthoreovirus isolate 13-6_1587 sigma 3 and p13 protein genes. partial cds	USA	2012	*Oncorhynchus tshawytscha*	Purcell
KY369105	13-6_1594	Ia	PRV1	Piscine orthoreovirus isolate 13-6_1594 sigma 3 and p13 protein genes. partial cds	USA	2012	*Oncorhynchus tshawytscha*	Purcell
KY369106	14-11_3	Ia	PRV1	Piscine orthoreovirus isolate 14-11_3 sigma 3 and p13 protein genes. partial cds	USA	2012	*Oncorhynchus tshawytscha*	Purcell
KY369107	14-18_13	Ia	PRV1	Piscine orthoreovirus isolate 14-18_13 sigma 3 and p13 protein genes. partial cds	USA	2012	*Salmo salar*	Purcell
KY369108	14-21_16	Ia	PRV1	Piscine orthoreovirus isolate 14-21_16 sigma 3 and p13 protein genes. partial cds	USA	2012	*Oncorhynchus kisutch*	Purcell
KY369109	14-22_12	Ia	PRV1	Piscine orthoreovirus isolate 14-22_12 sigma 3 and p13 protein genes. partial cds	USA	2012	*Oncorhynchus kisutch*	Purcell
KY369110	14-29_15	Ia	PRV1	Piscine orthoreovirus isolate 14-29_15 sigma 3 and p13 protein genes. partial cds	USA	2012	*Oncorhynchus kisutch*	Purcell
KY369111	14-29_2	Ia	PRV1	Piscine orthoreovirus isolate 14-29_2 sigma 3 and p13 protein genes. partial cds	USA	2012	*Oncorhynchus kisutch*	Purcell
KY369112	14-30_5	Ia	PRV1	Piscine orthoreovirus isolate 14-30_5 sigma 3 and p13 protein genes. partial cds	USA	2013	*Oncorhynchus tshawytscha*	Purcell
KY369113	15-10_8	Ia	PRV1	Piscine orthoreovirus isolate 15-10_8 sigma 3 and p13 protein genes. partial cds	USA	2013	*Oncorhynchus kisutch*	Purcell
KY369114	15-3_9	Ia	PRV1	Piscine orthoreovirus isolate 15-3_9 sigma 3 and p13 protein genes. partial cds	USA	2013	*Oncorhynchus tshawytscha*	Purcell
KY369115	15-8_2	Ia	PRV1	Piscine orthoreovirus isolate 15-8_2 sigma 3 and p13 protein genes. partial cds	USA	2013	*Oncorhynchus kisutch*	Purcell
KY369116	15-9_5	Ia	PRV1	Piscine orthoreovirus isolate 15-9_5 sigma 3 and p13 protein genes. partial cds	USA	2013	*Oncorhynchus kisutch*	Purcell
KY369117	15-28_9	Ia	PRV1	Piscine orthoreovirus isolate 15-28_9 sigma 3 and p13 protein genes. partial cds	USA	2013	*Oncorhynchus gorbuscha*	Purcell
KY369118	15-29_5	Ia	PRV1	Piscine orthoreovirus isolate 15-29_5 sigma 3 and p13 protein genes. partial cds	USA	2013	*Oncorhynchus kisutch*	Purcell
KY369119	15-29_8	Ia	PRV1	Piscine orthoreovirus isolate 15-29_8 sigma 3 and p13 protein genes. partial cds	USA	2013	*Oncorhynchus kisutch*	Purcell
KY369120	15-30_15	Ia	PRV1	Piscine orthoreovirus isolate 15-30_15 sigma 3 and p13 protein genes. partial cds	USA	2013	*Oncorhynchus kisutch*	Purcell
KY369121	15-56_17	Ia	PRV1	Piscine orthoreovirus isolate 15-56_17 sigma 3 and p13 protein genes. partial cds	USA	2013	*Oncorhynchus kisutch*	Purcell
KY369122	15-57_1	Ia	PRV1	Piscine orthoreovirus isolate 15-57_1 sigma 3 and p13 protein genes. partial cds	USA	2013	*Oncorhynchus kisutch*	Purcell
KY369123	15-57_8	Ia	PRV1	Piscine orthoreovirus isolate 15-57_8 sigma 3 and p13 protein genes. partial cds	USA	2013	*Oncorhynchus kisutch*	Purcell
KY369124	15-32_3	Ia	PRV1	Piscine orthoreovirus isolate 15-32_3 p13 protein and sigma 3 genes. partial cds	USA	2013	*Oncorhynchus kisutch*	Purcell
KY369125	15-21_14	Ia	PRV1	Piscine orthoreovirus isolate 15-21_14 sigma 3 and p13 protein genes. partial cds	USA	2013	*Oncorhynchus tshawytscha*	Purcell
KY369126	15-26_1	Ia	PRV1	Piscine orthoreovirus isolate 15-26_1 sigma 3 and p13 protein genes. partial cds	USA	2013	*Oncorhynchus kisutch*	Purcell
KY379959	15-34_11	Ia	PRV1	Piscine orthoreovirus isolate 15-34_11 sigma 3 and p13 protein genes. partial cds	USA	2013	*Oncorhynchus kisutch*	Purcell
KY429947	NOR2012-V3621	Ib	PRV1	Piscine orthoreovirus isolate NOR2012-V3621 segment M2, complete sequence	Norway	2012	*Salmo salar*	Wessel
KY429949	NOR2012-V3621	Ib	PRV1	dump description	Norway	2012	*Salmo salar*	Wessel
LC145613	PRV-2	IIb	PRV2	Piscine orthoreovirus 2 genomic RNA, segment M2, complete sequence	Japan	2012	*Oncorhynchus kisutch*	Takano
LC145616	PRV-2	IIb	PRV2	Piscine orthoreovirus 2 genomic RNA, segment S1, complete sequence	Japan	2012	*Oncorhynchus kisutch*	Takano
LN680851	F445-2013/NOR	IIa	PRV3	Reovirus sp. rainbow trout/F445-2013/NOR partial segment 1 for sigma 3 protein	Norway	2013	*Oncorhynchus mykiss*	Olsen
MF946290	VT10092015-122	Ia	PRV1	Piscine orthoreovirus isolate VT10092015-122 outer clamp protein and p13 genes. complete cds	Canada	2015	*Salmo salar*	Kibenge
MF946291	VT10092015-123	Ia	PRV1	Piscine orthoreovirus isolate VT10092015-123 outer clamp protein and p13 genes. complete cds	Canada	2015	*Salmo salar*	Kibenge
MF946292	VT10092015-125	Ia	PRV1	Piscine orthoreovirus isolate VT10092015-125 outer clamp protein and p13 genes. complete cds	Canada	2015	*Salmo salar*	Kibenge
MF946293	VT12072015-130	Ia	PRV1	Piscine orthoreovirus isolate VT12072015-130 outer clamp protein and p13 genes. complete cds	Canada	2015	*Salmo salar*	Kibenge
MF946294	VT12072015-132	Ia	PRV1	Piscine orthoreovirus isolate VT12072015-132 outer clamp protein and p13 genes. complete cds	Canada	2015	*Salmo salar*	Kibenge
MF946295	VT12072015-137	Ia	PRV1	Piscine orthoreovirus isolate VT12072015-137 outer clamp protein and p13 genes. complete cds	Canada	2015	*Salmo salar*	Kibenge
MF946296	VT12162015-141	Ia	PRV1	Piscine orthoreovirus isolate VT12162015-141 outer clamp protein and p13 genes. complete cds	Canada	2015	*Salmo salar*	Kibenge
MF946297	VT01222016-15	Ia	PRV1	Piscine orthoreovirus isolate VT01222016-15 outer clamp protein and p13 genes. complete cds	Canada	2015	*Salmo salar*	Kibenge
MF946298	VT06082016-37	Ia	PRV1	Piscine orthoreovirus isolate VT06082016-37 outer clamp protein and p13 genes. complete cds	Canada	2016	*Salmo salar*	Kibenge
MF946299	VT02162017-59	Ia	PRV1	Piscine orthoreovirus isolate VT02162017-59 outer clamp protein and p13 genes. complete cds	Norway	2017	*Salmo salar*	Kibenge
MF946300	VT03022017-69	Ia	PRV1	Piscine orthoreovirus isolate VT03022017-69 outer clamp protein and p13 genes. complete cds	Canada	2017	*Salmo salar*	Kibenge
MF946301	VT03302017-99	Ia	PRV1	Piscine orthoreovirus isolate VT03302017-99 outer clamp protein and p13 genes. complete cds	Canada	2017	*Salmo salar*	Kibenge
MF946302	VT04282017A-108-10	Ia	PRV1	Piscine orthoreovirus isolate VT04282017A-108-10 outer clamp protein and p13 genes. complete cds	Canada	2017	*Salmo salar*	Kibenge
MG253811	NOR/060214	IIa	PRV3	Piscine orthoreovirus 3 strain NOR/060214 segment M2 mu 1 protein gene, complete cds	Norway	2013	*Onchorynchus mykiss*	Dhamotharan
MG253816	NOR/060214	IIa	PRV3	Piscine orthoreovirus 3 strain NOR/060214 segment S1 sigma 3 and p13 genes. complete cds	Norway	2014	*Oncorhynchus mykiss*	Dhamotharan
MG720339	12-105_(58)	Ib	PRV1	Piscine orthoreovirus isolate 12-105_(58) F-Andoy 2012 Escaped segment S1 sigma 3 protein gene	Norway	2012	*Salmo salar*	Madhun
MG720340	12-112_(59)	Ib	PRV1	Piscine orthoreovirus isolate 12-112_(59) F-Andoy 2012 Escaped segment S1 sigma 3 protein gene	Norway	2012	*Salmo salar*	Madhun
MG720341	12-115_(60)	Ib	PRV1	Piscine orthoreovirus isolate 12-115_(60) F-Andoy 2012 Escaped segment S1 sigma 3 protein gene	Norway	2012	*Salmo salar*	Madhun
MG720342	22-6_(9)	Ib	PRV1	Piscine orthoreovirus isolate 22-6_(9) B-Nordkapp 2012 Escaped segment S1 sigma 3 protein gene	Norway	2012	*Salmo salar*	Madhun
MG720343	12-142_(61)	Ib	PRV1	Piscine orthoreovirus isolate 12-142_(61) F-Andoy 2012 Escaped segment S1 sigma 3 protein gene	Norway	2012	*Salmo salar*	Madhun
MG720344	38-172_(44)	Ib	PRV1	Piscine orthoreovirus isolate 38-172_(44) D-Manes 2012 Wild segment S1 sigma 3 protein gene. partial	Norway	2012	*Salmo salar*	Madhun
MG720345	8-032_(62)	Ib	PRV1	Piscine orthoreovirus isolate 8-032_(62) A-Bugoynes 2012 Escaped segment S1 sigma 3 protein gene	Norway	2012	*Salmo salar*	Madhun
MG720346	38-242_(43)	Ib	PRV1	Piscine orthoreovirus isolate 38-242_(43) D-Manes 2012 Escaped segment S1 sigma 3 protein gene	Norway	2012	*Salmo salar*	Madhun
MG720347	38-197_(48)	Ib	PRV1	Piscine orthoreovirus isolate 38-197_(48) D-Manes 2012 Escaped segment S1 sigma 3 protein gene	Norway	2012	*Salmo salar*	Madhun
MG720348	31-60_(54)	Ib	PRV1	Piscine orthoreovirus isolate 31-60_(54) C-Alta 2012 Escaped segment S1 sigma 3 protein gene. partial	Norway	2012	*Salmo salar*	Madhun
MG720349	8-093_(63)	Ib	PRV1	Piscine orthoreovirus isolate 8-093_(63) A-Bugoynes 2012 Wild segment S1 sigma 3 protein gene. partial	Norway	2012	*Salmo salar*	Madhun
MG720350	38-267_(36)	Ib	PRV1	Piscine orthoreovirus isolate 38-267_(36) D-Manes 2012 Wild segment S1 sigma 3 protein gene. partial	Norway	2012	*Salmo salar*	Madhun
MG720351	38-382_(18)	Ib	PRV1	Piscine orthoreovirus isolate 38-382_(18) D-Manes 2012 Escaped segment S1 sigma 3 protein gene	Norway	2012	*Salmo salar*	Madhun
MG720352	38-369_(42)	Ib	PRV1	Piscine orthoreovirus isolate 38-369_(42) D-Manes 2012 Wild segment S1 sigma 3 protein gene. partial	Norway	2012	*Salmo salar*	Madhun
MG720353	38-226_(45)	Ib	PRV1	Piscine orthoreovirus isolate 38-226_(45) D-Manes 2012 Escaped segment S1 sigma 3 protein gene	Norway	2012	*Salmo salar*	Madhun
MG720354	38-268_(13)	Ib	PRV1	Piscine orthoreovirus isolate 38-268_(13) D-Manes 2012 Wild segment S1 sigma 3 protein gene. partial	Norway	2012	*Salmo salar*	Madhun
MG720355	12-503_(15)	Ib	PRV1	Piscine orthoreovirus isolate 12-503_(15) E-Stamnes 2012 Escaped segment S1 sigma 3 protein gene	Norway	2012	*Salmo salar*	Madhun
MG720356	12-20B_(55)	Ia	PRV1	Piscine orthoreovirus isolate 12-20B_(55) F-Andoy 2012 Escaped segment S1 sigma 3 protein gene	Norway	2012	*Salmo salar*	Madhun
MG720357	12-501_(16)	Ia	PRV1	Piscine orthoreovirus isolate 12-501_(16) E-Stamnes 2012 Escaped segment S1 sigma 3 protein gene	Norway	2012	*Salmo salar*	Madhun
MG720358	38-181_(41)	Ia	PRV1	Piscine orthoreovirus isolate 38-181_(41) D-Manes 2012 Escaped segment S1 sigma 3 protein gene	Norway	2012	*Salmo salar*	Madhun
MG720359	38-225_(40)	Ia	PRV1	Piscine orthoreovirus isolate 38-225_(40) D-Manes 2012 Escaped segment S1 sigma 3 protein gene	Norway	2012	*Salmo salar*	Madhun
MG720360	12-31_(14)	Ia	PRV1	Piscine orthoreovirus isolate 12-31_(14) E-Stamnes 2012 Wild segment S1 sigma 3 protein gene. partial	Norway	2012	*Salmo salar*	Madhun
MG720361	38-368_(37)	Ia	PRV1	Piscine orthoreovirus isolate 38-368_(37) D-Manes 2012 Wild segment S1 sigma 3 protein gene. partial	Norway	2012	*Salmo salar*	Madhun
MG720362	38-182_(39)	Ia	PRV1	Piscine orthoreovirus isolate 38-182_(39) D-Manes 2012 Escaped segment S1 sigma 3 protein gene	Norway	2012	*Salmo salar*	Madhun
MG720363	38-183_(12)	Ia	PRV1	Piscine orthoreovirus isolate 38-183_(12) D-Manes 2012 Escaped segment S1 sigma 3 protein gene	Norway	2012	*Salmo salar*	Madhun
MG720364	38-196_(34)	Ia	PRV1	Piscine orthoreovirus isolate 38-196_(34) D-Manes 2012 Escaped segment S1 sigma 3 protein gene	Norway	2012	*Salmo salar*	Madhun
MG720365	31-02_(53)	Ia	PRV1	Piscine orthoreovirus isolate 31-02_(53) C-Alta 2012 Escaped segment S1 sigma 3 protein gene. partial	Norway	2012	*Salmo salar*	Madhun
MG720366	12-35_(57)	Ia	PRV1	Piscine orthoreovirus isolate 12-35_(57) E-Stamnes 2012 Wild segment S1 sigma 3 protein gene. partial	Norway	2012	*Salmo salar*	Madhun
MG720367	8-017_(17)	Ia	PRV1	Piscine orthoreovirus isolate 8-017_(17) A-Bugoynes 2012 Wild segment S1 sigma 3 protein gene. partial	Norway	2012	*Salmo salar*	Madhun
MG720368	31-72_(7)	Ia	PRV1	Piscine orthoreovirus isolate 31-72_(7) C-Alta 2012 Wild segment S1 sigma 3 protein gene. partial cds	Norway	2012	*Salmo salar*	Madhun
MG720369	38-207_(35)	Ia	PRV1	Piscine orthoreovirus isolate 38-207_(35) D-Manes 2012 Escaped segment S1 sigma 3 protein gene	Norway	2012	*Salmo salar*	Madhun
MG720370	22-14_(8)	Ia	PRV1	Piscine orthoreovirus isolate 22-14_(8) B-Nordkapp 2012 Wild segment S1 sigma 3 protein gene. partial	Norway	2012	*Salmo salar*	Madhun
MG720371	38-363_(46)	Ia	PRV1	Piscine orthoreovirus isolate 38-363_(46) D-Manes 2012 Escaped segment S1 sigma 3 protein gene	Norway	2012	*Salmo salar*	Madhun
MG720372	38-367_(11)	Ia	PRV1	Piscine orthoreovirus isolate 38-367_(11) D-Manes 2012 Escaped segment S1 sigma 3 protein gene	Norway	2012	*Salmo salar*	Madhun
MG720373	38-185_(33)	Ia	PRV1	Piscine orthoreovirus isolate 38-185_(33) D-Manes 2012 Escaped segment S1 sigma 3 protein gene	Norway	2012	*Salmo salar*	Madhun
MG720374	38-269_(38)	Ia	PRV1	Piscine orthoreovirus isolate 38-269_(38) D-Manes 2012 Wild segment S1 sigma 3 protein gene. partial	Norway	2012	*Salmo salar*	Madhun
MG720375	38-176_(47)	Ib	PRV1	Piscine orthoreovirus isolate 38-176_(47) D-Manes 2012 Escaped segment S1 sigma 3 protein gene	Norway	2012	*Salmo salar*	Madhun
MG720376	22-24_(10)	Ib	PRV1	Piscine orthoreovirus isolate 22-24_(10) B-Nordkapp 2012 Escaped segment S1 sigma 3 protein gene	Norway	2012	*Salmo salar*	Madhun
MG720377	12-11_(56)	Ia	PRV1	Piscine orthoreovirus isolate 12-11_(56) E-Stamnes 2012 Wild segment S1 sigma 3 protein gene. partial	Norway	2012	*Salmo salar*	Madhun
MG770490	DH773072017	IIa	PRV3	Piscine orthoreovirus isolate PRV_Om_DH773/775_072017 sigma 3 protein gene. partial cds	Germany	2017	*Oncorhynchus mykiss*	Adamek
MG983780	NOR/060214	IIa	PRV3	Piscine orthoreovirus 3 isolate NOR/060214 sigma 3 protein gene. partial cds	Norway	2013	*Onchorynchus mykiss*	Dhamotharan
MG983781	G1491	IIa	PRV3	Piscine orthoreovirus 3 isolate G1491 sigma 3 protein gene. partial cds	Scotland	2017	*Onchorynchus mykiss*	Dhamotharan
MG983782	DK/17-18918-13	IIa	PRV3	Piscine orthoreovirus 3 isolate DK/17-18918-13 sigma 3 protein gene. partial cds	Denmark	2017	*Onchorynchus mykiss*	Dhamotharan
MG983783	IT/17-211.3	IIa	PRV3	Piscine orthoreovirus 3 isolate IT/17-19266-27 sigma 3 protein gene. partial cds	Italy	2017	*Salmo trutta*	Dhamotharan
MG983784	IT/17-267	IIa	PRV3	Piscine orthoreovirus 3 isolate IT/17-19266-35 sigma 3 protein gene. partial cds	Italy	2017	*Salmo trutta*	Dhamotharan
MG983785	DK/17-18918-1	IIa	PRV3	Piscine orthoreovirus 3 isolate DK/17-18918-1 sigma 3 protein gene. partial cds	Denmark	2017	*Onchorynchus mykiss*	Dhamotharan
MG983786	DK/17-18918-6	IIa	PRV3	Piscine orthoreovirus 3 isolate DK/17-18918-6 sigma 3 protein gene. partial cds	Denmark	2017	*Onchorynchus mykiss*	Dhamotharan
MG983787	DH/747072017	IIa	PRV3	Piscine orthoreovirus 3 isolate DH/747072017 sigma 3 protein gene. partial cds	Germany	2017	*Onchorynchus mykiss*	Dhamotharan
MH093952	A.3.2-36_G609	Ia	PRV1	Piscine orthoreovirus isolate A.3.2-36_G609 outer shell gene, complete cds	Canada	2011	*Salmo salar*	Di Cicco
MH093953	A.3.2-69_G531	Ia	PRV1	Piscine orthoreovirus isolate A.3.2-69_G531 outer shell gene, complete cds	Canada	2011	*Salmo salar*	Di Cicco
MH093954	A.3.2-153_G808	Ia	PRV1	Piscine orthoreovirus isolate A.3.2-153_G808 outer shell gene, complete cds	Canada	2013	*Salmo salar*	Di Cicco
MH093955	A.3.3-19_G744	Ia	PRV1	Piscine orthoreovirus isolate A.3.3-19_G744 outer shell gene, complete cds	Canada	2012	*Salmo salar*	Di Cicco
MH093956	A.3.3-133_G444	Ia	PRV1	Piscine orthoreovirus isolate A.3.3-133_G444 outer shell gene, complete cds	Canada	2012	*Salmo salar*	Di Cicco
MH093957	A.3.5-168_G860	Ia	PRV1	Piscine orthoreovirus isolate A.3.5-168_G860 outer shell gene, complete cds	Canada	2013	*Salmo salar*	Di Cicco
MH093958	P.2-1_G577	Ia	PRV1	Piscine orthoreovirus isolate P.2-1_G577 outer shell gene, complete cds	Canada	2011	*Oncorhynchus tshawytscha*	Di Cicco
MH093959	P.2-3_G460	Ia	PRV1	Piscine orthoreovirus isolate P.2-3_G460 outer shell gene, complete cds	Canada	2013	*Oncorhynchus tshawytscha*	Di Cicco
MH093960	P.2-95_G383	Ia	PRV1	Piscine orthoreovirus isolate P.2-95_G383 outer shell gene, complete cds	Canada	2013	*Oncorhynchus tshawytscha*	Di Cicco
MH093961	P.2-99_G772	Ia	PRV1	Piscine orthoreovirus isolate P.2-99_G772 outer shell gene, complete cds	Canada	2013	*Oncorhynchus tshawytscha*	Di Cicco
MH093962	P.3-3_G729	Ia	PRV1	Piscine orthoreovirus isolate P.3-3_G729 outer shell gene, complete cds	Canada	2012	*Oncorhynchus tshawytscha*	Di Cicco
MH093963	P.3-37_G446	Ia	PRV1	Piscine orthoreovirus isolate P.3-37_G446 outer shell gene, complete cds	Canada	2013	*Oncorhynchus tshawytscha*	Di Cicco
MH093964	P.3-120_G417	Ia	PRV1	Piscine orthoreovirus isolate P.3-120_G417 outer shell gene, complete cds	Canada	2012	*Oncorhynchus tshawytscha*	Di Cicco
MH093978	A.3.2-36_G609	Ia	PRV1	Piscine orthoreovirus isolate A.3.2-36_G609 outer clamp gene. partial cds	Canada	2011	*Salmo salar*	Di Cicco
MH093979	A.3.2-69_G531	Ia	PRV1	Piscine orthoreovirus isolate A.3.2-69_G531 outer clamp gene. partial cds	Canada	2011	*Salmo salar*	Di Cicco
MH093980	A.3.2-153_G808	Ia	PRV1	Piscine orthoreovirus isolate A.3.2-153_G808 outer clamp gene. partial cds	Canada	2013	*Salmo salar*	Di Cicco
MH093981	A.3.3-19_G744	Ia	PRV1	Piscine orthoreovirus isolate A.3.3-19_G744 outer clamp gene. partial cds	Canada	2012	*Salmo salar*	Di Cicco
MH093982	A.3.3-133_G444	Ia	PRV1	Piscine orthoreovirus isolate A.3.3-133_G444 outer clamp gene. partial cds	Canada	2012	*Salmo salar*	Di Cicco
MH093983	A.3.5-168_G860	Ia	PRV1	Piscine orthoreovirus isolate A.3.5-168_G860 outer clamp gene. partial cds	Canada	2013	*Salmo salar*	Di Cicco
MH093984	P.2-1_G577	Ia	PRV1	Piscine orthoreovirus isolate P.2-1_G577 outer clamp gene. partial cds	Canada	2011	*Oncorhynchus tshawytscha*	Di Cicco
MH093985	P.2-3_G460	Ia	PRV1	Piscine orthoreovirus isolate P.2-3_G460 outer clamp gene. partial cds	Canada	2013	*Oncorhynchus tshawytscha*	Di Cicco
MH093986	P.2-95_G383	Ia	PRV1	Piscine orthoreovirus isolate P.2-95_G383 outer clamp gene. partial cds	Canada	2013	*Oncorhynchus tshawytscha*	Di Cicco
MH093987	P.2-99_G772	Ia	PRV1	Piscine orthoreovirus isolate P.2-99_G772 outer clamp gene. partial cds	Canada	2013	*Oncorhynchus tshawytscha*	Di Cicco
MH093988	P.3-3_G729	Ia	PRV1	Piscine orthoreovirus isolate P.3-3_G729 outer clamp gene. partial cds	Canada	2012	*Oncorhynchus tshawytscha*	Di Cicco
MH093989	P.3-37_G446	Ia	PRV1	Piscine orthoreovirus isolate P.3-37_G446 outer clamp gene. partial cds	Canada	2013	*Oncorhynchus tshawytscha*	Di Cicco
MH093990	P.3-120_G417	Ia	PRV1	Piscine orthoreovirus isolate P.3-120_G417 outer clamp gene. partial cds	Canada	2012	*Oncorhynchus tshawytscha*	Di Cicco
MH215517	VT01192018-07	Ia	PRV1	Piscine orthoreovirus isolate VT01192018-07 sigma 3 protein and p13 genes. complete cds	USA	2017	*Salmo salar*	Kibenge
MH215518	VT01192018-16	Ia	PRV1	Piscine orthoreovirus isolate VT01192018-16 sigma 3 protein and p13 genes. complete cds	USA	2017	*Salmo salar*	Kibenge
MH215519	VT01192018-21	Ia	PRV1	Piscine orthoreovirus isolate VT01192018-21 sigma 3 protein and p13 genes. complete cds	USA	2017	*Salmo salar*	Kibenge
MH215520	VT01192018-26	Ia	PRV1	Piscine orthoreovirus isolate VT01192018-26 sigma 3 protein and p13 genes. complete cds	USA	2017	*Salmo salar*	Kibenge
MH215521	VT01192018-33	Ia	PRV1	Piscine orthoreovirus isolate VT01192018-33 sigma 3 protein and p13 genes. complete cds	USA	2017	*Salmo salar*	Kibenge
MH215522	VT01192018-43	Ia	PRV1	Piscine orthoreovirus isolate VT01192018-43 sigma 3 protein and p13 genes. complete cds	USA	2017	*Salmo salar*	Kibenge
MH215523	VT01192018-48	Ia	PRV1	Piscine orthoreovirus isolate VT01192018-48 sigma 3 protein and p13 genes. complete cds	USA	2017	*Salmo salar*	Kibenge
MH215524	VT01192018-52	Ia	PRV1	Piscine orthoreovirus isolate VT01192018-52 sigma 3 protein and p13 genes. complete cds	USA	2017	*Salmo salar*	Kibenge
MH215525	VT01192018-53	Ia	PRV1	Piscine orthoreovirus isolate VT01192018-53 sigma 3 protein and p13 genes. complete cds	USA	2017	*Salmo salar*	Kibenge
MH215526	VT10252017-405	Ia	PRV1	Piscine orthoreovirus isolate VT10252017-405 sigma 3 protein and p13 genes. complete cds	Canada	2017	*Salmo salar*	Kibenge
MH215527	VT11022017-423	Ia	PRV1	Piscine orthoreovirus isolate VT11022017-423 sigma 3 protein and p13 genes. complete cds	Canada	2017	*Salmo salar*	Kibenge
MH229780	ADLPRV3	IIa	PRV3	Piscine orthoreovirus 3 isolate ADLPRV3 segment M2, complete sequence	Chile	2017	*Oncorhynchus kisutch*	Bohle
MH229785	ADLPRV3	IIa	PRV3	Piscine orthoreovirus 3 isolate ADLPRV3 segment S1, complete sequence	Chile	2017	*Oncorhynchus kisutch*	Bohle
MH347363	16-005	unknown	unknown	Piscine orthoreovirus isolate 16-005 segment M2 outer capsid shell gene, complete cds.	Canada	2016	*Salmo salar*	Polinski
MH347364	16-005	unknown	unknown	Piscine orthoreovirus isolate 16-005 segment M2 outer capsid shell gene, complete cds	Canada	2016	*Salmo salar*	Polinski
MH347365	16-005	Ia	PRV1	Piscine orthoreovirus isolate 16-005 segment S1 outer capsid coat and nonstructural protein P13 genes, complete cds.	Canada	2016	*Salmo salar*	Polinski
MH347373	16-011	unknown	unknown	Piscine orthoreovirus isolate 16-011 segment M2 outer capsid shell gene, complete cds	Canada	2016	*Salmo salar*	Polinski
MH347375	16-011	Ia	PRV1	Piscine orthoreovirus isolate 16-011 segment S1 outer capsid coat and nonstructural protein P13 genes, complete cds.	Canada	2016	*Salmo salar*	Polinski
MH513870	Kuehn-PRV2	IIa	PRV3	Piscine orthoreovirus isolate S1 outer clamp protein gene. partial cds	Germany	2008	*Salmo salar*	Kuehn
MH558129	VT04202018-119	Ia	PRV1	Piscine orthoreovirus isolate VT04202018-119 segment S1 outer clamp protein and nonstructural protein	USA	2018	*Salmo salar*	Kibenge
MH558130	VT04202018-121	Ia	PRV1	Piscine orthoreovirus isolate VT04202018-121 segment S1 outer clamp protein and nonstructural protein	USA	2018	*Salmo salar*	Kibenge
MH558131	VT04202018-123	Ia	PRV1	Piscine orthoreovirus isolate VT04202018-123 segment S1 outer clamp protein and nonstructural protein	USA	2018	*Salmo salar*	Kibenge
MH558132	VT04202018-124	Ia	PRV1	Piscine orthoreovirus isolate VT04202018-124 segment S1 outer clamp protein and nonstructural protein	USA	2017	*Salmo salar*	Kibenge
MH558133	VT04202018-129	Ia	PRV1	Piscine orthoreovirus isolate VT04202018-129 segment S1 outer clamp protein and nonstructural protein	Iceland	2018	*Salmo salar*	Kibenge
MH581192	VT05252017-236	Ia	PRV1	Piscine orthoreovirus isolate VT05252017-236 outer clamp protein and non-structural protein genes	Canada	2017	*Salmo salar*	Kibenge
MH581193	VT06092017-347	Ia	PRV1	Piscine orthoreovirus isolate VT06092017-347 outer clamp protein and non-structural protein genes	Canada	2017	*Salmo salar*	Kibenge
MH581194	VT06092017-348	Ia	PRV1	Piscine orthoreovirus isolate VT06092017-348 outer clamp protein and non-structural protein genes	Canada	2017	*Salmo salar*	Kibenge
MH581195	VT10042017-382	Ia	PRV1	Piscine orthoreovirus isolate VT10042017-382 outer clamp protein and non-structural protein genes	Canada	2017	*Salmo salar*	Kibenge
MH581196	VT10042017-383	Ia	PRV1	Piscine orthoreovirus isolate VT10042017-383 outer clamp protein and non-structural protein genes	Canada	2017	*Salmo salar*	Kibenge
MH581197	VT10252017-421	Ia	PRV1	Piscine orthoreovirus isolate VT10252017-421 outer clamp protein and non-structural protein genes	Canada	2017	*Oncorhynchus tshawytscha*	Kibenge
MH581198	VT10252017-422	Ia	PRV1	Piscine orthoreovirus isolate VT10252017-422 outer clamp protein and non-structural protein genes	Canada	2017	*Oncorhynchus tshawytscha*	Kibenge
MH581199	VT11302017-453	Ia	PRV1	Piscine orthoreovirus isolate VT11302017-453 outer clamp protein and non-structural protein genes	Canada	2017	*Salmo salar*	Kibenge
MH581200	VT11302017-454	Ia	PRV1	Piscine orthoreovirus isolate VT11302017-454 outer clamp protein and non-structural protein genes	Canada	2017	*Salmo salar*	Kibenge
MH581201	VT03022018-66	Ia	PRV1	Piscine orthoreovirus isolate VT03022018-66 outer clamp protein and non-structural protein genes	Canada	2018	*Salmo salar*	Kibenge
MH581202	VT03022018-67	Ia	PRV1	Piscine orthoreovirus isolate VT03022018-67 outer clamp protein and non-structural protein genes	Canada	2018	*Salmo salar*	Kibenge
MH581203	VT03022018-68	Ia	PRV1	Piscine orthoreovirus isolate VT03022018-68 outer clamp protein and non-structural protein genes	Canada	2018	*Salmo salar*	Kibenge
MH581204	VT03262018-75-27	Ia	PRV1	Piscine orthoreovirus isolate VT03262018-75-27 outer clamp protein and non-structural protein genes	Canada	2018	*Salmo salar*	Kibenge
MH581205	VT03262018-77	Ia	PRV1	Piscine orthoreovirus isolate VT03262018-77 outer clamp protein and non-structural protein genes	Canada	2018	*Salmo salar*	Kibenge
MH581206	VT03262018-83	Ia	PRV1	Piscine orthoreovirus isolate VT03262018-83 outer clamp protein and non-structural protein genes	Canada	2018	*Salmo salar*	Kibenge

MH581207	VT03262018-84	Ia	PRV1	Piscine orthoreovirus isolate VT03262018-84 outer clamp protein and non-structural protein genes	Canada	2018	*Salmo salar*	Kibenge
MH581208	VT03262018-88	Ia	PRV1	Piscine orthoreovirus isolate VT03262018-88 outer clamp protein and non-structural protein genes	Canada	2018	*Salmo salar*	Kibenge
MH581209	VT04062018-105	Ia	PRV1	Piscine orthoreovirus isolate VT04062018-105 outer clamp protein and non-structural protein genes	Canada	2018	*Salmo salar*	Kibenge
MH581210	VT04062018-106	Ia	PRV1	Piscine orthoreovirus isolate VT04062018-106 outer clamp protein and non-structural protein genes	Canada	2018	*Salmo salar*	Kibenge
MH581211	VT04062018-108	Ia	PRV1	Piscine orthoreovirus isolate VT04062018-108 outer clamp protein and non-structural protein genes	Canada	2018	*Salmo salar*	Kibenge
MH581212	VT04062018-109	Ia	PRV1	Piscine orthoreovirus isolate VT04062018-109 outer clamp protein and non-structural protein genes	Canada	2018	*Salmo salar*	Kibenge
MK052922	13(P2)	IIa	PRV3	Piscine orthoreovirus isolate 13(P2) segment S1, complete sequence	Chile	2018	*Oncorhynchus kisutch*	Cartagena
MK052923	14(P2)	IIa	PRV3	Piscine orthoreovirus isolate 14(P2) segment S1, complete sequence	Chile	2018	*Oncorhynchus kisutch*	Cartagena
MK052924	15(P2)	IIa	PRV3	Piscine orthoreovirus isolate 15(P2) segment S1, complete sequence	Chile	2018	*Oncorhynchus kisutch*	Cartagena
MK052925	17(P5)	Ib	PRV1	Piscine orthoreovirus isolate 17(P5) segment S1, complete sequence.	Chile	2018	*Oncorhynchus kisutch*	Cartagena
MK052926	18(P5)	Ib	PRV1	Piscine orthoreovirus isolate 18(P5) segment S1, complete sequence.	Chile	2018	*Oncorhynchus kisutch*	Cartagena
MK052927	19(P2)	IIa	PRV3	Piscine orthoreovirus isolate 19(P2) segment S1, complete sequence.	Chile	2018	*Oncorhynchus kisutch*	Cartagena
MK052928	23(P2)	IIa	PRV3	Piscine orthoreovirus isolate 23(P2) segment S1, complete sequence	Chile	2018	*Oncorhynchus kisutch*	Cartagena
MK052929	25(P5)	Ib	PRV1	Piscine orthoreovirus isolate 25(P5) segment S1, complete sequence.	Chile	2018	*Oncorhynchus kisutch*	Cartagena
MK052930	27(P5)	Ib	PRV1	Piscine orthoreovirus isolate 27(P5) segment S1, complete sequence	Chile	2018	*Oncorhynchus kisutch*	Cartagena
MK052931	28(P5)	Ib	PRV1	Piscine orthoreovirus isolate 28(P5) segment S1, complete sequence.	Chile	2018	*Oncorhynchus kisutch*	Cartagena
MK286552	Kuehn-PRV2	IIa	PRV3	Piscine orthoreovirus outer shell protein gene, partial cds	Germany	2008	*Salmo trutta fario*	Kuehn
MK286554	Kuehn-PRV2	IIa	PRV3	Piscine orthoreovirus outer clamp protein gene. partial cds	Germany	2008	*Salmo trutta fario*	Kuehn
MK368744	DK-14-15958-4	Ia	PRV1	Piscine orthoreovirus isolate DK-14-15958-4 sigma 3 protein gene. complete cds	Denmark	2014	*Salmo salar*	Vendramin
MK368745	DK-2013-50-288-63	Ia	PRV1	Piscine orthoreovirus isolate DK-2013-50-288-63 sigma 3 protein gene. partial cds	Denmark	2013	*Salmo salar*	Vendramin
MK368746	DK-2013-50-259-10	Ia	PRV1	Piscine orthoreovirus isolate DK-2013-50-259-10 sigma 3 protein gene. partial cds	Denmark	2013	*Salmo salar*	Vendramin
MK368747	DK-2013-50-282-20	Ia	PRV1	Piscine orthoreovirus isolate DK-2013-50-282-20 sigma 3 protein gene. partial cds	Denmark	2013	*Salmo salar*	Vendramin
MK368748	DK-2013-50-282-11	Ia	PRV1	Piscine orthoreovirus isolate DK-2013-50-282-11 sigma 3 protein gene. partial cds	Denmark	2013	*Salmo salar*	Vendramin
MK368749	DK-14-16407-1	Ia	PRV1	Piscine orthoreovirus isolate DK-14-16407-1 sigma 3 protein gene. partial cds	Denmark	2014	*Salmo salar*	Vendramin
MK368750	DK-2013-50-288-65	Ia	PRV1	Piscine orthoreovirus isolate DK-2013-50-288-65 sigma 3 protein gene. partial cds	Denmark	2013	*Salmo salar*	Vendramin
MK368751	DK-15-23576-37	Ia	PRV1	Piscine orthoreovirus isolate DK-15-23576-37 sigma 3 protein gene. partial cds	Denmark	2015	*Salmo salar*	Vendramin
MK368752	DK-14-15550-2	Ib	PRV1	Piscine orthoreovirus isolate DK-14-15550-2 sigma 3 protein gene. partial cds	Denmark	2015	*Salmo salar*	Vendramin
MK368753	DK-14-16407-3	Ib	PRV1	Piscine orthoreovirus isolate DK-14-16407-3 sigma 3 protein gene. complete cds	Denmark	2014	*Salmo salar*	Vendramin
MK368754	DK-15-23478-20	Ib	PRV1	Piscine orthoreovirus isolate DK-15-23478-20 sigma 3 protein gene. partial cds	Denmark	2015	*Salmo salar*	Vendramin
MK368755	SW-17-1611-24	Ib	PRV1	Piscine orthoreovirus isolate SW-17-1611-24 sigma 3 protein gene. partial cds	Sweden	2017	*Salmo salar*	Vendramin
MK368756	IR-17-13316-6	Ia	PRV1	Piscine orthoreovirus isolate IR-17-13316-6 sigma 3 protein gene. partial cds	Ireland	2017	*Salmo salar*	Vendramin
MK368757	IR-17-6222-57	Ia	PRV1	Piscine orthoreovirus isolate IR-17-6222-57 sigma 3 protein gene. partial cds	Ireland	2017	*Salmo salar*	Vendramin
MK368758	F211-893-59	Ia	PRV1	Piscine orthoreovirus isolate F211-893-59 sigma 3 protein gene. partial cds	Faroe Islands	2011	*Salmo salar*	Vendramin
MK368759	F211-516-8	Ia	PRV1	Piscine orthoreovirus isolate F211-516-8 sigma 3 protein gene. partial cds	Faroe Islands	2011	*Salmo salar*	Vendramin
MK675826	NOR-1997	Ib	PRV1	Piscine orthoreovirus isolate NOR-1997 segment M2 mu1 gene, partial cds.	Norway	1997	*Salmo salar*	Dhamotharan
MK675828	NOR-1997	Ib	PRV1	Piscine orthoreovirus isolate NOR-1997 segment S1 sigma 3 and p13 genes. complete cds	Norway	1997	*Salmo salar*	Dhamotharan
MK675836	NOR-2005/TT	Ib	PRV1	Piscine orthoreovirus isolate NOR-2005/TT segment M2 mu1 gene, complete cds.	Norway	2005	*Salmo salar*	Dhamotharan
MK675838	NOR-2005/TT	Ib	PRV1	Piscine orthoreovirus isolate NOR-2005/TT segment S1 sigma 3 and p13 genes. complete cds	Norway	2005	*Salmo salar*	Dhamotharan
MK675846	NOR-2015/MS	Ib	PRV1	Piscine orthoreovirus isolate NOR-2015/MS segment M2 mu1 gene, complete cds	Norway	2015	*Salmo salar*	Dhamotharan
MK675848	NOR-2015/MS	Ib	PRV1	Piscine orthoreovirus isolate NOR-2015/MS segment S1 sigma 3 and p13 genes. complete cds	Norway	2015	*Salmo salar*	Dhamotharan
MK675856	NOR-2015/SSK	Ib	PRV1	Piscine orthoreovirus isolate NOR-2015/SSK segment M2 mu1 gene, complete cds	Norway	2015	*Salmo salar*	Dhamotharan
MK675858	NOR-2015/SSK	Ib	PRV1	Piscine orthoreovirus isolate NOR-2015/SSK segment S1 sigma 3 and p13 genes. complete cds	Norway	2015	*Salmo salar*	Dhamotharan
MK675866	NOR-1988	Ia	PRV1	Piscine orthoreovirus isolate NOR-1988 segment M2 mu1 gene, complete cds	Norway	1998	*Salmo salar*	Dhamotharan
MK675868	NOR-1988	Ia	PRV1	Piscine orthoreovirus isolate NOR-1988 segment S1 sigma 3 and p13 genes. complete cds	Norway	1998	*Salmo salar*	Dhamotharan
MK675876	FO/1978/15	Ia	PRV1	Piscine orthoreovirus isolate FO/1978/15 segment M2 mu1 gene, complete cds	Denmark	2015	*Salmo salar*	Vendramin
MK675878	FO/1978/15	Ia	PRV1	Piscine orthoreovirus isolate FO/1978/15 segment S1 sigma 3 and p13 genes. complete cds	Denmark	2015	*Salmo salar*	Vendramin
MK675886	FO/41/16	Ia	PRV1	Piscine orthoreovirus isolate FO/41/16 segment M2 mu1 gene, complete cds	Denmark	2016	*Salmo salar*	Vendramin
MK675888	FO/41/16	Ia	PRV1	Piscine orthoreovirus isolate FO/41/16 segment S1 sigma 3 and p13 genes. complete cds	Denmark	2016	*Salmo salar*	Vendramin
MN106287	J3542	unknown	unknown	Piscine orthoreovirus isolate J3542 L1 gene, complete cds.	Greenland	2017	*Salmo salar*	Teffer
MN106288	J3575	unknown	unknown	Piscine orthoreovirus isolate J3575 L1 gene, complete cds.	Greenland	2017	*Salmo salar*	Teffer
MN106289	J3611	unknown	unknown	Piscine orthoreovirus isolate J3611 L1 gene, complete cds.	Greenland	2016	*Salmo salar*	Teffer
MN106290	J3542	unknown	unknown	Piscine orthoreovirus isolate J3542 L2 gene, complete cds.	Greenland	2017	*Salmo salar*	Teffer
MN106291	J3575	unknown	unknown	Piscine orthoreovirus isolate J3575 L2 gene, complete cds.	Greenland	2017	*Salmo salar*	Teffer
MN106292	J3611	unknown	unknown	Piscine orthoreovirus isolate J3611 L2 gene, complete cds.	Greenland	2016	*Salmo salar*	Teffer
MN106293	J3542	unknown	unknown	Piscine orthoreovirus isolate J3542 L3 gene, complete cds.	Greenland	2017	*Salmo salar*	Teffer
MN106294	J3575	unknown	unknown	Piscine orthoreovirus isolate J3575 L3 gene, complete cds.	Greenland	2017	*Salmo salar*	Teffer
MN106295	J3611	unknown	unknown	Piscine orthoreovirus isolate J3611 L3 gene, complete cds.	Greenland	2016	*Salmo salar*	Teffer
MN106296	J3542	unknown	unknown	Piscine orthoreovirus isolate J3542 M1 gene, complete cds.	Greenland	2017	*Salmo salar*	Teffer
MN106297	J3575	unknown	unknown	Piscine orthoreovirus isolate J3575 M1 gene, complete cds.	Greenland	2017	*Salmo salar*	Teffer
MN106298	J3611	unknown	unknown	Piscine orthoreovirus isolate J3611 M1 gene, complete cds.	Greenland	2016	*Salmo salar*	Teffer
MN106299	J3542	unknown	unknown	Piscine orthoreovirus isolate J3542 M2 gene, complete cds.	Greenland	2017	*Salmo salar*	Teffer
MN106300	J3575	unknown	unknown	Piscine orthoreovirus isolate J3575 M2 gene, complete cds.	Greenland	2017	*Salmo salar*	Teffer
MN106301	J3611	unknown	unknown	Piscine orthoreovirus isolate J3611 M2 gene, complete cds.	Greenland	2016	*Salmo salar*	Teffer
MN106302	J3542	unknown	unknown	Piscine orthoreovirus isolate J3542 M3 gene, complete cds.	Greenland	2017	*Salmo salar*	Teffer
MN106303	J3575	unknown	unknown	Piscine orthoreovirus isolate J3575 M3 gene, complete cds.	Greenland	2017	*Salmo salar*	Teffer
MN106304	J3611	unknown	unknown	Piscine orthoreovirus isolate J3611 M3 gene, complete cds.	Greenland	2016	*Salmo salar*	Teffer
MN106305	J3542	unknown	unknown	Piscine orthoreovirus isolate J3542 S1 gene, complete cds.	Greenland	2017	*Salmo salar*	Teffer
MN106306	J3575	unknown	unknown	Piscine orthoreovirus isolate J3575 S1 gene, complete cds.	Greenland	2017	*Salmo salar*	Teffer
MN106307	J3611	unknown	unknown	Piscine orthoreovirus isolate J3611 S1 gene, complete cds.	Greenland	2016	*Salmo salar*	Teffer
MN106308	J3542	unknown	unknown	Piscine orthoreovirus isolate J3542 S2 gene, complete cds.	Greenland	2017	*Salmo salar*	Teffer
MN106309	J3575	unknown	unknown	Piscine orthoreovirus isolate J3575 S2 gene, complete cds.	Greenland	2017	*Salmo salar*	Teffer
MN106310	J3611	unknown	unknown	Piscine orthoreovirus isolate J3611 S2 gene, complete cds.	Greenland	2016	*Salmo salar*	Teffer
MN106311	J3542	unknown	unknown	Piscine orthoreovirus isolate J3542 S3 gene, complete cds.	Greenland	2017	*Salmo salar*	Teffer
MN106312	j3575	unknown	unknown	Piscine orthoreovirus isolate J3575 S3 gene, complete cds.	Greenland	2017	*Salmo salar*	Teffer
MN106313	J3611	unknown	unknown	Piscine orthoreovirus isolate J3611 S3 gene, complete cds.	Greenland	2016	*Salmo salar*	Teffer
MN106314	J3542	unknown	unknown	Piscine orthoreovirus isolate J3542 S4 gene, partial cds	Greenland	2018	*Salmo salar*	Teffer
MN106315	J3575	unknown	unknown	Piscine orthoreovirus isolate J3575 S4 gene, partial cds	Greenland	2017	*Salmo salar*	Teffer
MN106316	J3611	unknown	unknown	Piscine orthoreovirus isolate J3611 S4 gene, partial cds	Greenland	2016	*Salmo salar*	Teffer
MN337614	2019-50-212	unknown	unknown	Piscine orthoreovirus 3 isolate 2019-50-212 S3 gene, partial cds.	Norway	2017	*Onchorynchus mykiss*	Moldal
NC036472	CGA280-05	Ib	PRV1	Piscine reovirus isolate CGA280-05 outer clamp protein and non-structural protein gene, segment S1, complete cds.	Chile	2011	*Salmo salar*	Kibenge
