# Supplementary figures and images for: Extensive Phylogenetic Analysis of Piscine Orthoreovirus Genomic Sequences Shows the Robustness of Subgenotype Classification
Source: Pathogens. 2021 Jan 7;10(1):41. doi: 10.3390/pathogens10010041 (PMC7825714; doi:10.3390/pathogens10010041)

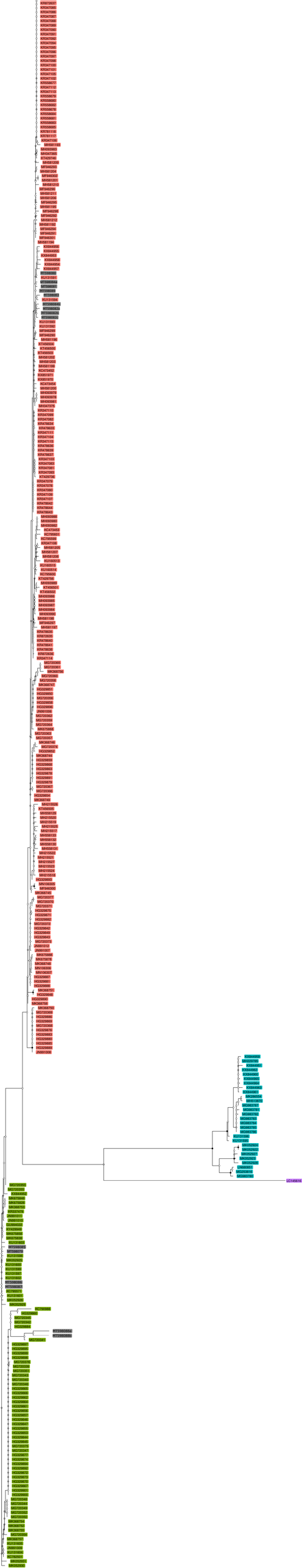

factor(genotype)

|   |               |
|---|---------------|
| a | la            |
| b | lb            |
| a | lla           |
| a | llb           |
| a | New sequences |

Bootstrap support (BP)

|   |                |
|---|----------------|
| ● | BP ≥ 0.9       |
| ○ | 0.7 ≤ BP < 0.9 |
| ○ | BP < 0.7       |

Supplement: Supplementary file 1 [file pathogens-10-00041-s001.zip › Supplementary folder/pathogens_Supplementary Figure S1_December 13 2020.pdf]

L1 segment

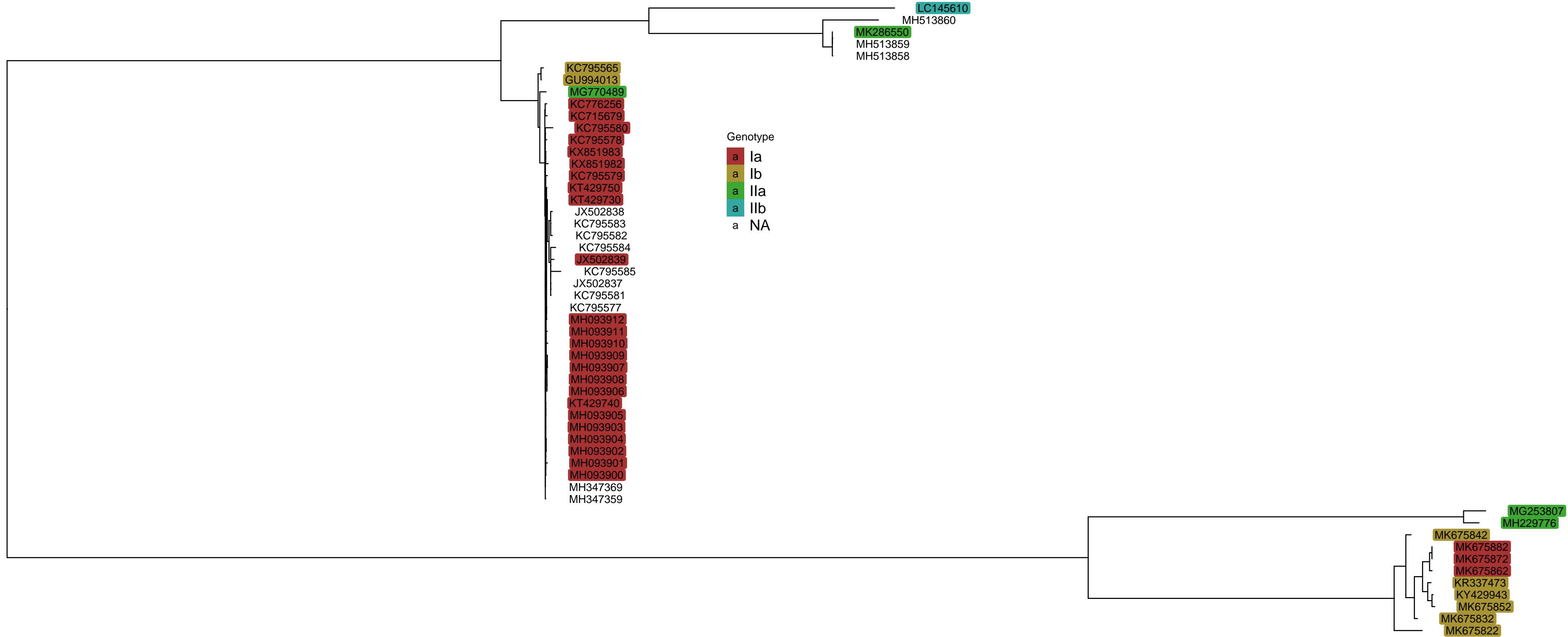

L2 segment

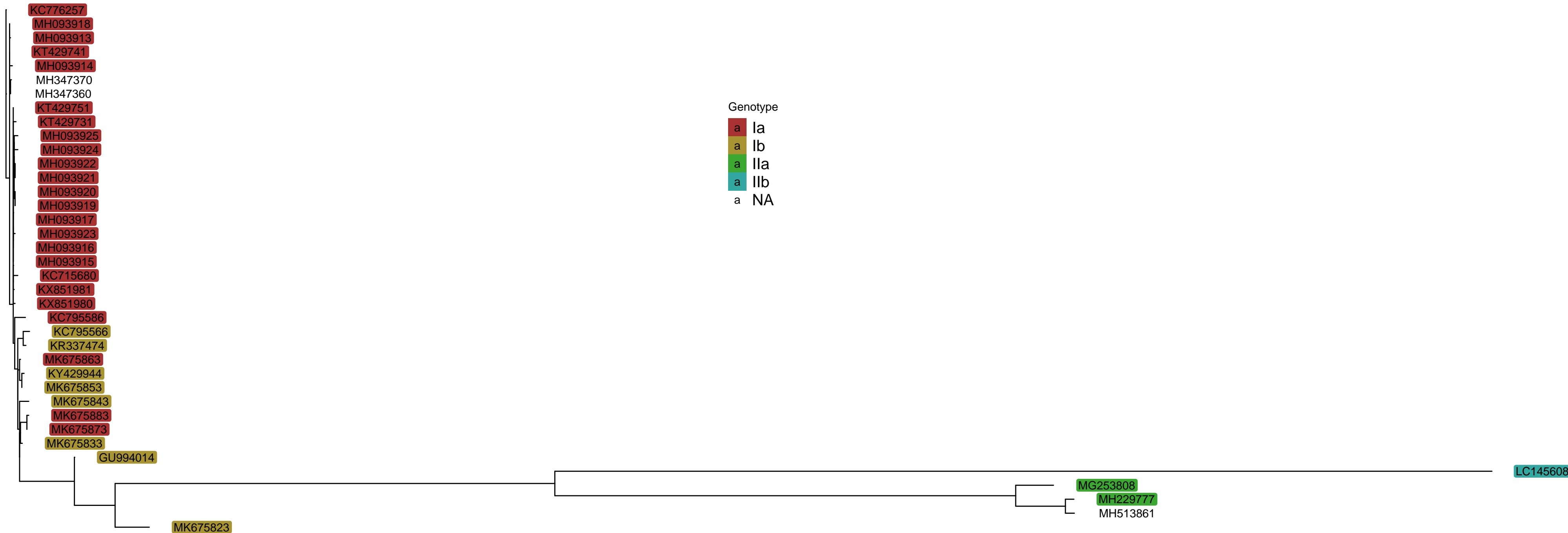

L3 segment

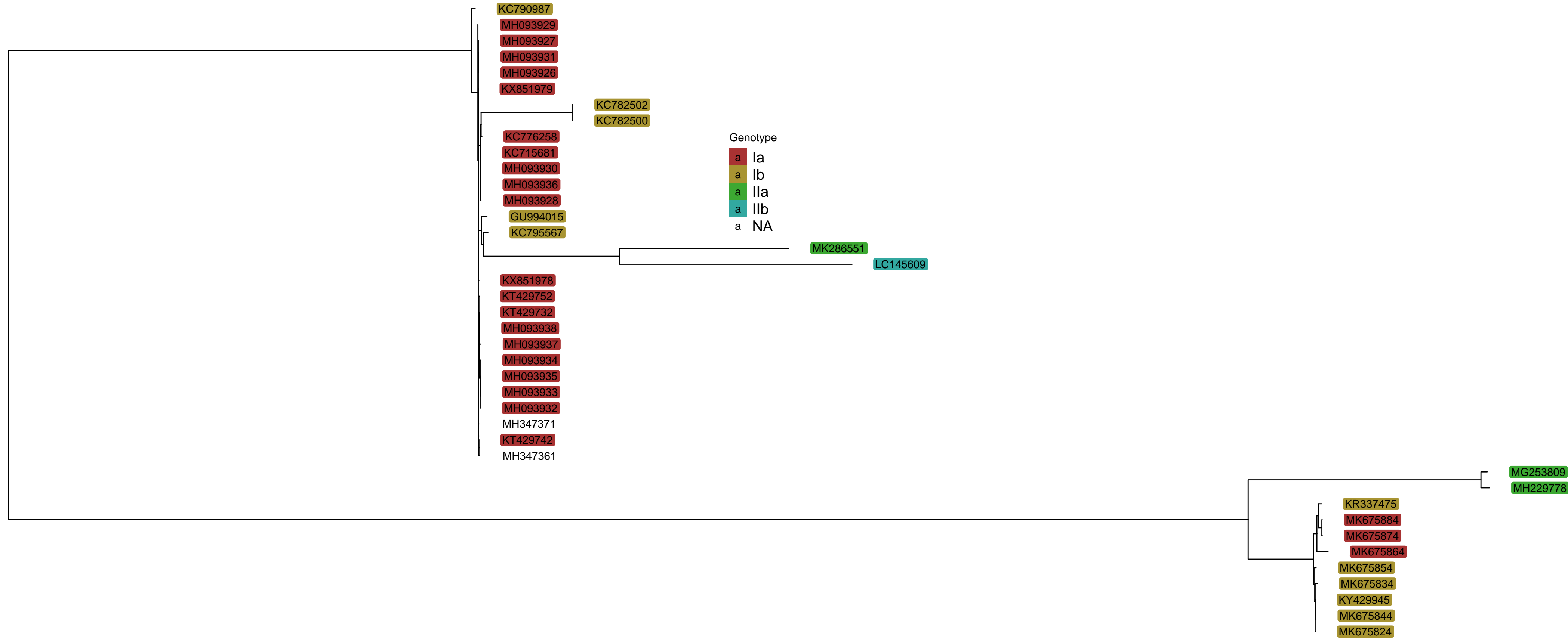

M1 segment

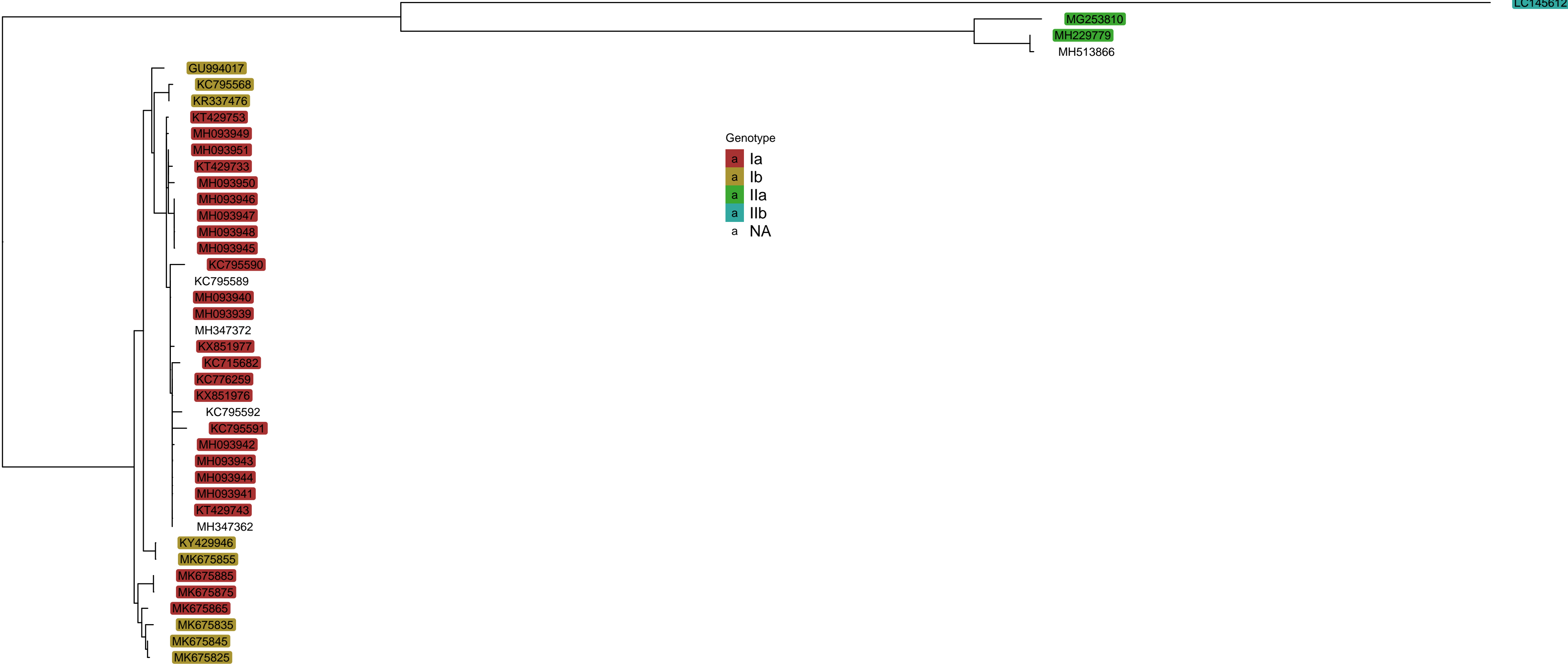

M3 segment

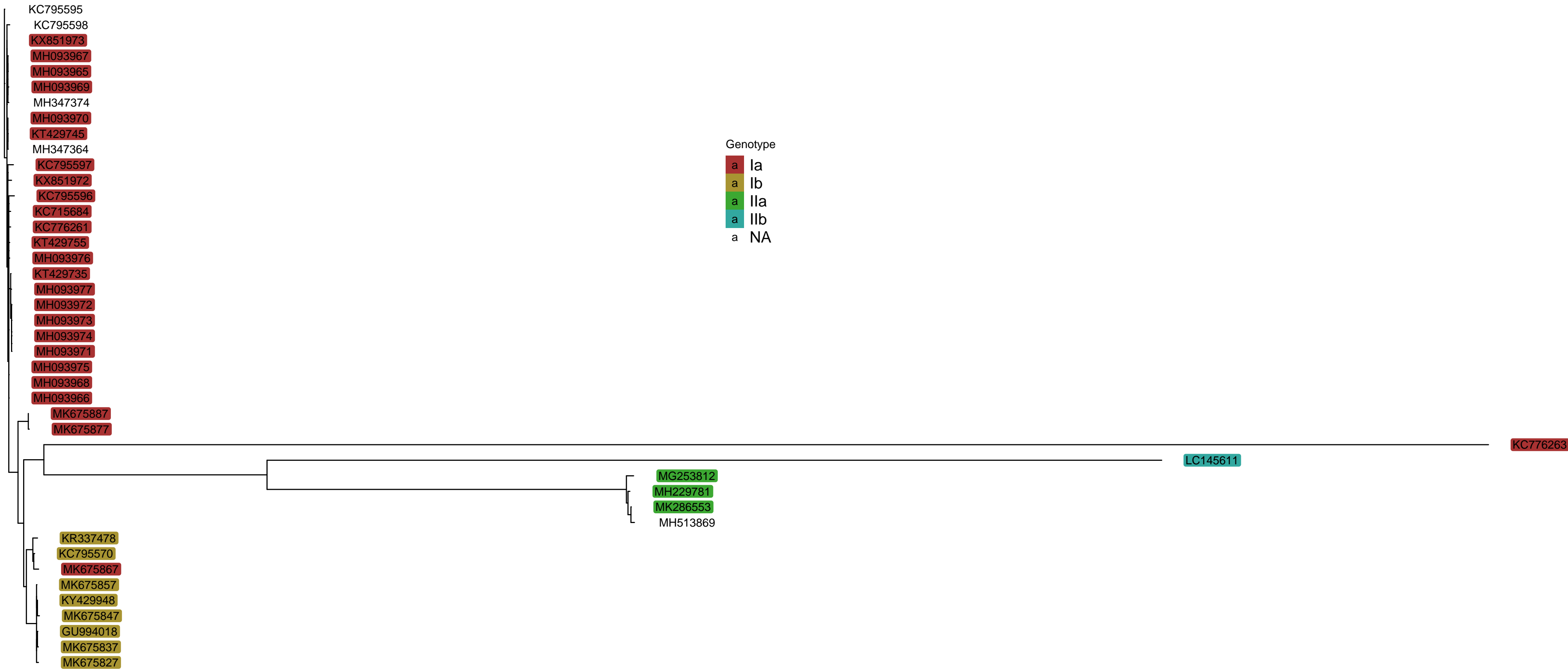

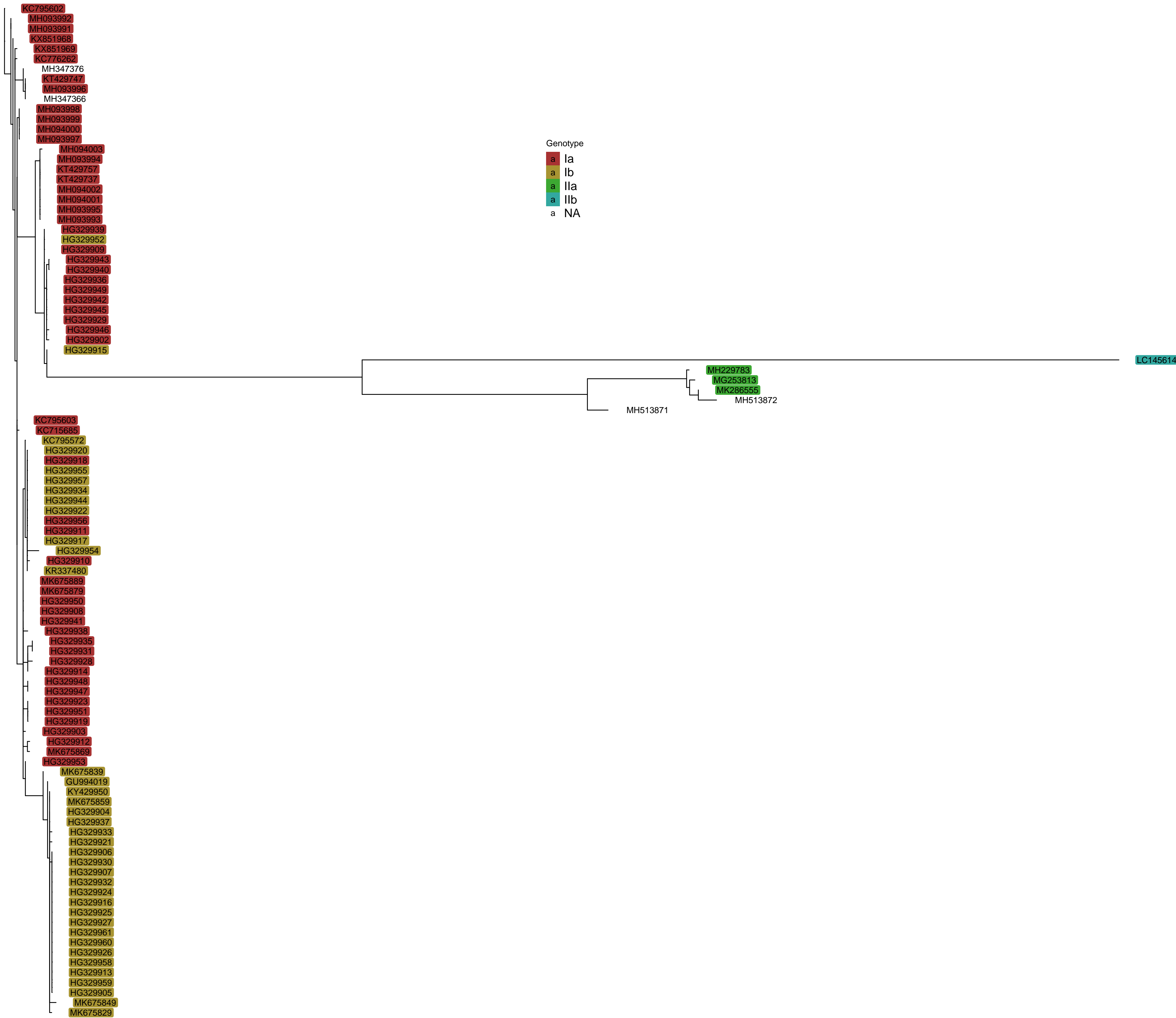

S3 segment

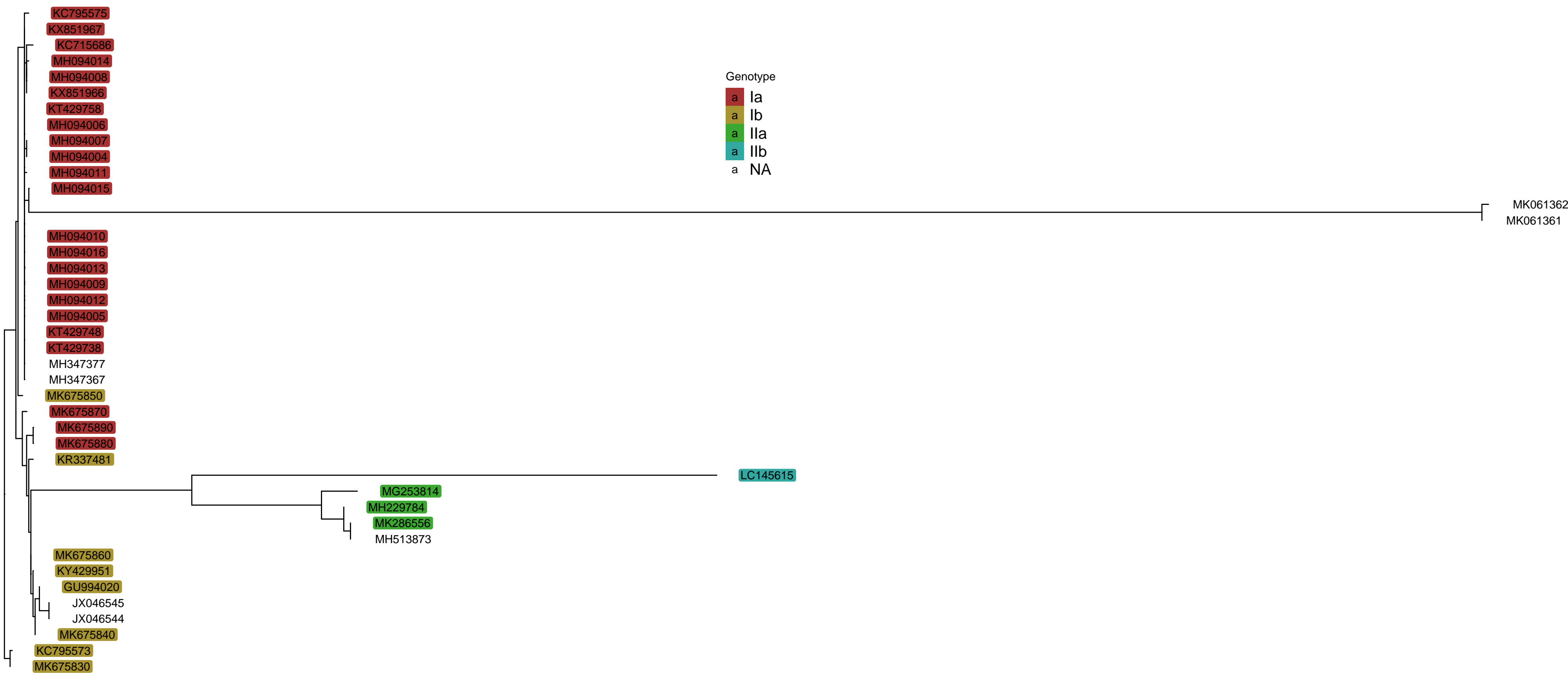

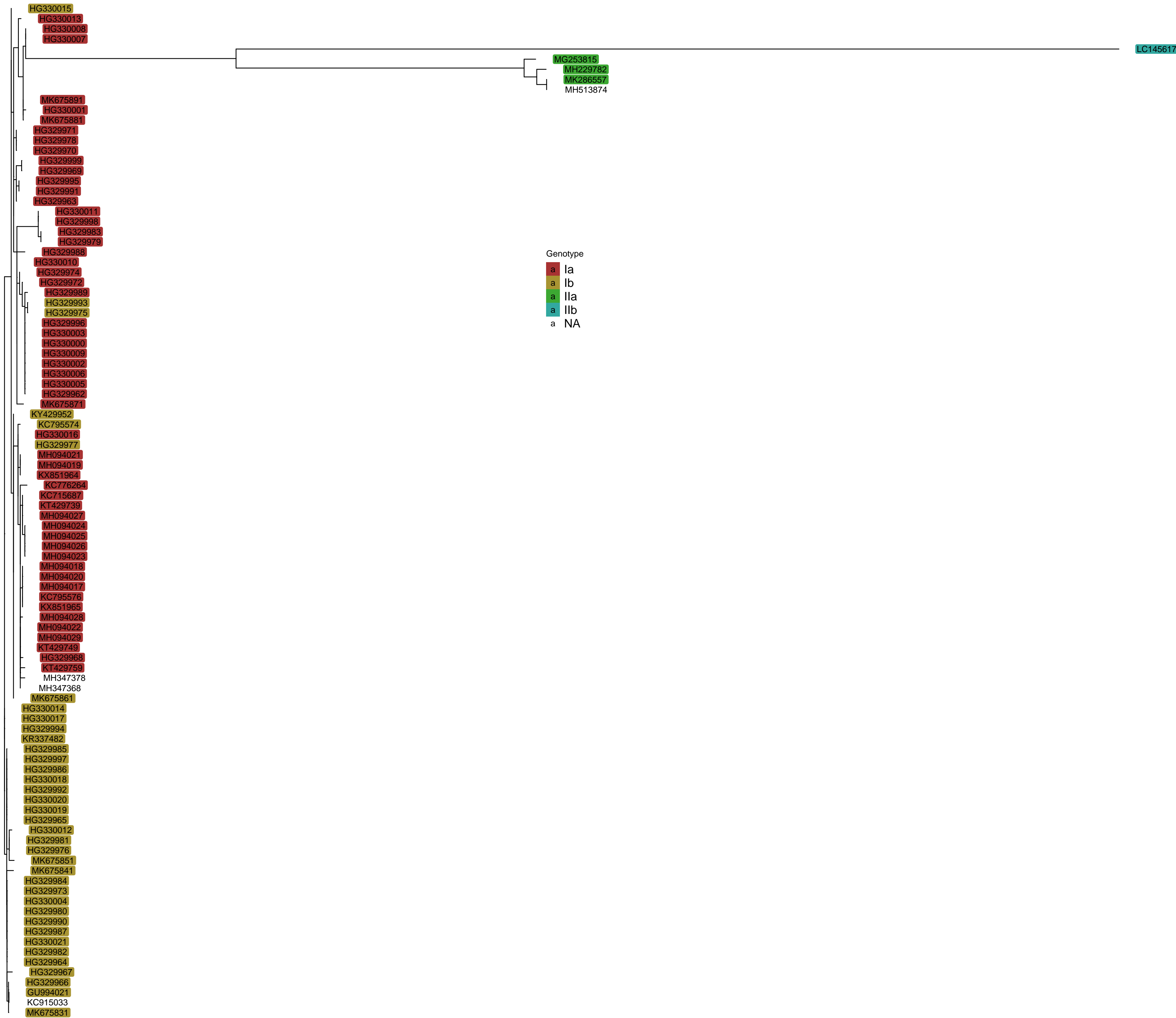

Supplement: Supplementary file 1 [file pathogens-10-00041-s001.zip › Supplementary folder/pathogens_Supplementary Figure S2_December 13 2020.pdf]

### Mu 1 Matrix Distance

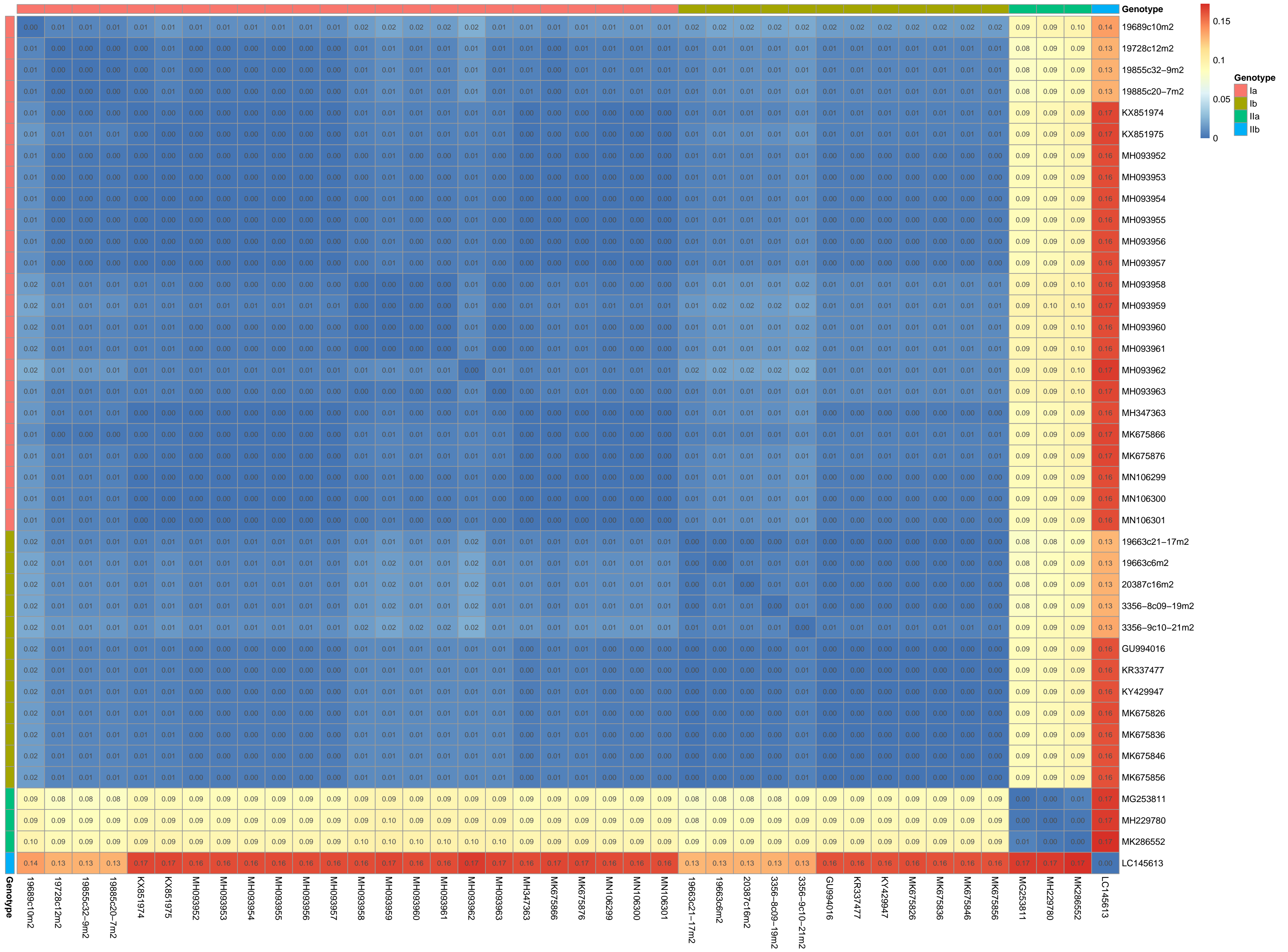

Supplement: Supplementary file 1 [file pathogens-10-00041-s001.zip › Supplementary folder/pathogens_Supplementary Figure S3_January 04 2021.pdf]

### Sigma 3 Matrix Distance

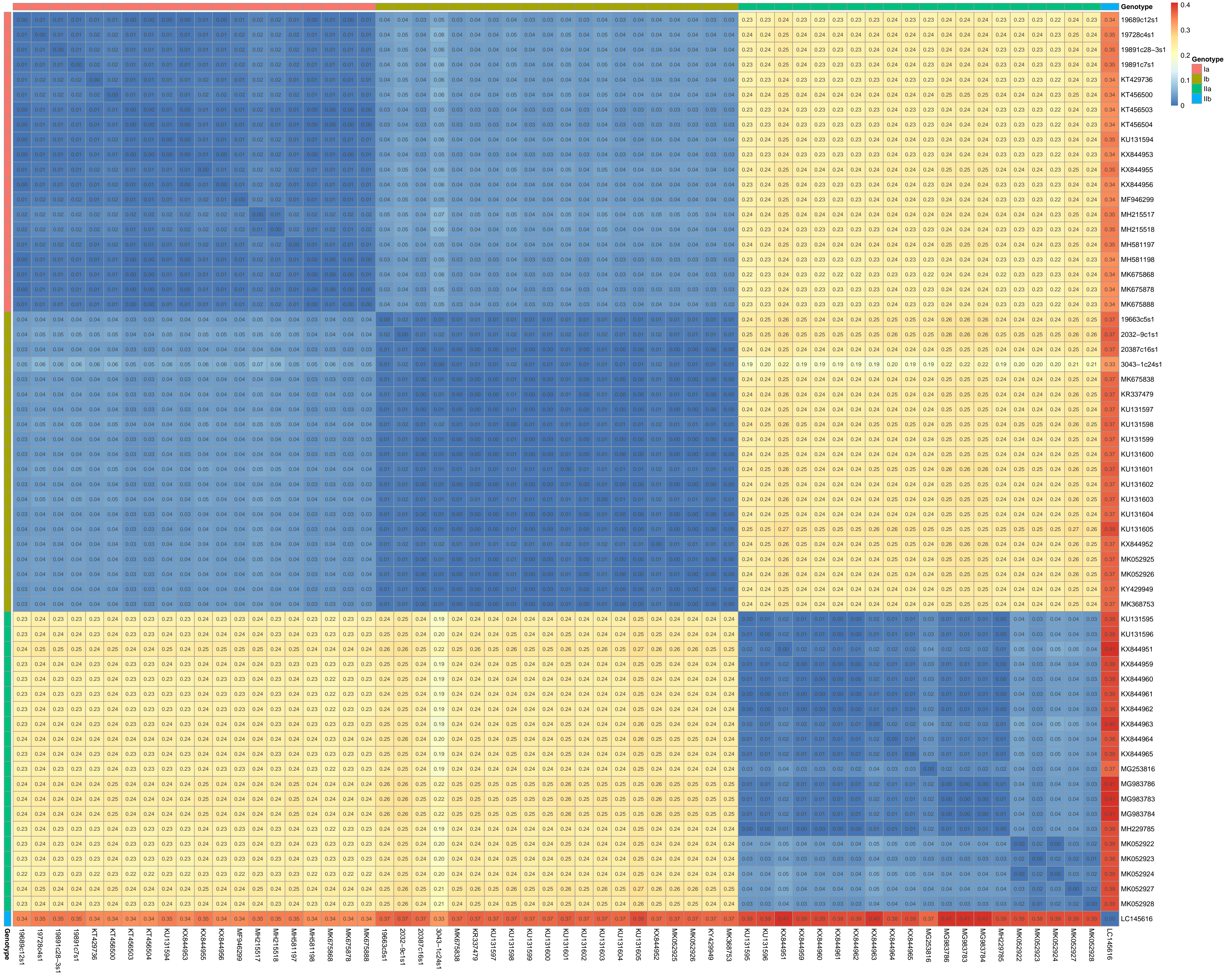

Supplement: Supplementary file 1 [file pathogens-10-00041-s001.zip › Supplementary folder/pathogens_Supplementary Figure S4_January 04 2021.pdf]

### Sigma 3 Matrix Di

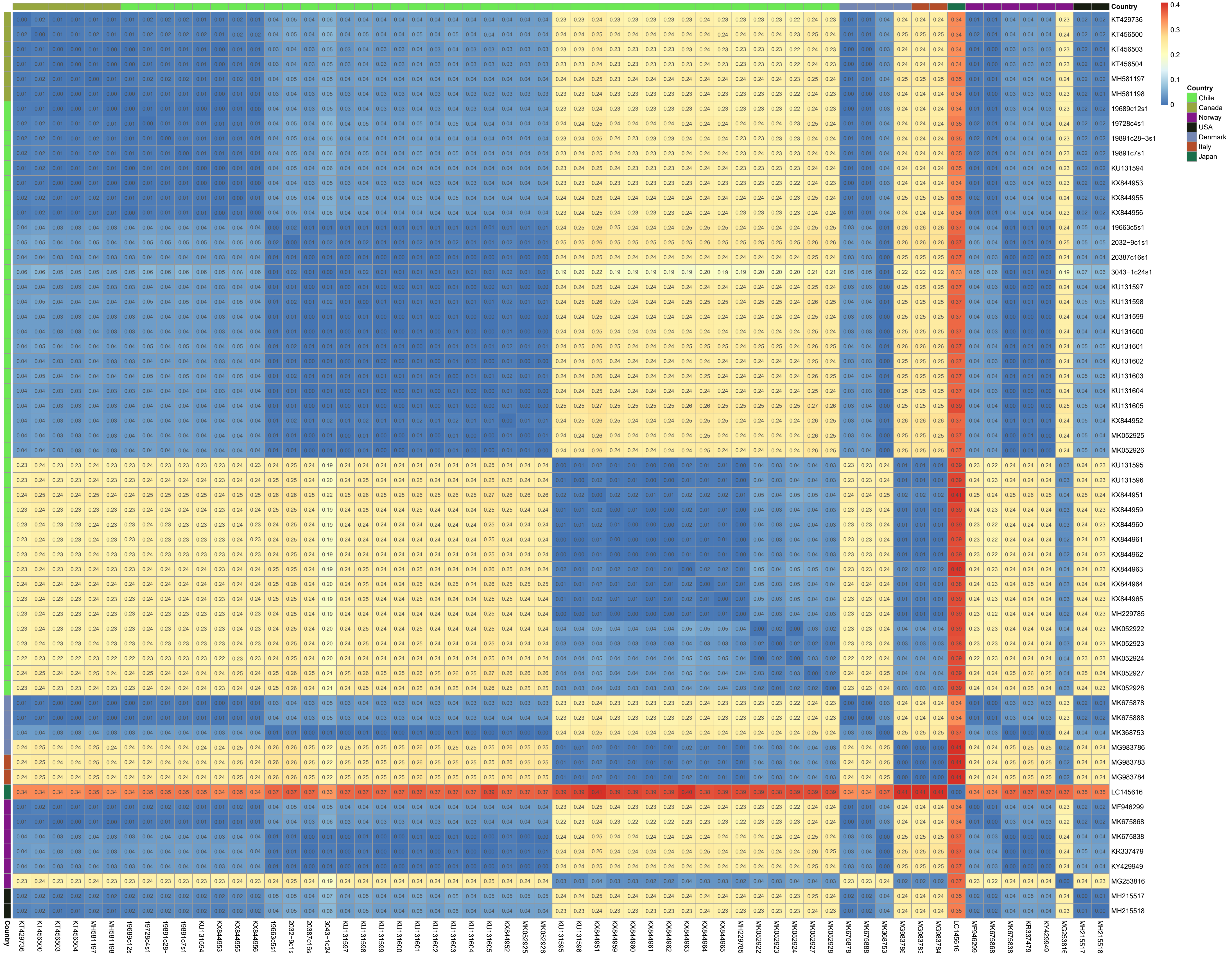

Supplement: Supplementary file 1 [file pathogens-10-00041-s001.zip › Supplementary folder/pathogens_Supplementary Figure S5_January 04 2021.pdf]
